# Anchoring of perforin-2 via the transmembrane domain is required for antigen escape into the cytosol

**DOI:** 10.1038/s44318-026-00903-z

**Published:** 2026-08-19

**Authors:** Marco Laub, Ritika Chatterjee, Patrycja Kozik

**Affiliations:** MRC Laboratory of Molecular Biology, Cambridge, UK

**Keywords:** Immunology, Membranes & Trafficking, Structural Biology

## Abstract

Perforin-2 is a pore-forming protein localised to the endocytic compartments of dendritic cells and macrophages. It is reported to perform two distinct functions during immune responses: attacking intravacuolar pathogens, and forming pores in endocytic compartments to enable cytosolic delivery of antigens during cross-presentation. The molecular mechanisms that regulate perforin-2 remain unknown. Here, we address how cross-presenting dendritic cells control pore formation in phagosomes while maintaining the integrity of their endocytic compartments. We demonstrate that perforin-2 undergoes extensive proteolytic processing involving multiple endocytic proteases. Although the transmembrane anchor has been proposed to protect host membranes by orienting pores towards bacterial targets, we find that endocytic escape is mediated by full-length, membrane-anchored perforin-2 rather than by the proteolytically released ectodomain. Moreover, we show that perforin-2-mediated antigen translocation does not require low pH, explaining how perforin-2 can form pores in cross-presenting dendritic cells which do not acidify their phagosomes. Our findings point to a critical role of the transmembrane anchor in perforin-2 biology and suggest that perforin-2 employs distinct mechanisms of pore formation during anti-bacterial defence and cross-presentation.

## Synopsis

Perforin-2 forms pores with functions in anti-bacterial immunity and in antigen delivery into the cytosol during cross-presentation. This study shows that perforin-2 undergoes extensive proteolytic processing, but its full-length form mediates antigen trafficking in dendritic cells.

- Perforin-2 undergoes extensive proteolytic processing, including ectodomain shedding.
- Proteolytic maturation of perforin-2 is mediated by a range of proteases, including cathepsins, AEP, and γ-secretase.
- Proteolytic processing of perforin-2 takes place primarily in low-pH compartments.
- Endocytic escape of antigens in dendritic cells is mediated by full-length, transmembrane-anchored perforin-2, and does not require low pH.

## Abstract (web summary)

Perforin-2 employs distinct mechanisms of pore formation during anti-bacterial defence and antigen cross-presentation.

## Graphical abstract

**Figure:**
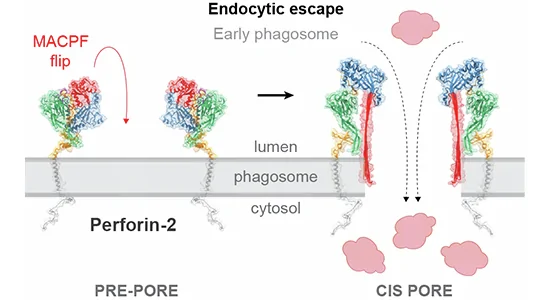

## Introduction

The mammalian immune system employs a family of membrane attack complex and perforin (MACPF) domain-containing pore-forming proteins in innate and adaptive immunity. The family includes C6-C9 subunits of the complement membrane attack complex as well as two perforins. Complement components are produced primarily by hepatocytes and secreted into the serum, where they can form lytic pores on the surface of Gram-negative bacteria, enveloped viruses, parasites, and host cells. Perforin-1 (*Prf1*) is expressed in cytotoxic T cells and natural killer cells. Upon activation, perforin-1-containing cytotoxic granules are secreted into the immune synapse between the killer cell and the target. Perforin-1 pores can be lytic at high concentration, but their primary role is to deliver apoptosis-inducing granzymes into the cytosol of the target cell (reviewed in (Krawczyk et al, 2020)). Perforin-2 (*Mpeg1*) is the evolutionarily most ancient member of the mammalian MACPF family (McCormack and Podack, 2015), yet it remains the least well characterised. Perforin-2 is highly expressed by antigen presenting cells such as dendritic cells and macrophages, as well as by neutrophils. Initially, it has been proposed to target intravacuolar bacteria (Fields et al, 2013; McCormack et al, 2015), but its role in anti-bacterial immunity has been questioned in later work (Ebrahimnezhaddarzi et al, 2022). More recently, we demonstrated that perforin-2 can form pores in the membranes of endocytic compartments to facilitate the release of internalised antigens into the cytosol, where they are processed by the proteasomes (Rodríguez-Silvestre et al, 2023). Proteasome-derived peptides are then presented on MHC class I molecules, enabling APCs to activate antigen-specific T cells even when they do not express the antigens themselves, in a pathway referred to as cross-presentation.

**Figure 1:**
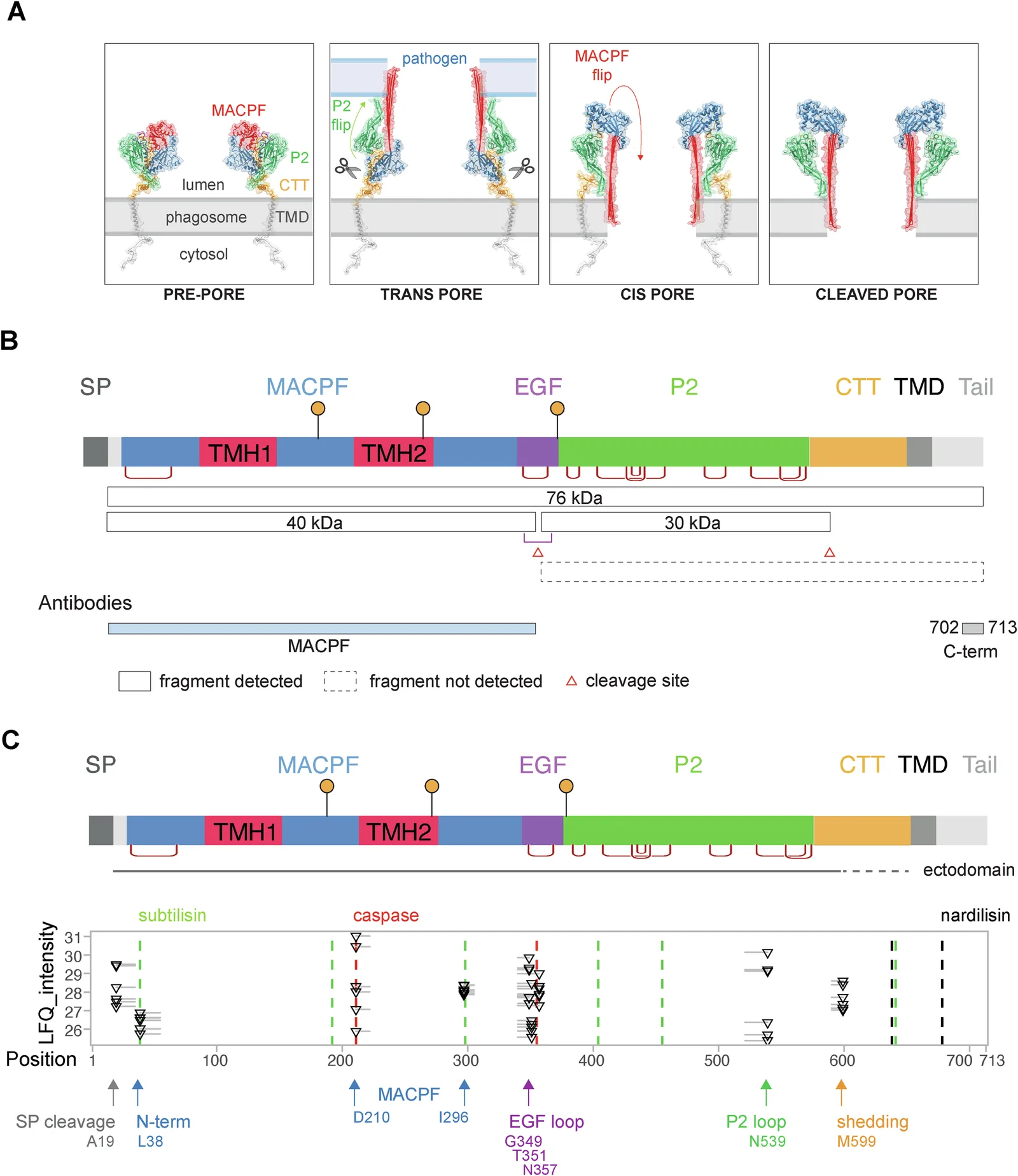
Mapping of perforin-2 cleavage sites by proteomics. (**A**) Schematic of the pre-, trans, cis and cleaved pores of perforin-2. (**B**) Schematic illustrating hypothetical (dashed lines) and previously observed (solid lines) perforin-2 fragments, including epitopes for perforin-2 antibodies. (**C**) Non-tryptic peptides identified by mass-spectrometry of total cell lysates of MutuDCs. The triangles indicate non-tryptic cleavage sites. Vertical dotted lines indicate cleavage sites predicted by the ELM database. See also Dataset EV1.

The mechanism of pore formation by MACPF-containing proteins is broadly conserved and shared with a related family of cholesterol-dependent cytolysins (CDCs) present in bacteria (Dunstone and Tweten, 2012). The key steps of this process include (1) association with the membrane, (2) oligomerisation into a pre-pore structure and (3) transition from the pre-pore to pore conformation through unwinding of the α-helical transmembrane hairpins (TMH1 and TMH2). Following the conformational change, each TMH contributes two β-sheets to the resulting β-barrel pore (Serna et al, 2016; Law et al, 2010; Ivanova et al, 2022; Pang et al, 2019; Ni et al, 2020). Distinct from other proteins in the MACPF/CDC superfamily, perforin-2 has an additional transmembrane domain (TMD) which does not form part of the pore (Pang et al, 2019; Ni et al, 2020) (Fig. 1A,B). As a consequence of this membrane anchoring, in the oligomeric pre-pore state of perforin-2, the pore-forming TMH helices point away from the membrane (Fig. 1A). Thus, it has been initially proposed that the TMD serves to protect endogenous membranes by orienting the pores towards the lumen of a pathogen-containing vacuole (Pang et al, 2019). However, perforin-2’s function in endocytic escape of antigens in cross-presenting DCs suggests that pore formation in endogenous membranes is an important function of perforin-2, rather than an unwanted effect (Rodríguez-Silvestre et al, 2023).

If TMHs face into the organellar lumen, how are perforin-2 pores inserted into the membranes during cross-presentation? One solution to this topological problem is to proteolytically release perforin-2’s ectodomain from the membrane. Notably, the structures of perforin-2 pores have been obtained using the ectodomain only, suggesting, at least in vitro, perforin-2 can undergo pre-pore to pore transition without the TMD (Pang et al, 2019; Ni et al, 2020; Yu et al, 2022). Indeed, in the cDC1-like cell line, MutuDCs (Marraco et al, 2012), the majority of perforin-2 has lost the TMD (Rodríguez-Silvestre et al, 2023; Ebrahimnezhaddarzi et al, 2022). Such proteolytic release would enable rotation of the TMHs towards endogenous membranes. However, in the absence of the TMD, perforin-2 binds membranes via the tip of the P2 domain, which in the pre-pore points in the opposite direction to the TMHs (see Fig. 1). Thus, the proteolytic release alone does not explain how the pores can form on the same membrane on which the pre-pore sits.

Interestingly, two models have been proposed for the pre-pore-to-pore conformational transition of perforin-2. The model where the TMHs unfurl away from the membrane and insert into an opposing bilayer is referred to as the trans model (Fig. 1A). Whether formation of the trans pores is preceded by the rotation of the P2 domain has not been demonstrated, but it appears likely, as it would facilitate binding to (pore insertion into) the target membrane (Pang et al, 2019; Ni et al, 2020). This mode of pore formation would allow for targeting intravacuolar pathogens, but, as discussed above, is unlikely to facilitate the release of endocytic cargo. In the alternative cis model, the P2 domain remains bound to the membrane, but the MACPF domain rotates by 180 degrees prior to unfurling (Fig. 1A) (Jiao et al, 2022; Yu et al, 2022). This allows for the insertion of pores into the membrane on which the pre-pore sits. The cis model could be employed during pore formation on bacterial membranes, but this would require proteolytic release of the ectodomain to enable pre-pores to bind the pathogen surface. Importantly, this model would also enable the formation of pores in the membranes of endocytic compartments. Theoretically, the TMD would not need to be removed to enable endocytic escape via cis pores, but whether full-length perforin-2 can oligomerise and make pores has not been demonstrated.

Here, we asked whether ectodomain shedding is required for perforin-2-mediated endocytic escape. To this end, we first mapped the cleavage sites involved in perforin-2 maturation and identified the proteases involved. This enabled us to inhibit proteolytic maturation of perforin-2. Surprisingly, we found that shedding was not required for pore formation. Instead, we demonstrate that membrane anchoring of perforin-2 facilitates formation of endocytic escape pores, and mutations in the CTT domain, which links the ectodomain with the TMD, interfere with escape. Together, these data point to a central role of the TMD anchor during perforin-2-mediated antigen escape and indicate that ectodomain shedding could serve as a mechanism to inactivate perforin-2 in dendritic cells.

## Results

### Mapping of perforin-2 cleavage sites by proteomics

The perforin-2 ectodomain has a predicted molecular weight (MW) of approximately 76 kDa and is composed of the pore-forming MACPF domain, an epidermal growth factor (EGF) linker, a membrane binding P2 domain, and an unstructured CTT domain that connects it to the TMD (Fig. 1B). At steady state, in addition to full-length perforin-2, two fragments have been robustly detected in murine DCs: a ~ 40 kDa MACPF domain (consistent with the predicted MW of 35.5 kDa plus two N-linked glycans), and a ~ 30 kDa P2 domain (theoretical MW 22–30 kDa, depending on the precise location of the shedding site, plus one N-linked glycan) (Fig. 1B) (Rodríguez-Silvestre et al, 2023; Ebrahimnezhaddarzi et al, 2022). Because the antibody against the C-terminal tail does not recognise the ~30 kDa P2 fragment, these observations suggest that two cleavage sites are present in perforin-2: one in the EGF domain, resulting in two fragments linked by a disulfide bond, and one responsible for ectodomain shedding (Fig. 1B).

To define the proteolytic steps involved in perforin-2 maturation, we first mapped candidate cleavage sites using previously published mass spectrometry data from whole cell lysates of MutuDC (Rodríguez-Silvestre et al, 2023) (Dataset EV1a). Figure 1C shows the positions of non-tryptic peptides—i.e., peptides likely generated in cells rather than produced by trypsin digestion of the lysates—identified in each of six independent samples. As a complementary approach, we used the Eukaryotic Linear Motif (ELM) database (Kumar et al, 2024) to identify known protease recognition motifs in the mouse perforin-2 sequence (indicated with dashed lines) (Fig. 1C; Dataset EV1B).

In addition to the previously reported promiscuous cleavage in the EGF loop (Rodríguez-Silvestre et al, 2023; Ebrahimnezhaddarzi et al, 2022), we observed five putative cleavage events: (1) a site at position L38^, which would remove a short N-terminal fragment; (2–3) two sites within the MACPF domain (D210^ and I296^); (4) one site in the P2 loop (N539^); and (5) a candidate shedding site in the CTT at M599^ (cleavage at position A19^ corresponds to signal peptide removal). Three of the five sites in the ectodomain (N-term, EGF and the P2 loop cleavages) are between cysteine residues linked by disulfides bond, and are therefore unlikely to result in major structural rearrangements in non-reducing conditions (Fig. 1C). We hypothesise that the two cleavages in the MACPF domain (D210^ and I296^) are involved in the disassembly of perforin-2, as they would compromise the integrity of the pore-forming domain. In line with this hypothesis, cleavage at D210^ corresponds to a putative caspase site and is therefore likely to occur following pore formation, when the β-barrel is accessible from the cytosol. The candidate shedding site is in the unstructured region of the CTT, 55 residues away from the transmembrane domain. In summary, the proteomics-based analysis uncovers a previously unappreciated level of proteolytic remodelling of perforin-2, which includes, but is not limited to, ectodomain shedding.

### P2 loop is cleaved by asparagine endopeptidase (AEP)

**Figure 2:**
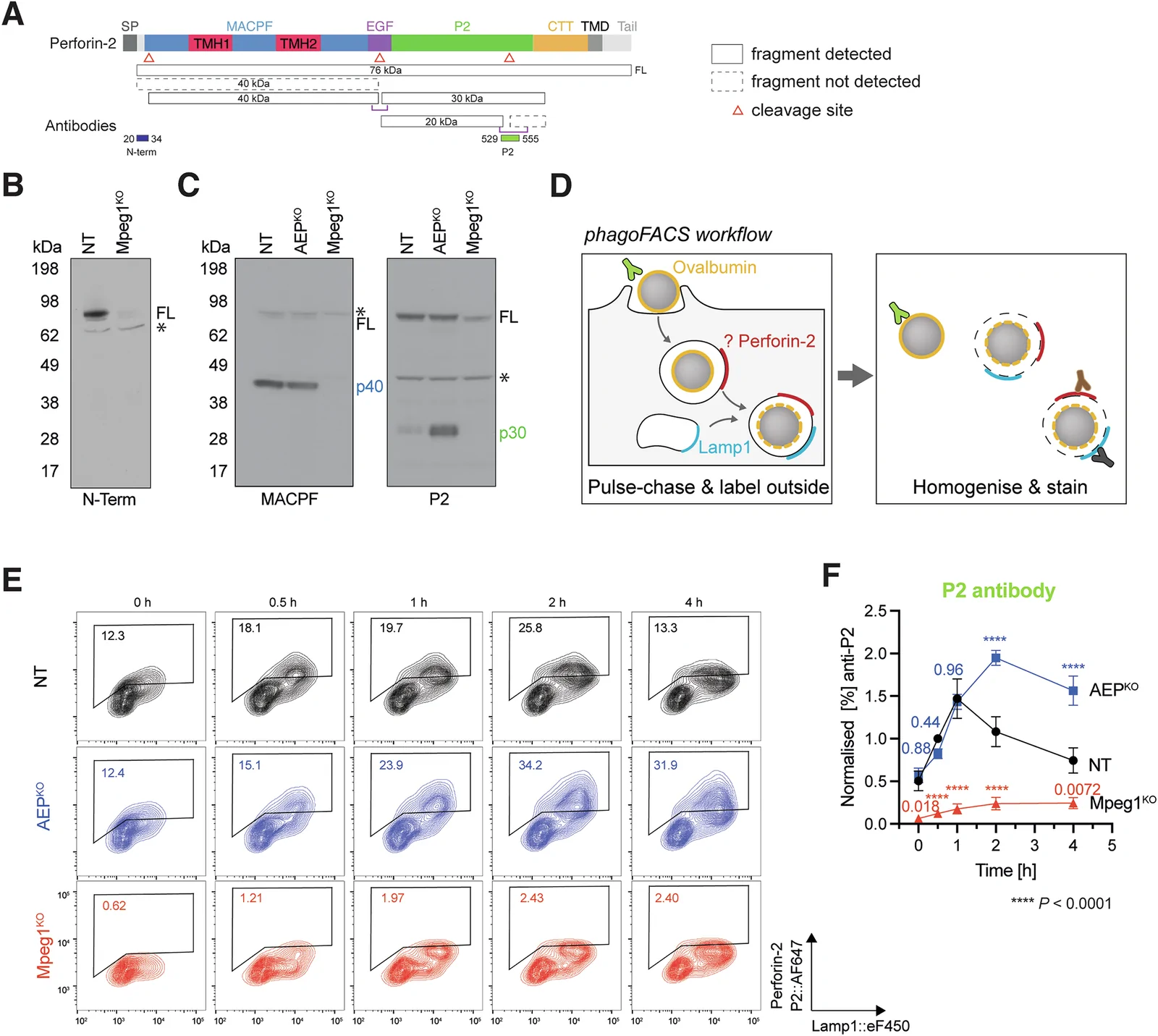
Perforin-2 is cleaved by AEP in the EGF and P2 domains. (**A**) Diagram illustrating the perforin-2 fragments relevant for Fig. 2 and the epitopes of antibodies raised in this study. (**B**) Immunoblot analysis of NT and Mpeg1^KO^ MutuDCs lysates performed under reducing conditions using the αN-term antibody. *Non-specific bands. The corresponding Ponceau S stain is shown in Appendix Fig. S1. (**C**) Immunoblot analysis of NT, AEP^KO^ and Mpeg1^KO^ MutuDCs lysates performed under reducing conditions using the αMACPF and αP2 antibodies. The corresponding Ponceau S stain is shown in Appendix Fig. S1. (**D**) Schematic of the phagoFACS assay: MutuDCs are pulsed with OVA-coated beads and chased for the indicated time. The cells are then shifted to 4°C to label beads that were not internalised, lysed, and stained with selected antibodies to analyse protein recruitment to phagosomes by flow cytometry. (**E**, **F**) PhagoFACS assay on NT, AEP^KO^, and Mpeg1^KO^ MutuDCs. Sample flow plots (**E**) and quantification of the percentage of αP2^+^ phagosomes (**F**). The data were normalised to the NT sample at 1 h. Mean and SEM. *P* values calculated using a two-way ANOVA with Dunnett’s multiple comparisons test versus NT MutuDCs. For all data, *n* = 3 independent experiments. Source data are available online for this figure.

To validate the cleavage sites identified by proteomics, we raised antibodies against the N-terminal end of perforin-2 (residues D21-C34) and the P2 loop (residues N539-C554) (Fig. 2A). The N-terminal antibody detected full-length perforin-2, but it did not recognise the 40 kDa MACPF fragment revealed by a commercial antibody with an unknown binding site (Fig. 2B,C; Appendix Fig. S1). Thus, these data confirm that the N-terminus is cleaved at an early stage of perforin-2 maturation, and suggest that this occurs prior to or concurrently with the cleavage in the EGF domain.

The antibody against the P2 loop detected full-length perforin-2 as well as a faint band corresponding to the P2 domain at 30 kDa (Fig. 2C). We have previously demonstrated that the EGF-linker is cleaved by AEP. In the absence of AEP, the EGF-linker is still cleaved, but the cleavage results in different tryptic fragments, suggesting that other proteases can compensate for AEP activity (Rodríguez-Silvestre et al, 2023). Data from AEP^KO^ cells also suggested that AEP might control an additional cleavage, located in the P2 loop, as the non-tryptic peptide p521-539 was depleted in the AEP^KO^ cells (Rodríguez-Silvestre et al, 2023). Indeed, in the absence of AEP, we observed accumulation of the 30 kDa P2 domain when probing with the N539-C554 P2 antibody (Fig. 2C). The cleavage in the P2 domain is corroborated by the previous observation of a faint 20 kDa fragment detected with a commercial αP2 antibody (Rodríguez-Silvestre et al, 2023). The immunoblotting data also suggest that cleavage in the P2 loop occurs after (or at the same time as) the EGF loop cleavage, as there is no accumulation of full-length perforin-2 (nor appearance of any other fragments) in the AEP^KO^ cells under reducing conditions.

**Figure EV1:**
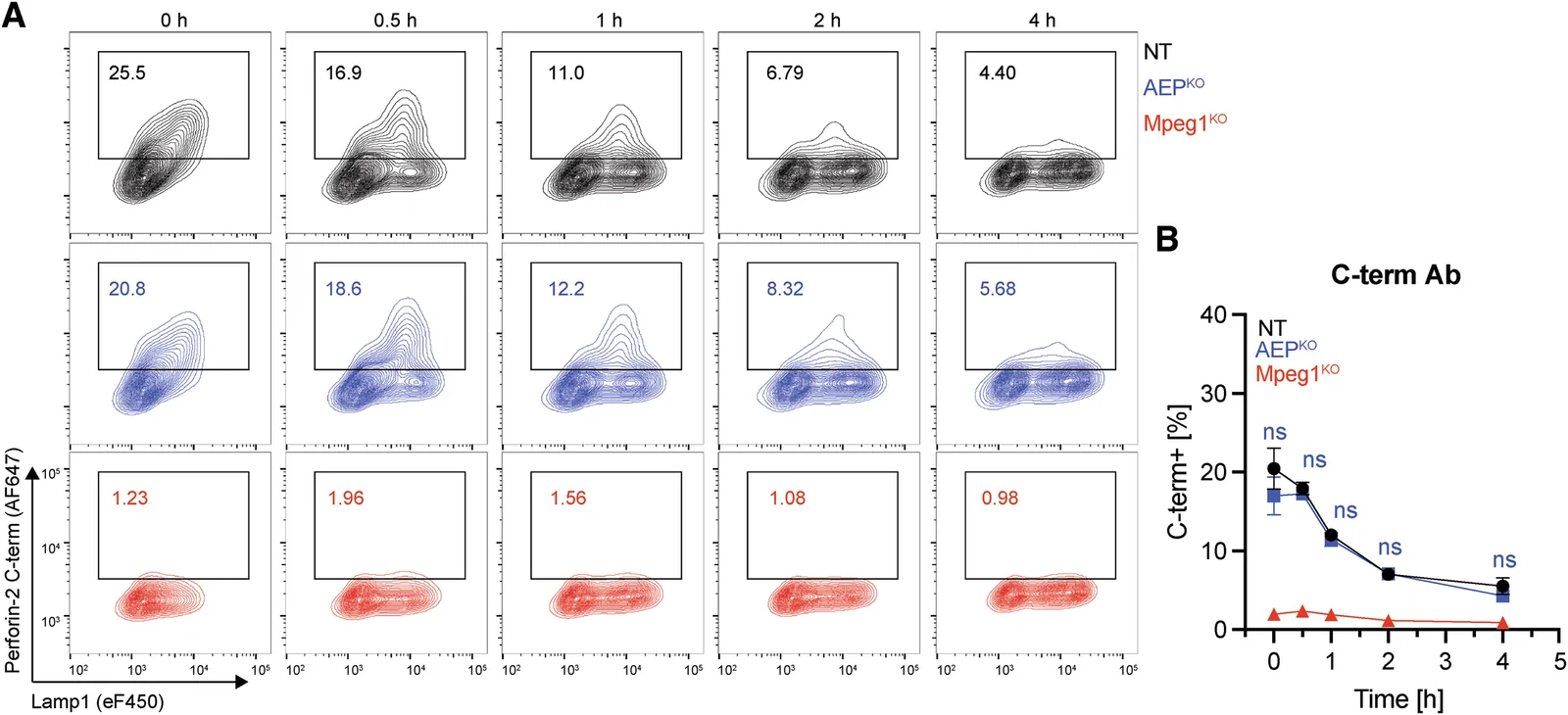
Perforin-2 recruitment to phagosomes in control, AEP^KO^, and Mpeg1^KO^ cells. (**A**, **B**) PhagoFACS assay as in Fig. 2E,F showing staining with αC-term (**A**) and % of αC-term^+^ phagosomes (**B**). Data represent mean and SEM of *n* = 3 independent experiments. ns, not significant using a two-way ANOVA with Dunnett’s multiple comparisons test versus NT MutuDCs.

To test whether the cleavage in the P2 loop occurs following recruitment to antigen-containing compartments, we analysed perforin-2 processing in phagosomes using the phagoFACS assay (Fig. 2D, Appendix Fig. S2) (Hoffmann et al, 2012). We pulsed the cells with ovalbumin (OVA)-coated beads, and at different time points, labelled the outside of the beads with anti-OVA antibody and lysed the cells. The phagosomes were then isolated and stained with epitope-specific antibodies against perforin-2 and Lamp1. As we reported previously, the signal from the C-term antibody, which detects full-length perforin-2, peaked within 30 min (Fig. EV1) and was then lost over time, most likely due to removal of the TMD from the membrane following ectodomain shedding. The signal from the P2-loop antibody peaked later, at 1 h (Fig. 2E,F), suggesting the P2 loop is cleaved at slower kinetics compared to shedding. In AEP^KO^ cells, initial recruitment of perforin-2 recognised by the P2-loop antibody follows similar kinetics to control cells, but at later time points, the signal continues to accumulate, consistent with the model where, in the absence of AEP, the P2 loop is no longer cleaved.

Together, these data demonstrate that N-terminal cleavage represents an early maturation event, whereas cleavage within the P2 domain occurs once perforin-2 is recruited to phagosomes and has undergone ectodomain shedding.

### TMD and the cytosolic tail are removed from the membrane by γ-secretase

**Figure 3:**
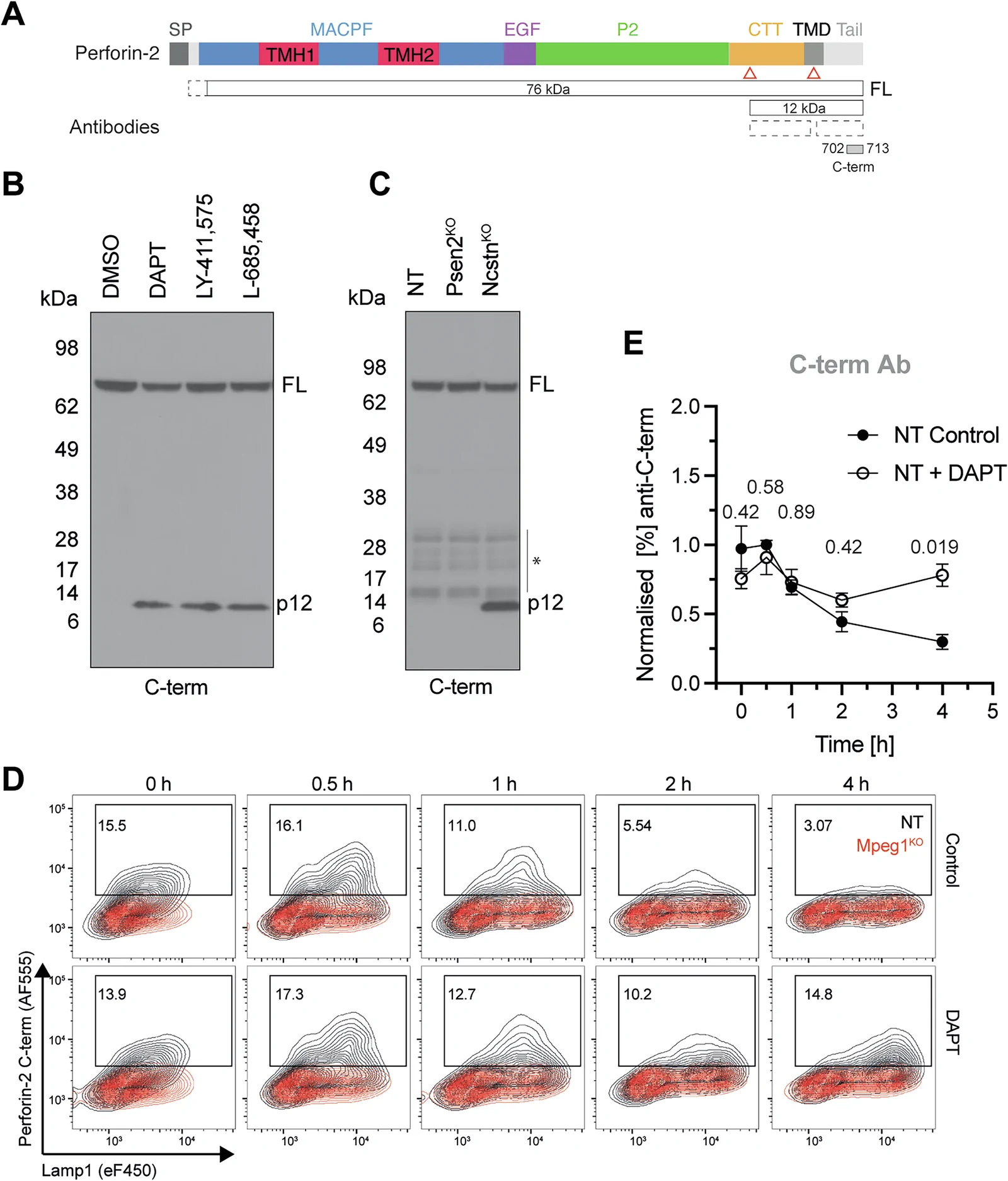
Perforin-2 is cleaved by γ-secretase. (**A**) Diagram illustrating perforin-2 fragments relevant for Fig. 3. (**B**, **C**) Immunoblot analysis of MutuDCs treated with DMSO (0.1% v/v), DAPT (10 μM), LY-411,575 (10 μM) or L-685,458 (10 μM) for 8 h and performed under non-reducing conditions using the αC-term antibody. *n* = 10 independent experiments for DAPT, and *n* = 2 for LY-411,575 and L-685,458. The corresponding Ponceau S stain is shown in Appendix Fig. S1. (**C**) Immunoblot of NT, Psen2^KO^, and Ncstn^KO^ MutuDCs lysates analysed under non-reducing conditions using the αC-term antibody. *n* = 2 independent experiments. The corresponding Ponceau S stain is shown in Appendix Fig. S1. (**D**, **E**) PhagoFACS assay (see Fig. 2D) on control and DAPT-treated (10 μM) MutuDCs performed with αC-term and αLamp-1 antibodies. Representative flow plots (**D**) and quantification (**E**). Data normalised to the control sample at 1 h. Data are mean and SEM. *n* = 3 independent experiments. *P* values calculated using a multiple unpaired *t* test (two-stage step-up, Benjamini, Krieger and Yekutieli) versus NT MutuDCs control. Source data are available online for this figure.

As we were unable to detect any fragments of cleaved perforin-2 with the C-terminal tail antibody, we predicted that following shedding, the TMD and the cytosolic tail are rapidly removed from the membranes. The best characterised intramembrane protease in the endocytic compartments is γ-secretase, which recognises substrates with short luminal domains (usually following a shedding step by other proteases) (Kopan and Ilagan, 2004; Funamoto et al, 2013). Indeed, when we treated the cells with γ-secretase inhibitors (DAPT, LY-411,575, and LY-685,458), we observed accumulation of a 12 kDa fragment detected with the C-term antibody (Fig. 3A,B). The size of the fragment is consistent with the predicted shedding site in the CTT domain, at position M599^. We also confirmed these results in MutuDC lines deficient in γ-secretase activity generated by knocking out the auxiliary subunit nicastrin (Ncstn) (Fig. 3C; Appendix Fig. S3). Knockout of the proteolytically active subunit, presenilin (Psen2), did not abolish the γ-secretase-mediated cleavage, most likely due to the homologous gene Psen1 (Fig. 3C; Appendix Fig. S3). In cells treated with γ-secretase inhibitor DAPT, we also observed accumulation of the C-terminal tail staining on phagosomes, in line with the hypothesis that the TMD and the cytosolic tail are removed from the membrane during phagosome maturation (Fig. 3D,E). These data are consistent with the model in which full-length perforin-2 is recruited to phagosomes, where the ectodomain is shed into the lumen and the TMD with the cytosolic tail are removed from the membrane by γ-secretase.

### Shedding is inhibited by the broad cathepsin inhibitor, Z-FA-FMK

**Figure 4:**
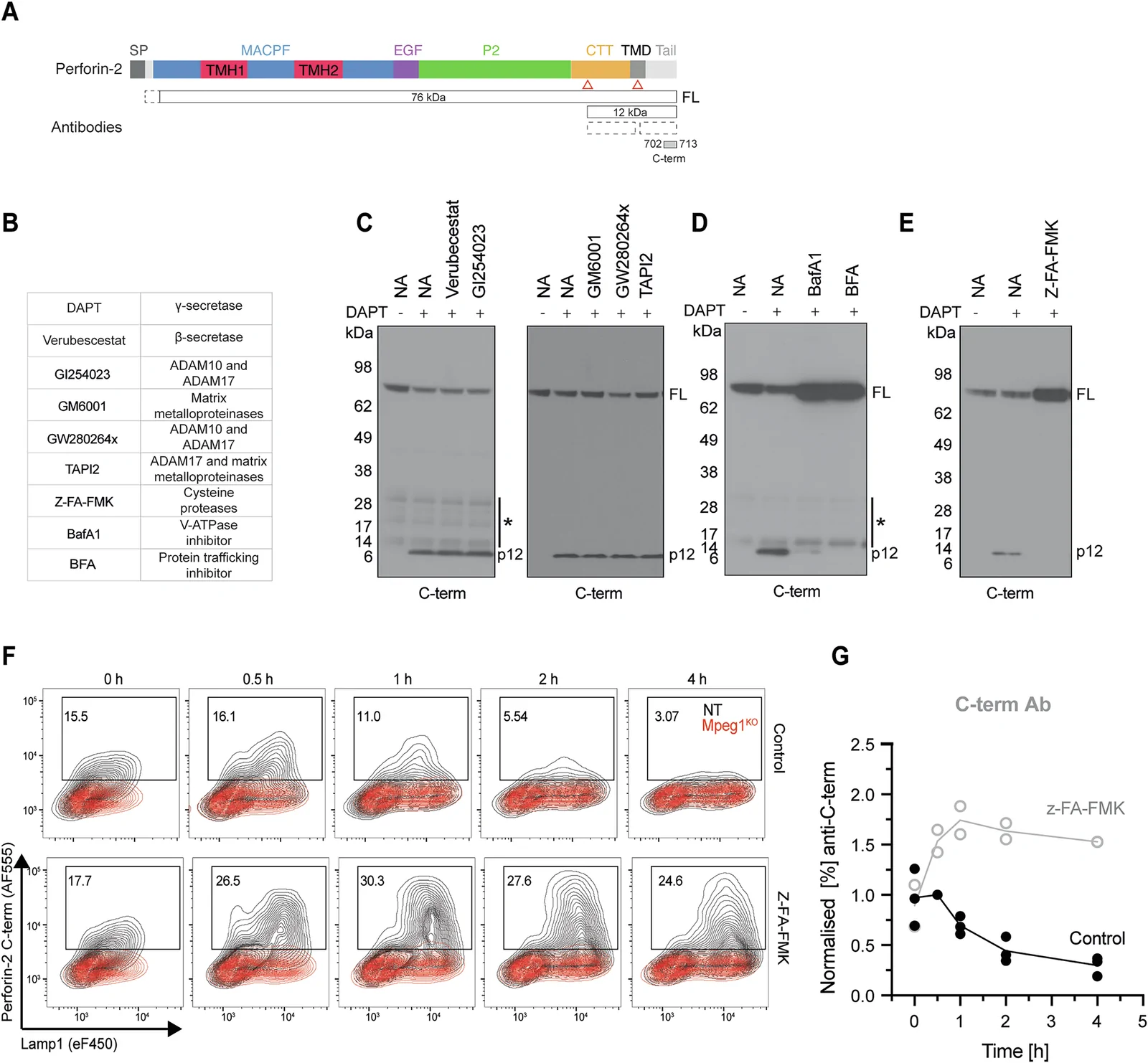
Ectodomain shedding is executed by cathepsins in acidified compartments. (**A**) Diagram illustrating perforin-2 fragments relevant for Fig. 3. (**B**) Small-molecule inhibitors and their targets. (**C**–**E**) Immunoblots of MutuDCs pre-treated with the indicated compounds for 2 h, followed by a 6-h treatment with DAPT (10 μM) in the continued presence of the respective inhibitors (**C**: 1 µM Verubecestat, 10 µM GI254023, 10 µM GM6001, 10 µM GW280264x, 20 µM TAPI2; **D**: bafilomycin A1 (0.5 μM), brefeldin A (5 μM); **E**: Z-FA-FMK (10 μM). Performed under non-reducing conditions using the αC-term antibody. The corresponding Ponceau S stains are shown in Appendix Fig. S1. *n* = 2 independent experiments. (**F**, **G**) PhagoFACS assay on NT and Mpeg1^KO^ MutuDCs performed in the presence or absence of Z-FA-FMK (10 μM), using αC-term and αLamp1 antibodies. Representative flow plots (**F**) and quantification (**G**). The flow cytometry plot for the control is reproduced from Fig. 3D as the Z-FA-FMK-treated sample shown was acquired in the same experiment. Data were normalised to control at 1 h. Individual data points from independent experiments are shown. Source data are available online for this figure.

In order to identify the mechanism of shedding, we screened small-molecule inhibitors for those that prevent the appearance of the 12 kDa perforin-2 fragment (TMD with the cytosolic tail) in the presence of DAPT (Fig. 4A). If the responsible sheddase were inhibited, the p12 fragment should no longer be generated and therefore would not accumulate upon γ-secretase inhibition. Most known sheddases belong to “a disintegrin and metalloprotease” (ADAM), beta-site APP cleaving enzyme (BACE) or matrix metalloprotease (MMP) families (Lichtenthaler et al, 2018). We therefore pre-treated MutuDCs for 2 h with a panel of inhibitors targeting these enzymes (Fig. 4B), followed by a 6-h incubation in the presence of DAPT. However, none of the classical sheddase inhibitors prevented the generation of the p12 fragment (Fig. 4C).

We then tested whether ectodomain shedding depends on perforin-2 delivery to phagosomes or phagosome acidification. We treated MutuDCs with an inhibitor of ARF GTPases, Brefeldin A, which blocks transport through the secretory pathway as well as with V-ATPase inhibitor, bafilomycin A1. Both treatments prevented the generation of the p12 fragment and resulted in the accumulation of full-length perforin-2 (Fig. 4D), suggesting that shedding might be mediated by lysosomal proteases. Indeed, we were able to inhibit shedding with Z-FA-FMK, which primarily targets cysteine proteases, including cathepsin B and L (Fig. 4E) (Hara et al, 1997; Citarella and Micale, 2020; Lopez-Hernandez et al, 2003). In line with these data, Z-FA-FMK resulted in strong accumulation of perforin-2 αC-term staining on phagosomes (Fig. 4F,G). Together, these results suggest that Z-FA-FMK targets the protease(s) responsible for perforin-2 ectodomain shedding and that shedding occurs once perforin-2-containing phagosomes acidify.

### Ectodomain shedding is not required for endocytic escape

**Figure 5:**
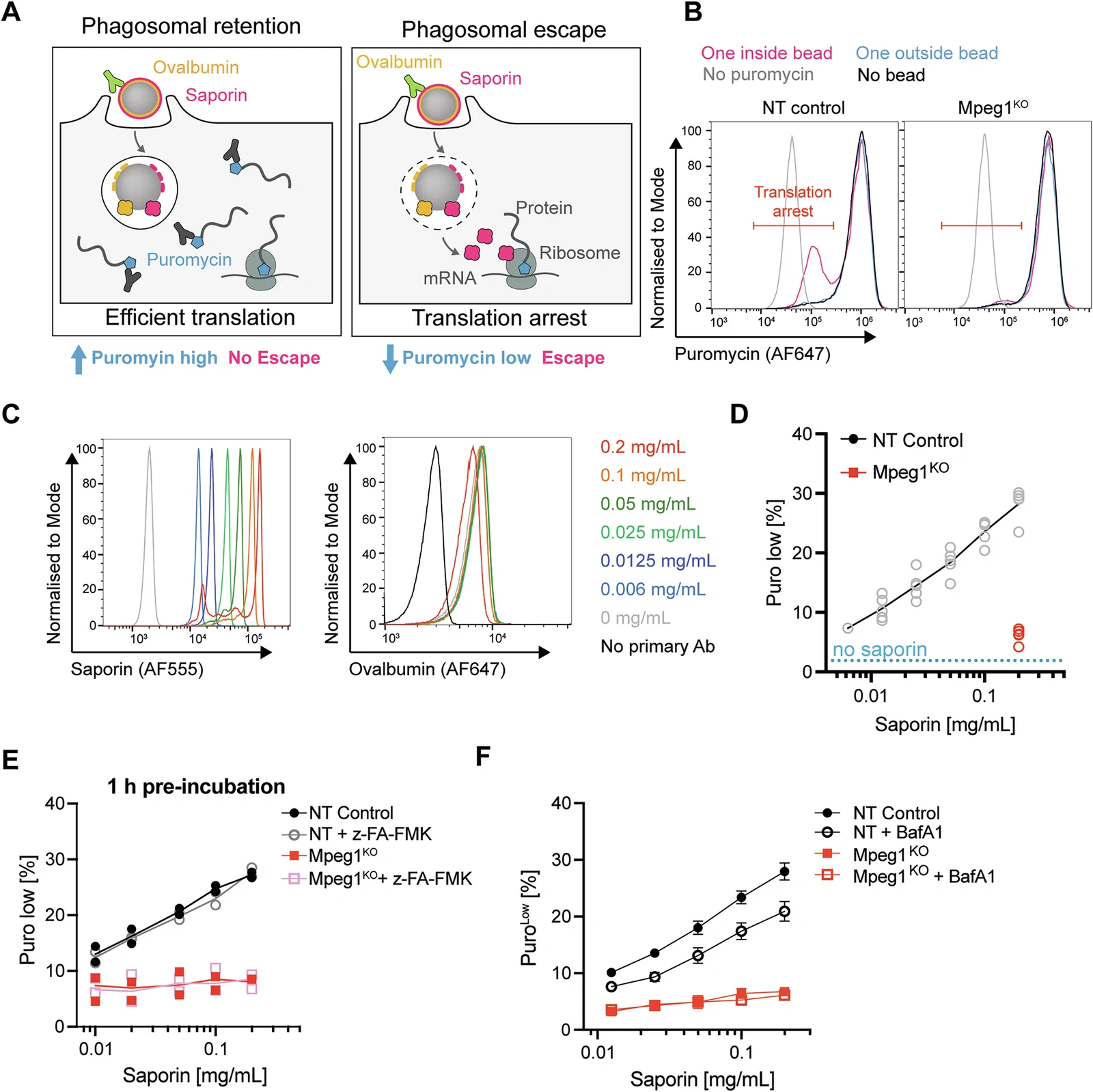
Proteolytic processing of perforin-2 is not required for endocytic escape. (**A**) Schematic of the saporin–puromycin phagosomal escape assay. The cells are incubated with ovalbumin- and saporin-coated beads for 5 h. When saporin escapes into the cytosol, it inactivates ribosomes, leading to translation arrest. To monitor translation efficiency, the cells are pulsed with puromycin for 30 min to label nascent polypeptides (thus, in cells where saporin escaped into the cytosol, puromycin incorporation is inefficient). Cells are then placed on ice and outside beads labelled with an αOvalbumin antibody. After fixation and permeabilisation, levels of puromycin incorporation are detected with an αPuromycin antibody. See also Appendix Fig. S4. (**B**) Saporin–puromycin assay on NT or Mpeg1^KO^ MutuDCs (see **A**). The population of cells where saporin escaped into the cytosol leading to translation arrest is highlighted. Individual data points from independent experiments are shown. (**C**) Staining of amino beads coated with ovalbumin followed by conjugation with the indicated concentrations of saporin. Beads were stained with αSaporin and αOvalbumin antibodies. (**D**) Saporin–puromycin assay (see A) of MutuDCs incubated with beads coated in different concentration of saporin. % of Puro^Low^ in Mpeg1^KO^ MutuDCs is shown for reference. Percentage of cells in translation arrest (PuroLow, see gate in **B**). *n* = 5 independent experiments. (**E**) Saporin–puromycin assay of NT and Mpeg1^KO^ MutuDCs pre-incubated for 1 h with Z-FA-FMK. Quantification of cells in translation arrest (Puro^Low^) based on the gating shown in (Appendix Fig. S3B). Individual data points from independent experiments are shown. (**F**) Saporin–puromycin assay on NT and Mpeg1^KO^ MutuDCs in the presence or absence of 0.5 µM bafilomycin A1. Quantification of cells in translation arrest (PuroLow) based on the gating shown in Appendix Fig. S4B). Data represent mean and SEM of three independent experiments with BafA1-treated Mpeg1^KO^ cells. Data represent mean and SEM of *n* = 4 independent experiments. Source data are available online for this figure.

To test whether proteolytic maturation of perforin-2 is required for pore formation in the phagocytic membranes, we employed a saporin-based endocytic escape assay (Fig. 5A; Appendix Fig. S4) (Rodríguez-Silvestre et al, 2023). Saporin is a ribosome-inactivating toxin (RIP), which, when delivered into the cytosol, leads to translation arrest (Nielsen and Boston, 2001). To monitor endocytic escape from phagosomes, we conjugated saporin to ovalbumin-coated beads using the heterobifunctional cross-linker SPDP, which introduces disulfide bonds. The bond can be reduced through the activity of phagosomal gamma-interferon–inducible lysosomal thiolreductase (GILT), resulting in the release of saporin into the phagosome lumen (Huotari and Helenius, 2011). To monitor saporin-mediated translation arrest, we labelled nascent polypeptide chains with puromycin (Schmidt et al, 2009). In line with previous data (Rodríguez-Silvestre et al, 2023), saporin escape from the beads was abolished in Mpeg1^KO^ MutuDCs (Fig. 5B). As reported before, in the saporin–puromycin assay, we observe two populations of cells: cells with efficient translation, and puromycin^low^ cells where saporin escaped into the cytosol, leading to translation arrest (Fig. 5B). This distribution of cells into two populations suggests that, once a small number of ribosomes are inactivated, there is a translational shutdown, possibly due to a ribotoxic stress response (Vind et al, 2020; Nielsen and Boston, 2001). To understand how the percentage of in cells in translation arrests correlates to the efficiency of saporin escape, we titrated the amount of saporin on the beads (Fig. 5C,D). In cells containing a single phagosome, a twofold dilution of saporin on the beads resulted in about 4% decrease in the percentage of cells in translation arrests, demonstrating that the assay is quantitative and that in Mpeg1^KO^ MutuDC antigen escape is at least 30-fold less efficient.

**Figure EV2:**
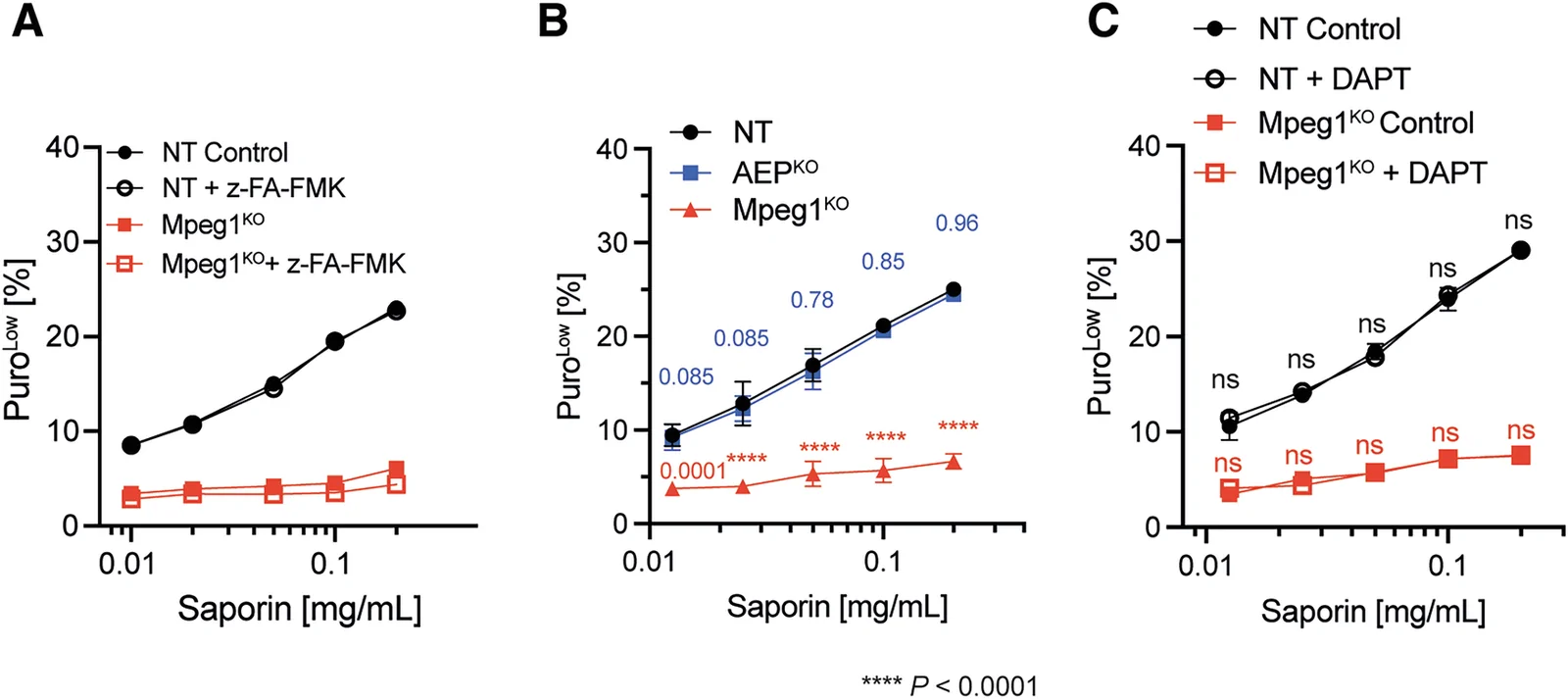
Efficiency of antigen escape in MutuDCs monitored by the saporin-puromycin assay. (**A**) Saporin–puromycin assay in NT or Mpeg1^KO^ MutuDCs pre-incubated for 16 h with Z-FA-FMK. (**B**) Saporin–puromycin assay in NT, AEP^KO^, and Mpeg1 ^KO^ MutuDCs. See also Appendix Fig. S4C). *n* = 3 independent experiments. *P* values calculated using a two-way ANOVA with Dunnett’s multiple comparisons test versus NT MutuDCs. (**C**) Saporin–puromycin assay in NT and Mpeg1^KO^ MutuDCs with and without DAPT. *n* = 3 independent experiments. ns, not significant using a multiple paired *t* test (two-stage step-up, Benjamini, Krieger and Yekutieli).

Unexpectedly, in cells treated with Z-FA-FMK to inhibit shedding, we saw no difference in the efficiency of saporin escape from phagosomes (Figs. 5E and EV2A). We were also not able to inhibit escape by interfering with the AEP-mediated cleavage in the P2 loop (Fig. EV2B), γ-secretase-mediated removal of the C-terminal fragment (Fig. EV2C), or with the promiscuous cleavage in the EGF domain (Appendix Fig. S5). Together, these data suggest that neither shedding nor the cleavages in the EGF and P2 domains are required for endocytic escape from phagosomes.

### Perforin-2-mediated endocytic escape is not abolished by the V-ATPase inhibitor

**Figure EV3:**
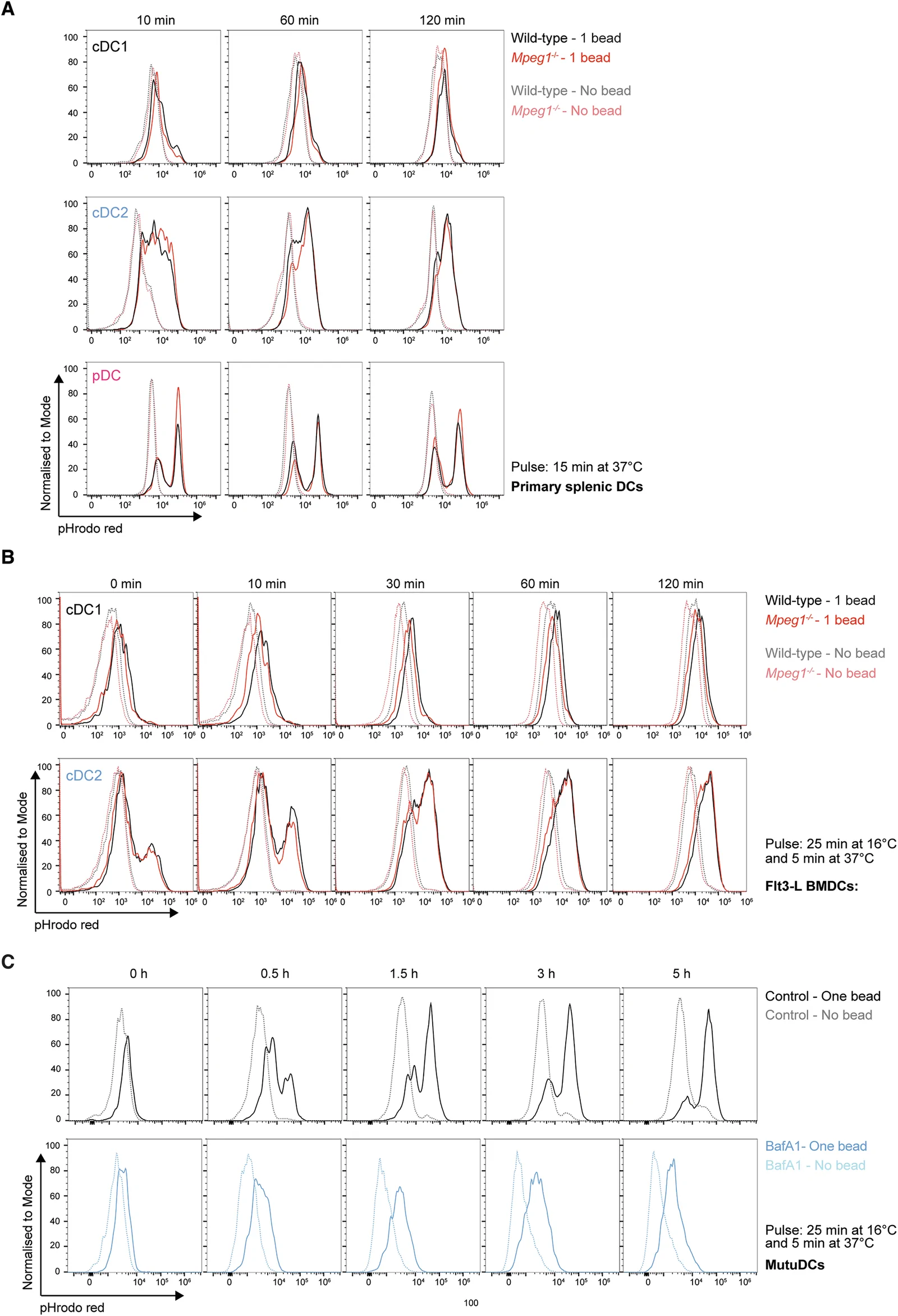
Analysis of phagosomal pH in dendritic cells. (**A**, **B**) pHrodo-bead assay on WT or Mpeg1^−/−^ splenic DCs were (**A**) or Flt3L-derived BMDCs (**B**). See also Appendix Fig. S6. Cells were pulsed with beads for 15 min at 37 °C and chased for the indicated time. Data representative of *n* = 2 independent experiments. (**C**) pHrodo-bead assay on MutuDCs in the presence or absence of 0.5 µM BafA1. Data representative of *n* = 5 independent experiments.

**Figure EV4:**
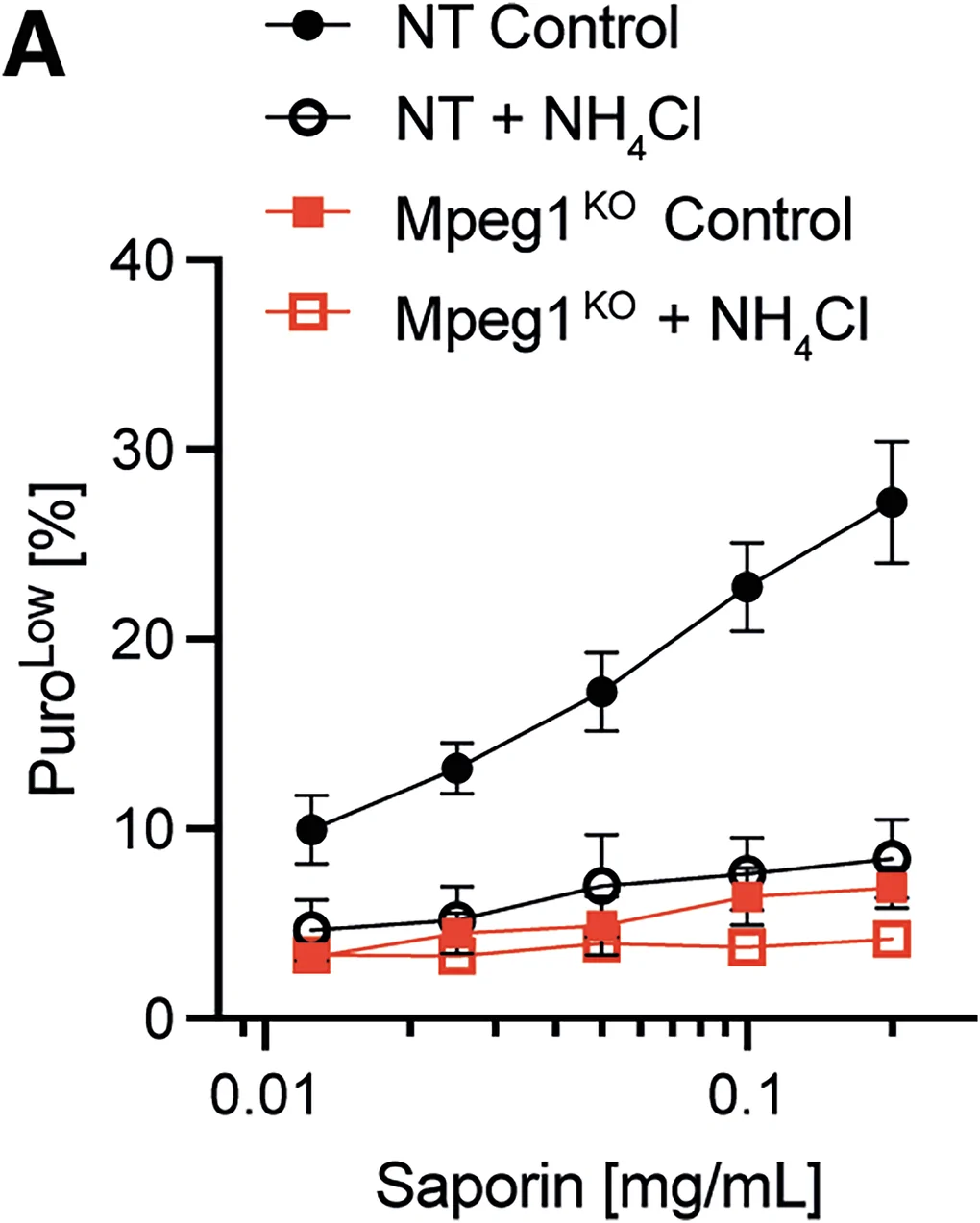
Efficiency of antigen escape in MutuDCs treated with NH_2_Cl to neutralise phagosomal pH. (**A**) Saporin–puromycin assay in NT and Mpeg1^KO^ MutuDCs with and without NH_4_Cl. *n* = 3 independent experiments.

Since shedding was inhibited in cells treated with BafA1 (see Fig. 4D), we went on to test whether phagosome acidification is required for perforin-2-mediated antigen delivery into the cytosol. Interestingly, in vitro, pre-pore to pore transition of perforin-2 has been achieved by incubating pre-pores at pH 4 (Ni et al, 2020; Pang et al, 2019; Jiao et al, 2021). However, cDC1s, which are the main cross-presenting cells (Hildner et al, 2008), and employ perforin-2 to deliver antigens into the cytosol (Rodríguez-Silvestre et al, 2023), were reported to maintain their phagosomes at near-neutral pH (Savina et al, 2009; Netting et al, 2025; Mantegazza et al, 2008). Therefore, the requirement of pH 4 for the formation of endocytic escape pores appeared inconsistent with the physiology of cDC1 phagosomes. Using pH-sensitive beads coated with pHrodo, we confirmed that phagosomal pH in both primary and Flt3L-derived cDC1s remains neutral, in contrast to cDC2 or pDCs (Fig. EV3A,B; Appendix Fig. S6). Importantly, the pHrodo experiments suggest that cDC1-like MutuDCs, in contrast to primary cDC1s, acidify their phagosomes (Fig. EV3C). Nevertheless, when treated with BafA1 to interfere with acidification (Fig. EV3C), MutuDCs were still able to import saporin from phagosomes into the cytosol (Fig. 5F, see also Appendix Fig. S4B). This was in contrast to treatment of the cells with a lysosomotropic weak base NH_4_Cl, which abolished escape (see Fig. EV4). Importantly, while treatment of MutuDCs with BafA1 significantly reduced phagosome acidification, a small increase in pHrodo fluorescence was still observed over time (Fig. EV3C). Thus, a pH gradient during phagosome maturation is likely relevant for perforin-2 activity. Nevertheless, saporin escape in BafA1-treated MutuDCs as well as in primary and Flt3L-derived cDC1s (Rodríguez-Silvestre et al, 2023), which maintain phagosomes at near-neutral pH, suggests that perforin-2-mediated endocytic escape does not require a strongly acidic environment. In contrast, shedding requires low pH, further supporting the model where full-length perforin-2 is employed during cross-presentation.

### Membrane anchoring by CTT is required for endocytic escape

If full-length perforin-2 mediates endocytic escape, the pores in phagosomes must be formed in the cis conformation, i.e., by rotating the MACPF domain towards the membrane on which the pre-pore sits (Yu et al, 2022; Jiao et al, 2021). According to this model, the individual MAPCF domains flip and propagate insertion in a clockwise fashion around the perforin-2 ring. Interestingly, during this propagation, the intermolecular interactions between the two neighbouring subunits are almost completely lost, as in addition to the MACPF flip, the P2 domain rotates along the axis roughly perpendicular to the membrane (Yu et al, 2022).

**Figure 6:**
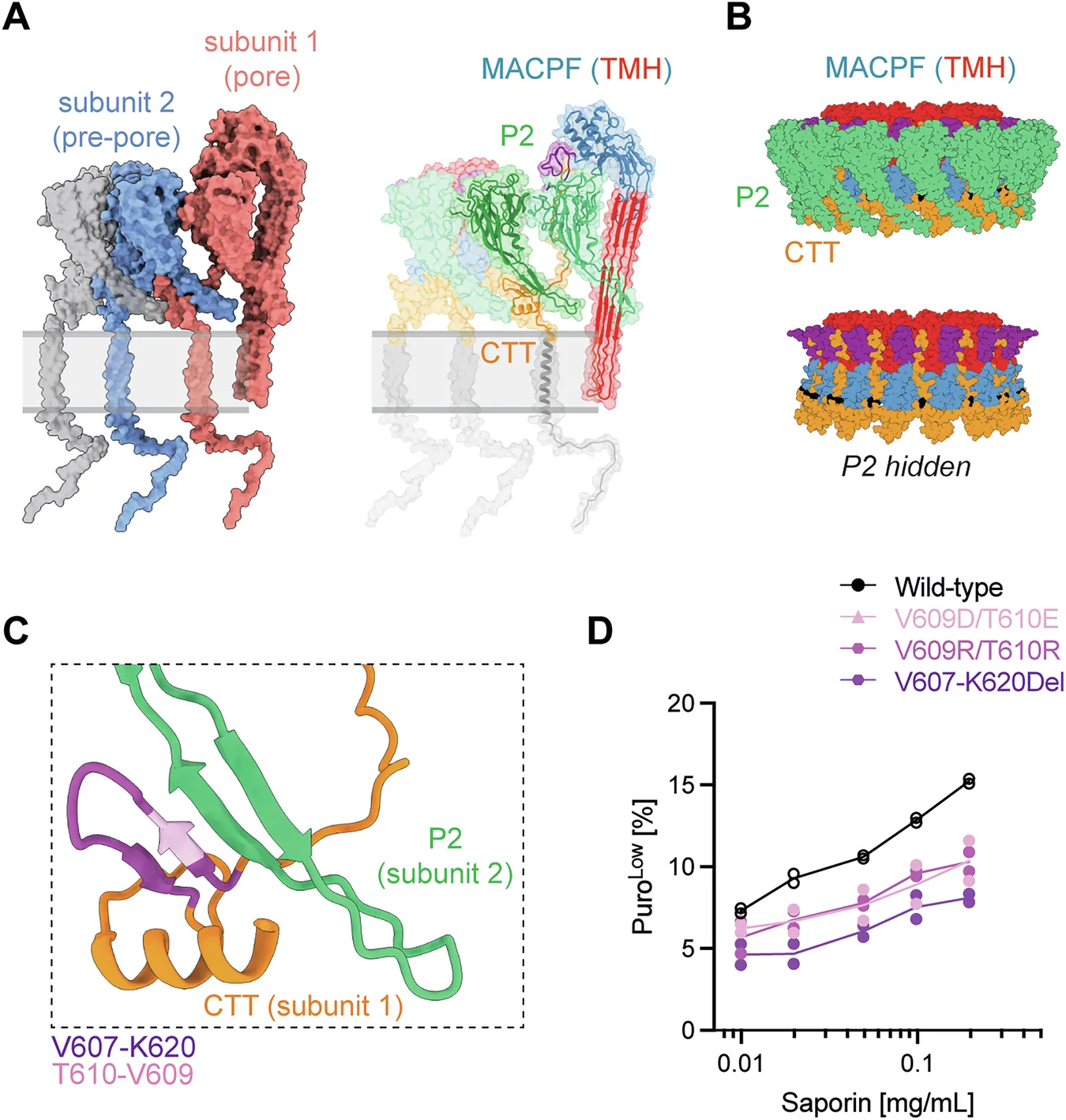
Anchoring via CTT domain is required for endocytic escape. (**A**) Predicted model of the pre-pore to cis pore conformational transition of full-length perforin-2 based on the experimental structures pdb:6sb3, pdb:6sb5 (Ni et al, 2020), and the AF-A1L314-F1 Alphafold model (Jumper et al, 2021). (**B**) Diagram showing CTT (including putative shedding site M599^S600 highlighted in black) concealed by the P2 domain in the pre-pore structure of perforin-2; pdb:6sb3 (Ni et al, 2020). (**C**) Diagram showing the interaction of CTT (orange) with neighbouring P2 domain (green); pdb:6sb3 (Ni et al, 2020). Mutated residues are shown in pink and purple. (**D**) Saporin–puromycin assay in Mpeg1^KO^ MutuDCs reconstituted with either WT Mpeg1 or with the indicated mutants. See also Appendix Fig. S7. Individual data points from independent experiments are shown. Source data are available online for this figure.

Importantly, however, in the available structures of the pre-pores (pdb: 6sb3) (Ni et al, 2020), the CTT domain wraps behind the P2 domain of the neighbouring subunit (Fig. 6A). This has two important implications. First, it suggests that the CTT-P2 interaction might in fact provide the majority of intermolecular interactions between the subunits, necessary to maintain the integrity of the ring during the MACPF flip (Fig. 6A). Second, it suggests that most of the CTT (including the putative M599^ shedding site) might not be accessible to proteases, because it is largely concealed by the P2 domain (Fig. 6B).

**Figure 7:**
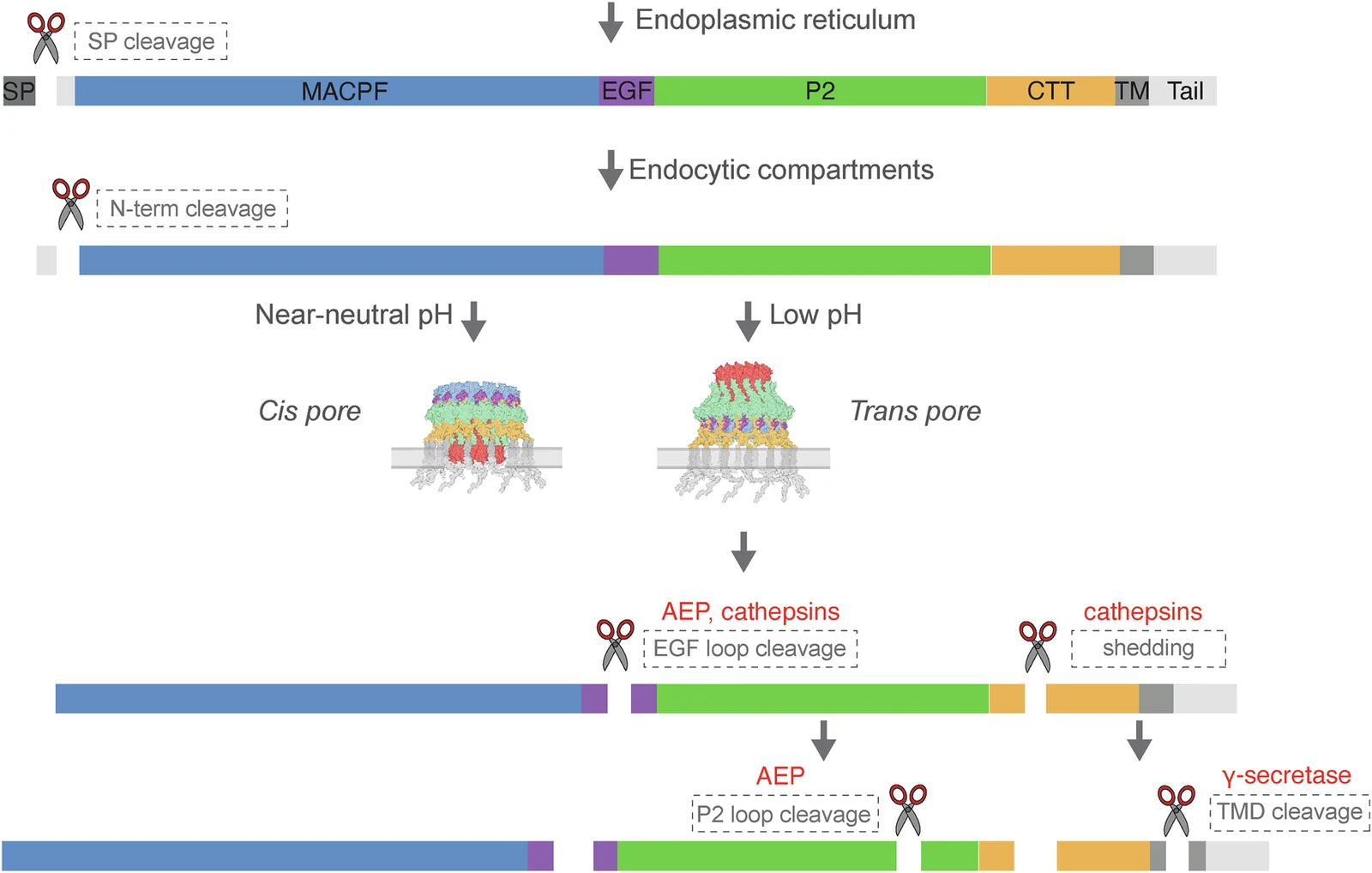
Schematic of the proposed steps involved in proteolytic maturation of perforin-2. The signal peptide is removed upon insertion into the ER membranes. A short N-terminal fragment is removed prior to any other cleavages (but it is unclear whether this happens after or before pore formation). In near-neutral, or mildly acidic organelles perforin-2 forms cis pores which mediate endocytic escape. In low pH compartments, the P2 domain flips to enable formation of trans pores in intravacuolar pathogens. Formation of the trans pores (or just the P2 flip) results in shedding and cleavage in the EGF domain. This is rapidly followed by an additional cleavage in the P2 loop and by the removal of the TMD and the cytosolic tail from the membrane by γ-secretase.

Thus, to establish whether the CTT is critical for endocytic escape, we introduced two charged residues, R609D/T610E or V609R/T610R, in the CTT β-hairpin that forms contacts with the neighbouring P2 (Fig. 6C). These point mutations significantly decreased the efficiency of the endocytic escape, and, surprisingly, the complete deletion of the β-hairpin, V607-K620Del, abolished escape (Fig. 6D; Appendix Fig. S7). Together, these data demonstrate the critical role of the CTT during the formation of endocytic escape pores (Fig. 7).

## Discussion

Perforin-2 is the most ancient, yet least characterised, member of the MACPF family. It has been implicated in two fundamentally distinct processes: the killing of intravacuolar pathogens during innate immune responses and the translocation of antigens across endocytic membranes during cross-presentation and T cell priming. How these two perforin-2 functions are regulated remains unclear. Here, we set out to identify the mechanism that permits controlled release of phagocytic cargo without compromising the integrity of intracellular compartments in dendritic cells. We demonstrate that anchoring of perforin-2 via the CTT and TMD to the phagosomal membrane is critical for escape.

The concept of regulating pore formation by proteolysis is well established for related pore-forming proteins. For example, perforin-1 requires the proteolytic removal of a C-terminal glycan that hinders oligomerisation (House et al, 2017; Uellner et al, 1997). The assembly of the complement membrane attack complex is regulated by a proteolytic cascade (Dunkelberger and Song, 2010). Similarly, members of the gasdermin family, which also form β-barrel pores, are activated through proteolytic activation by cytosolic caspases (Devant and Kagan, 2023). Our findings demonstrate that, similar to other pore-forming proteins, perforin-2 also undergoes extensive proteolytic remodelling with at least five cleavage sites characterised through mass spectrometry and immunoblotting. These include loss of the N-terminal helix, AEP-mediated cleavages in the EGF and P2 domains that result in three disulfide-linked fragments, as well as shedding of the ectodomain and γ-secretase-mediated release of the TMD from the membrane (see Fig. 7 for a schematic). Importantly, in cDC1-like cell line MutuDC, proteolytically cleaved perforin-2 appears to be the major species.

Unexpectedly, we report that perforin-2-mediated endocytic escape was not compromised when we interfered with its proteolytic maturation. Instead, our data point to a critical role of membrane anchoring by the CTT and TMD during formation of pores in the endocytic compartments, supporting the model where full-length perforin-2 mediates endocytic escape. The requirement of the TMD during pore formation is also consistent with the observation that the P2 domain does not bind mammalian lipids efficiently (Pang et al, 2019) and, therefore, it is unlikely that the ectodomain alone can insert into self-membranes. According to the model we propose, it is the proteolytic release of the ectodomain, rather than TMD-mediated anchoring, that serves to protect the endogenous membranes once phagosomes acidify. Importantly, since ectodomain shedding occurs in low pH compartments, it would explain how the dendritic cell cytosol is protected from hydrolytic enzymes present in acidified perforin-2-containing phagosomes or endolysosomes. We speculate that rapid acidification of phagosomes in other *Mpeg1-*expressing cell types, such as macrophages, might favour P2 domain flipping, revealing the shedding site and formation of proteolytically released trans pores in intravacuolar pathogens.

The mechanism of endocytic escape, where full-length perforin-2 forms cis pores in near-neutral pH compartments to facilitate antigen escape, explains several discrepancies in the proteolytic release model. (1) The proteolytically released ectodomain behaves as a soluble rather than a membrane-inserted protein in low pH compartments (Rodríguez-Silvestre et al, 2023). (2) The proton gradient across the phagosomal membrane is maintained during perforin-2-mediated endocytic escape (this work and Rodríguez-Silvestre et al, 2023). (3) cDC1s do not acidify their phagosomes (this work, Netting et al, 2025; Savina et al, 2006, 2009), yet they employ perforin-2 during cross-presentation. (4) A pre-pore lacking a TMD is unlikely to be able to associate with phagocytic membranes since the tip of the P2 domain shows preferential binding to bacterial rather than mammalian lipids (Ni et al, 2020). (5) The P2 domain is located in front of the CTT domain in the pre-pore conformation, protecting it from proteases and making it implausible that cleavage in the CTT could occur prior to cis pore formation (this work).

If ectodomain shedding by lysosomal enzymes is not the trigger for pore formation, what drives the formation of the cis pores? In vitro, perforin-2 pores were obtained by incubating pre-pores formed by perforin-2 ectodomain at pH 4 (Jiao et al, 2022). It is possible that these pores were formed in trans (by flipping of the P2 domain) rather than in cis (by flipping of the MACPF domain) and represent pores formed in acidic lysosomes to attack intravacuolar pathogens. Indeed, trans pores bridging two liposomes have been observed in cryo-electron microscopy data (Pang et al, 2019). Furthermore, it is still possible that in the early endocytic compartments, the mild drop of pH to ~6 is sufficient to drive the formation of the cis pores in the context of full-length perforin-2. Indeed, our data showed that while treatment with V-ATPase inhibitor BafA1 does not abolish endocytic escape, complete neutralisation of endocytic compartments with NH_4_Cl does. Alternatively, other environmental factors (such as lipids or calcium) or post-translational modifications (e.g. glycosylation or cleavage of the N-terminal fragment) could regulate pore formation in non-acidic compartments as has been described for other pore-forming proteins (Voskoboinik et al, 2005; Ding et al, 2016; House et al, 2017; Abrahamsen et al, 2024; Marini et al, 2023).

In summary, we demonstrate that full-length perforin-2 mediates antigen delivery into the cytosol of dendritic cells. We also propose that endocytic escape occurs via cis pores forming in endocytic compartments with near-neutral pH, and that pH drop favours formation and proteolytic release of trans pores, which attack pathogens. Thus, the TMD enables context-dependent functions of perforin-2 and repurposes the same effector for fundamentally different tasks in different cell types. This dual function might reflect the ancestral origin of perforin-2, from which complement and perforin-1 later diverged as specialised effectors for microbial killing and delivery of effectors across mammalian membranes.

## Methods

**Table:** Reagents and Tools Table

Reagent/resource	Reference or source	Identifier or catalogue number
**Experimental models**		
MutuDCs 1940	ABM; Marraco et al, 2012	T0528
C57BL/6 mice	Jackson Lab	
Mpeg1^−/−^ mice	Rodriguez-Silvestre et al, 2023	
**Recombinant DNA**		
Lenti-sgRNA-hygro	Addgene	#104991
Lenti-sgRNA-hygo-Ncstn	This work	
Lenti-sgRNA-hygo-Psen2	This work	
psPAX2	Addgene	#12260
pMD2.g	Addgene	#12250
pHR-mScarlet	Derived from Addgene #60906	#60906
pHR-MPEG1-IRES-mScarlet	Derived from Addgene #60906	#60906
**Antibodies**		
Biotin Streptavidin eF450	Thermo	48-4317-82
Biotin Streptavidin AF488	Thermo	S11223
Rabbit anti-BiP	Cell Signaling	3177 T
Rabbit anti-Cathepsin B	Cell Signaling	31718 T
CD11b M1/70 Rat eF450	Thermo	48-0112-82
Hamster anti-CD11c BV605	BioLegend	117333
Hamster anti-CD11c FITC	BioLegend	117306
Rat anti-CD172a BV421	BD	740071
Rabbit anti-Cytochrome C	Cell Signaling	11940 T
Rat anti-F4/80 BUV395	BD	565614
Goat IgG Polyclonal Donkey AF488	Thermo	A11055
Rat anti-Lamp1 Biotin	Thermo	13-1071-92
Rat anti-Lamp1	BioLegend	121601
Rat anti-MHC-II BV785	BioLegend	107645
Mouse IgG Polyclonal Donkey AF647	Thermo	A31571
Mouse IgG Polyclonal Donkey AF555	Thermo	A31570
Rabbit anti-Nicastrin	BioLegend	852301
Ovalbumin Polyclonal Goat	OriGene	AP33373SU-N
Ovalbumin Tosgaa1 Mouse	BioLegend	520402
Ovalbumin Polyclonal Rabbit	Merck	C6534
Rabbit anti- Perforin-2 MACPF Polyclonal	Cloud-clone	PAB352Mu01
Rabbit anti- Perforin-2 N-term Polyclonal raised against the epitope DKPLGETGTTGFQIC (D21 - C34)	Cambridge Research Biochemicals	Custom made
Rabbit anti- Perforin-2 P2 Polyclonal raised against the epitope NSDTAKDVRAPSLKKC (N539-C554)	Cambridge Research Biochemicals	Custom made
Rabbit anti- Perforin-2 C-term Polyclonal raised against the epitope TVLNGEEDPSPA (T702-A713)	Cambridge Research Biochemicals; Rodriguez-Silvestre et al, 2023	Custom made
Rabbit anti- Presenilin 2	Cell Signaling	9979T
Mouse anti- Puromycin Mouse AF647	Merck	MABE343-AF647
Mouse anti- Puromycin Mouse	Merck	MABE343
Rabbit IgG Polyclonal Donkey AF555	Abcam	Ab150062
Rabbit IgG Polyclonal Donkey AF647	Abcam	Ab150075
Rabbit IgG Polyclonal Donkey AF680	Thermo	A10043
Rabbit IgG Polyclonal Donkey AF488	Abcam	Ab150073
Rabbit anti- Saporin Polyclonal	Thermo	PA1-18425
Rat anti- Siglec-H APC	Thermo	17-0333-82
Mouse anti- Vps35	Santa Cruz	Sc-374372
Mouse anti- Xcr1 BV785	BioLegend	148225
**Oligonucleotides and other sequence-based reagents**		
Psen2-sgRNA for KO generation	IDT	Table EV1
Ncstn-sgRNA for KO generation	IDT	Table EV1
Psen2 primer for KO validation	IDT	Table EV2
Ncstn primer for KO validation	IDT	Table EV2
GeneBlock for V609R/T610R mutant	IDT	Table EV3
GeneBlock for V609D/T610E mutant	IDT	Table EV3
GeneBlock for V607-K620del mutant	IDT	Table EV3
**Chemicals, enzymes and other reagents**		
Fixable Viability Dye ViaKrome808	Beckman	C36628
DAPT: γ-secretase	Cambridge Bioscience	SM15-10
E64D: Cysteine proteases	Merck	E3132
GI254023: ADAM10 and ADAM17	Bio-Techne	3995
GM6001: Matrix metalloproteinases	Bio-Techne	2983
GW280264x: ADAM10 and ADAM17	Bio-Techne	7030
Leupeptin A: Serine/Cysteine proteases	Merck	L2884
LY-411,575: γ-secretase	Miltenyi	130-103-924
LY-685,458: γ-secretase	Bio-Techne	2627
Pepstatin A: Aspartic proteases	Merck	P5318
TAPI-2: ADAM17 and matrix metalloproteinases	Bio-Techne	6013
Verubecestat: β-secretase	Cambridge Bioscience	21115
Z-FA-FMK: Cysteine proteases	Santa Cruz	Sc-201303
Z-VAD-FMK: Cysteine proteases	Stratech Scientific	A1902-APE
20X TBS buffer	Thermo	28358
Amicon Ultra-15 centrifugal filters	Amicon	UFC9003
Bafilomycin A1	Cayman	11038
BCA protein assay kit	Thermo	23227
BD Cytofix/Cytoperm kit	BD Biosciences	554714
BlpI	NEB	R0585S
Bolt Reducing agent	Thermo	NP0009
Brefeldin A	Santa Cruz	Sc-200861c
BSA	Merck	A7979
BsmBI	NEB	R0739
BstXI	NEB	R0113L
Bsu36I	NEB	R0524S
CO_2_-independent medium	Thermo	18045088
Dialysed FBS	Thermo	A3382001
DMEM	Gibco	31966021
DNAse I	Merck	11284932001
DNeasy blood and tissue kit	Qiagen	69504
DTT	Merck	D9779
EasyTag™ EXPRESS35S Protein Labeling Mix, [35S]-, 2 mCi	Perkin Elmer	NEG772002MC
ECL prime reagent	Cytiva	RPN2236
ECL reagent	Cytiva	RPN2209
EDTA	Thermo	15575020
EDTA-free protease inhibitor cocktail	Roche	11873580001
Endo H	NEB	P0702L
EZ-Link Sulfo-NHS-LC-Biotinylation Kit	Thermo	21435
FBS	HyClone	SH30084.03
Flat3 Ligand	Miltenyi	130-097-372
Gel extraction kit	Qiagen	28706×4
Glutamax	Gibco	12440053
Glutaraldehyde	Merck	G5882
Glycine	Merck	G5417
GM-CSF	Miltenyi	130-095-746
HEPES	Gibco	15630106
Hygromycin B	Thermo	10687010
IMDM	Gibco	31980030
Imidazole	Merck	56748
Liberase-TL	Merck	5401020001
Lysotracker Red DND-99	Thermo	L7528
MluI-HF	NEB	R0198S
Mouse pan-dendritic cell isolation kit	Miltenyi	130-1000-875
NaCl	Fisher Scientific	S/3120/63
Non-essential amino acids	Gibco	11140050
NuPage LDS sample buffer	Thermo	NP0007
Ovalbumin	Worthington	LS0030540
PBS	Thermo	14190144
PCR purification kit	Qiagen	28104
Penicillin/streptomycin	Gibco	11548876
pHrodo iFL Red STP ester, amine reactive dye	Thermo	P36011
Plasmid maxi kit	Qiagen	12162
Plasmid mini kit	Qiagen	12123
PMSF	Merck	P7626
PNGase F	NEB	P0704L
Polybead Amino Microspheres 3.00 μm	Polysciences	17145-5
Puromycin	Thermo	A1113803
Q5 DNA polymerase	NEB	M0491S
Quick CIP	NEB	M0525S
Red Blood Cell Lysis Buffer Hybri-Max	Merck	R7757-100ML
RIPA buffer	Thermo	R0278
RPMI-1640	Gibco	6180036
RPMI-1640 lacking methionine and cystine	Merck	R7513
Saporin	Merck	S9896
SbfI-HF	NEB	R3642L
SDPD	Thermo	21857
SnaBI	NEB	R0130S
Sodium pyruvate	Gibco	11360070
Sucrose	Fisher Scientific	1046150
T4 DNA ligase	NEB	M0202
T4 Polynucleotide Kinase	NEB	M0201S
Top10 chemically competent E. coli	Thermo	C404003
TransIT-LT1	Mirus	MIR2304
Traut’s reagent	Thermo	26101
Triton X-100	Fisher Bioreagents	BP151-500
Trypsin	Merck	T6567
Tween-20	G-Bioscience	786-517
Zeba Spin 7 K MWCO desalting columns	Thermo	10415545
β-mercaptoethanol	Gibco	31350010
μ-slide 8-well chamber	Ibidi	80826
InstantBlue Coomassie stain	Abcam	Ab119211
**Software**		
GraphPad Prism		10.6.0 for Mac
FlowJo		10.8.2 for Mac
R studio		2024.09.1 + 394 for Mac
R		4.4.2 for Mac
Tidyverse		2.0.0 (Mac)
**Other**		

### Animals

All mice were bred/maintained in pathogen-free conditions by the Medical Research Council ARES facility. Experiments were approved by the LMB Animal Welfare and Ethical Review Body, and the UK Home Office (under the project license PP3605301). C57BL/6 and Mpeg1^−/−^ (Rodríguez-Silvestre et al, 2023) mice used were 8–13 weeks old when culled for tissue collection.

### Antibodies and reagents

All antibodies and reagents used in this study are listed in the Reagents and Tools Table.

### Cell lines and cell culture

MutuDCs from Abm, as well as those received as a kind gift from Hans Acha-Orbea, were used throughout this study. The cells were maintained in IMDM supplemented with 8% heat-inactivated FBS, 10 mM HEPES, 500 μM 2-mercaptoethanol, 1× GlutaMAX, penicillin (100 units/mL) and streptomycin (100 μg/mL) at 37 °C and 5% CO_2_. HEK-293Ts were maintained in DMEM supplemented with 10% heat-inactivated FBS and 1× GlutaMAX at 37 °C and 5% CO_2_. Cell lines are routinely tested for mycoplasma.

### Generation of Flt3L-BMDCs

Bone marrow was recovered from femurs and tibiae of from 8 to 13-week-old male or female C57BL/6 J mice and resuspended in RPMI-1640 supplemented 10% heat-inactivated FBS, 10 mM HEPES, 500 μM 2-mercaptoethanol, 1× sodium pyruvate, 1× non-essential amino acids, penicillin (100 units/mL) and streptomycin (100 μg/mL). For Flt3-L cultures, 15 × 10^6^ cells were seeded in a non-treated 10 cm dish and supplemented with 5 ng/mL GM-CSF and 200 ng/mL Flt3-L (Day 0). Cells were differentiated for 10 days with the addition of 10 mL of fresh media with 5 ng/mL GM-CSF and 200 ng/mL Flt3-L on Day 5 and 5 mL of fresh media containing 20 ng/mL GM-CSF on Day 7.

### Isolation of splenic DCs

Spleens were perfused with a solution of RPMI-1640, 0.1 mg/mL Liberase-TL and 0.1 mg/mL DNAse I, minced and digested for 25 min at 37 °C. Heat-inactivated FBS was added at 10% (v/v) to stop the digestion, before mashing tissues through a 70-μm filter. Red blood cells were lysed with Hybri-Max for 3 min at room temperature. Dendritic cells were isolated using a Miltenyi Pan-DC Isolation Kit (negative selection) according to the manufacturer’s instructions.

### Generation of Mpeg1 mutants

pHR-mMpeg1-IRES-mScarlet-V609D/T610E, pHR-mMpeg1-IRES-mScarlet-V609R/T610R and pHR-mMPEG1-IRES-mScarlet-V607-K620del Mpeg1 variants were generated by cloning custom GeneBlock DNA fragments (IDT, see Reagents and Tools Table for sequences) into pHR-MPEG1-IRES-mScarlet vector (Rodríguez-Silvestre et al, 2023). Vectors and gene fragments were digested using *Sbf*I and *Blp*I restriction enzymes for 2 h at 37 °C. Digested vectors were gel-purified from a 1% agarose gel using the QIAquick Gel Extraction Kit, and digested gene fragments were purified using the QIAquick PCR Purification Kit. Ligations were performed using T4 DNA ligase according to the manufacturer’s instructions. Transformations were performed using Top10 chemically competent *E. coli* according to the manufacturer’s instructions.

### Generation of sgRNA expression plasmids

sgRNAs were designed using the VBC score tool (https://www.vbc-score.org/) by choosing the highest-ranked sgRNA for each gene (Michlits et al, 2020). The chosen sgRNAs target exon 8 and exon 6 of Ncstn and Psen2, respectively. To generate sgRNA expression plasmids, lenti-sgRNA-hygro (t) was digested with BsmBI, dephosphorylated using Quick CIP according to the manufacturer’s instructions and purified using the QIAquick PCR purification kit. The sgRNA oligos were purchased as IDT RxnReady Primer Pools (Reagents and Tools Table). The oligos were phosphorylated using T4 Polynucleotide Kinase according to the manufacturer’s instructions, heated to 95 °C for 5 min to ensure hybridisation, and inserted into lenti-sgRNA-hygro plasmid.

### Lentiviral transduction of MutuDCs

For lentiviral production, 3 × 10^6^ HEK-293T cells were seeded into a 10-cm dish. The next day, the cells were transfected with a mixture of 45 μL TransIT-LTI, 7 μg of the expression vector with the gene of interest along with 7 μg psPAX2 and 0.7 μg pMD2.g in 1 mL of OptiMEM. The medium was replaced 18 h post transfection with fresh medium supplemented with 1% BSA. After 48 h post transfection, viral supernatant was collected, passed through a 0.45-μm-syringe filter and 10× concentrated using Amicon Ultracentrifugal filters with a 100 kDa MW cut-off. Aliquots were stored at −80 °C. For transduction, 2.5 × 10^6^ MutuDCs were seeded in a 6-cm dish in 3 mL of media. After letting the cells adhere for at least 2 h, 1 mL of 10× concentrated virus was added in the presence of 6 μg/mL polybrene. The next day, the virus was removed and replaced with fresh culture medium. Two days post-transduction, cells were selected with 200 μg/mL hygromycin for 1 week. *Mpeg1* mutants were transduced into Mpeg1^KO^ MutuDCs, and sgRNA guides for creating γ-secretase-deficient cells were transduced into Cas9-expressing MutuDCs (Rodríguez-Silvestre et al, 2023). To test the knockout efficiency, genomic DNA was isolated, and regions around the cut sites were amplified by PCR (Reagents and Tools Table). The amplicons were sequenced and analysed using TIDE (http://shinyapps.datacurators.nl/tide/).

### Preparation of ovalbumin-coated beads (OVA beads) for the phagoFACS assay

Amino-modified microspheres (Reagents and Tools Table) with a diameter of 3 μm were washed twice in PBS and preactivated with 8% (vol/vol) glutaraldehyde for 4 h at room temperature. Preactivated beads were washed once in PBS and then incubated overnight at 4 °C with ovalbumin (Reagents and Tools Table) at a concentration of 0.5 mg/mL in PBS. The next day, beads were quenched in 0.4 M glycine in PBS for 30 min, washed twice in PBS and used immediately for phagocytosis.

### Preparation of saporin-coated beads for the phagocytic escape assay

Saporin and BSA (Reagents and Tools Table) were conjugated to OVA beads through disulfide bonds. For efficient conjugation, free sulfhydryl groups were introduced by reacting 0.6 mL of 2.5 mg/mL saporin or BSA with 12 μL of 2 mg/mL Traut’s reagent in PBS containing 2 mM EDTA for 60 min at room temperature. Excess reagent was removed using a Zeba spin desalting column (Reagents and Tools Table) equilibrated with PBS containing 2 mM EDTA. OVA beads were resuspended in PBS containing 2 mM EDTA and reacted with 1 mM SPDP for 30 min at room temperature. The beads were then washed twice in PBS containing 2 mM EDTA and incubated with different mixtures of cysteine-modified saporin and BSA overnight at room temperature. Saporin was titrated (1 mg/mL to 0 mg/mL) and substituted with BSA so that the total protein concentration remained constant at 1 mg/mL. The next day, beads were washed three times in PBS and immediately used for phagocytosis.

### Preparation of pHrodo-coated beads

Amino-modified microspheres with a diameter of 3 μm were washed twice in PBS, resuspended in 100 mM sodium bicarbonate (pH 8.5) and reacted with 0.2 mM pHrodo iFL Red Ester dye (Reagents and Tools Table) for 1 h at room temperature. The beads were then washed once in PBS, and any reactive ester moieties were quenched by incubating the beads in 1× TBS for 10 min. After an additional two washes in PBS, the beads were coated with 1 mg/mL ovalbumin (Reagents and Tools Table) by passive absorption for 1 h at room temperature. The beads were then washed three times in PBS and immediately used for phagocytosis.

### PhagoFACS assay

MutuDCs were collected, washed once in PBS and resuspended in ice-cold internalisation medium (CO_2_-independent medium containing 1× GlutaMAX) to a density of 20 × 10^6^ cells/mL. OVA beads were added at a 10:1 ratio of beads: cells and incubated for 25 min at 16 °C followed by a 5-min incubation at 37 °C to allow phagocytic binding and internalisation of beads. To remove non-internalised beads, cells were first washed twice with 10 mL ice-cold PBS at 100×*g* for 4 min at 4 °C and then resuspended in 1 mL PBS, applied to a 5 mL FBS cushion and centrifuged at 150×*g* for 4 min at 4 °C. The cell pellet was then resuspended to 20 × 10^6^ cells/mL in cell culture medium and divided into different time points comprising 5 × 10^6^ cells each. For drug treatments, the culture medium was supplemented with 0.5 μM bafilomycin A1, 0.1 μM brefeldin A, 10 μM DAPT or 10 μM Z-FA-FMK. The chase was performed at 37 °C for different periods of time and stopped by adding ice-cold PBS. Non-internalised beads were stained on ice with a goat αOvalbumin antibody (1:100) (see Reagents and Tools Table for all antibodies) for 30 min followed by an αGoat-AF488 antibody (1:200) for 30 min. Cells from each time point were resuspended in 0.5 mL homogenisation buffer (250 mM sucrose, 3 mM imidazole, 2 mM DTT, 2 mM PMSF and 1× protease inhibitor cocktail, pH 7.4) and passed 25 times through a 22-G needle. Intact cells and debris were pelleted by centrifugation at 150× *g* for 4 min and the phagosome-containing post-nuclear supernatants were transferred to a V-bottom 96-well plate. The enriched phagosomes were washed with PBS containing 1% (vol/vol) BSA and stained with different primary antibodies overnight at 4 °C. The next day, the samples were incubated with appropriate secondary antibodies for 45 min on ice. Phagosomes were analysed by flow cytometry using a CytoFlex flow cytometer. The data were analysed using FlowJo software.

### Bead saporin–puromycin phagosomal escape assay

The bead saporin–puromycin was performed in cell culture medium lacking β-mercaptoethanol (βM-free) to avoid reduction of the conjugating disulfide bond on the saporin beads (see above for bead preparation protocol). MutuDCs were collected, washed once in PBS and seeded in a 96-well U-bottom plate with 5 × 10^5^ cells per well in 100 µL βM-free cell culture medium. Saporin beads were added at a beads:cells ratio of 10:1 in 50 µL βM-free medium. For drug-treated cells, the βM-free medium was supplemented with a final concentration of 0.5 µM bafilomycin A1, 10 μM DAPT or 10 μM Z-FA-FMK. Cells were incubated with beads at 37 °C for the indicated times. Then, puromycin was added at 10 µg/mL for 30 min, and cells were subsequently washed in ice-cold PBS. Non-internalised beads were stained on ice with a rabbit αOvalbumin antibody (1:200) for 30 min followed by an αRabbit-AF455 antibody (1:1000) for 30 min. After labelling dead cells with ViaKrome 808 (1:1000) for 10 min on ice, cells were fixed and permeabilised using the BD Cytofix/Cytoperm and Perm/Wash buffers. Puromycin incorporation was determined by staining with an αPuromycin-AF647 antibody (1:200) in Perm/Wash buffer for 45 min on ice. Cells were analysed by flow cytometry using a CytoFlex flow cytometer. The data were analysed using FlowJo software.

### pHrodo assay

For pHrodo assays with MutuDCs and BMDCs, the cells were pulsed with beads as described in the phagoFACS assay protocol, but using pHrodo-labelled beads (see above for bead preparation protocol). For pHrodo assays with splenic DCs, the pulse was adjusted to 15 min for 37 °C to allow comparison to a previous study (Savina et al, 2009). The chase was performed at 37 °C for different periods of time and stopped by adding ice-cold PBS. For drug-treated cells, the medium was supplemented with a final concentration of 0.5 µM bafilomycin A1 or 5 μM DPI. Non-internalised beads and dead cells were labelled in PBS containing 1% (vol/vol) BSA with a rabbit anti-OVA antibody for 30 min on ice, followed by a staining with donkey anti-rabbit-AF647 and ViaKrome 808 for 30 min on ice. For pHrodo assays with Flt3L-BMDCs and splenic DCs, non-specific antibody binding was blocked using αCD16/CD32 prior to staining. The staining also included antibodies against surface markers. Stained cells were immediately analysed on a CytoFlex flow cytometer using a chilled sample stage. The data were analysed using FlowJo software.

### Western blotting

For western blotting of drug-treated cells, MutuDCs were seeded into a six-well dish at 2 × 10^6^ cells/well in 2 mL of cell culture media. The next day, cells were incubated with inhibitors as indicated in the relevant figure legends. Drug-treated or untreated cells were pelleted and washed once in ice-cold PBS. Pellets were lysed in RIPA buffer supplemented with 1× protease inhibitor cocktail for 20 min at 4 °C while shaking at 800 rpm. Insoluble material was pelleted by centrifugation at 20,000× *g* for 10 min. Supernatants were mixed with NuPAGE LDS Sample Buffer and heated at 70 °C for 10 min in the presence or absence of 1× Bolt reducing agent. Samples were run in MOPS buffer on NuPAGE 4–12% Bis-Tris gels at 150 V for 60 min and transferred onto nitrocellulose membrane using an iBlot1 system. Loading was controlled by Ponceau staining. Membranes were then blocked by incubation with blocking buffer (PBS containing 5% milk 0.1% Tween-20) for 1 h at room temperature. Primary antibodies were diluted in blocking buffer and incubated at 4 °C overnight. After washing three times with PBS containing 0.1% Tween-20, membranes were incubated with HRP-conjugated secondary antibodies diluted in blocking buffer for 1 h at room temperature. Membranes were again washed three times with PBS containing 0.1% Tween-20 and then incubated with ECL reagent for 10 min. The resulting signal was detected using X-ray films with varying exposure times.

### Identification of cleavage sites from mass-spectrometry data

Proteomic data from (Rodríguez-Silvestre et al, 2023) were used for the identification of cleavage sites in perforin-2. Briefly, peptide fragments resulting from other cleavage events than the in-solution digest with LysC and trypsin were identified by setting the enzyme mode to semi-specific. Three control samples from the experiment with small molecules and three NTC control samples from the experiment with AEP^KO^ lines were used for analysis. Non-tryptic peptides were filtered for those with LFQ intensity >25 in each of the six replicates (see also Dataset EV1a).

### Identification of the predicted cleavage sites

Mouse perforin-2 sequence was used for analysis using The Eukaryotic Linear Motif resource, 2024 update (version 2.6.1) (https://elm.eu.org/) (Kumar et al, 2024).

### Structural analyses

Molecular graphics and analyses performed with UCSF ChimeraX (version 1.8) (Meng et al, 2023). Schematics in Fig. 1 were created using a model of the full-length pre-pore monomer from pdb 6SB3 (Ni et al, 2020), which was aligned with the Alphafold model of perforin-2, AF-A1L314-F1 (Jumper et al, 2021) using the matchmaker function, and the TMD was manually rotated to place into the membrane on which the pre-pore sits (residues 643–713 are shown from the Alphafold structure). Full-length trans pore was modelled in a similar way using pdb 8AD1 (Yu et al, 2022) (residues 600-713 are shown from the AlphaFold structure).

To model the pre-pore to cis pore transition in Fig. 6A, we used previously published structures pdb 6sb3 (chains A-C) and 6sb5 (chain A) as the starting point (Ni et al, 2020). To model the TMD and cytosolic tail we used the Alphafold model modified as above. First, the matchmaker function with default settings was used to align the three copies of the Alphafold model to 6sb3/A, B, and C. Next, the P2 domain from 6sb5 (A:374-577) was aligned to 6sb3/C:374-577 using the matchmaker function with default settings. Finally, residues 17-579 in 6sb3/C, and residues 1–642 in each of the three Alphafold models were deleted.

### Statistical analysis

Details of the statistical analysis are provided in the Figure Legends. Plots were generated using GraphPad Prism version 10.6.0 for Mac (GraphPad Software, La Jolla, California, USA).

## Supplementary information

Appendix

Table EV1

Table EV2

Table EV3

Peer Review File

Dataset EV1

Source data Fig. 2

Source data Fig. 3

Source data Fig. 4

Source data Fig. 5

Source data Fig. 6

Expanded View Figures

## Data Availability

No additional datasets are deposited, and all data are included with the manuscript. The source data of this paper are collected in the following database record: biostudies:S-SCDT-10_1038-S44318-026-00903-z.

## References

[CR1] Abrahamsen HL, Sanford TC, Collamore CE, Johnstone BA, Coyne MJ, García-Bayona L, Christie MP, Evans JC, Farrand AJ, Flores K et al (2024) Distant relatives of a eukaryotic cell-specific toxin family evolved a complement-like mechanism to kill bacteria. Nat Commun 15:5028 10.1038/s41467-024-49103-5 PMC11169675 38866748

[CR2] Citarella A, Micale N (2020) Peptidyl fluoromethyl ketones and their applications in medicinal chemistry. Molecules 25:4031 10.3390/molecules25174031 PMC7504820 32899354

[CR3] Devant P, Kagan JC (2023) Molecular mechanisms of gasdermin D pore-forming activity. Nat Immunol 24:1064–1075 10.1038/s41590-023-01526-w PMC12379974 37277654

[CR4] Ding J, Wang K, Liu W, She Y, Sun Q, Shi J, Sun H, Wang D-C, Shao F (2016) Pore-forming activity and structural autoinhibition of the gasdermin family. Nature 535:111–116 10.1038/nature18590 27281216

[CR5] Dunkelberger JR, Song W-C (2010) Complement and its role in innate and adaptive immune responses. Cell Res 20:34–50 10.1038/cr.2009.139 20010915

[CR6] Dunstone MA, Tweten RK (2012) Packing a punch: the mechanism of pore formation by cholesterol dependent cytolysins and membrane attack complex/perforin-like proteins. Curr Opin Struct Biol 22:342–349 10.1016/j.sbi.2012.04.008 PMC3383384 22658510

[CR7] Ebrahimnezhaddarzi S, Bird CH, Allison CC, Tuipulotu DE, Kostoulias X, Macri C, Stutz MD, Abraham G, Kaiserman D, Pang SS et al (2022) Mpeg1 is not essential for anti-bacterial or anti-viral immunity, but is implicated in antigen presentation. Immunol Cell Biol 100:529–546 10.1111/imcb.12554 PMC9545170 35471730

[CR8] Fields KA, McCormack R, de Armas LR, Podack ER (2013) Perforin-2 restricts growth of Chlamydia trachomatis in macrophages. Infect Immun 81:3045–3054 10.1128/IAI.00497-13 PMC3719555 23753625

[CR9] Funamoto S, Sasaki T, Ishihara S, Nobuhara M, Nakano M, Watanabe-Takahashi M, Saito T, Kakuda N, Miyasaka T, Nishikawa K et al (2013) Substrate ectodomain is critical for substrate preference and inhibition of γ-secretase. Nat Commun 4:2529 10.1038/ncomms3529 PMC3826621 24108142

[CR10] Hara H, Friedlander RM, Gagliardini V, Ayata C, Fink K, Huang Z, Shimizu-Sasamata M, Yuan J, Moskowitz MA (1997) Inhibition of interleukin 1β converting enzyme family proteases reduces ischemic and excitotoxic neuronal damage. Proc Natl Acad Sci USA 94:2007–2012 10.1073/pnas.94.5.2007 PMC20033 9050895

[CR11] Hildner K, Edelson BT, Purtha WE, Diamond M, Matsushita H, Kohyama M, Calderon B, Schraml BU, Unanue ER, Diamond MS et al (2008) Batf3 deficiency reveals a critical role for CD8alpha+ dendritic cells in cytotoxic T cell immunity. Science 322:1097–1100 10.1126/science.1164206 PMC2756611 19008445

[CR12] Hoffmann E, Kotsias F, Visentin G, Bruhns P, Savina A, Amigorena S (2012) Autonomous phagosomal degradation and antigen presentation in dendritic cells. Proc Natl Acad Sci USA 109:14556–14561 10.1073/pnas.1203912109 PMC3437883 22908282

[CR13] House IG, House CM, Brennan AJ, Gilan O, Dawson MA, Whisstock JC, Law RH, Trapani JA, Voskoboinik I (2017) Regulation of perforin activation and pre-synaptic toxicity through C-terminal glycosylation. EMBO Rep 18:1775–1785 10.15252/embr.201744351 PMC5623865 28808112

[CR14] Huotari J, Helenius A (2011) Endosome maturation. EMBO J 30:3481–3500 10.1038/emboj.2011.286 PMC3181477 21878991

[CR15] Ivanova ME, Lukoyanova N, Malhotra S, Topf M, Trapani JA, Voskoboinik I, Saibil HR (2022) The pore conformation of lymphocyte perforin. Sci Adv 8:eabk3147 10.1126/sciadv.abk3147 PMC8836823 35148176

[CR16] Jiao F, Dehez F, Ni T, Yu X, Dittman JS, Gilbert R, Chipot C, Scheuring S (2022) Perforin-2 clockwise hand-over-hand pre-pore to pore transition mechanism. Nat Commun 13:5039 10.1038/s41467-022-32757-4 PMC9418332 36028507

[CR17] Jiao F, Ruan Y, Scheuring S (2021) High-speed atomic force microscopy to study pore-forming proteins. Methods Enzymol 649:189–217 10.1016/bs.mie.2021.01.033 33712187

[CR18] Jumper J, Evans R, Pritzel A, Green T, Figurnov M, Ronneberger O, Tunyasuvunakool K, Bates R, Žídek A, Potapenko A et al (2021) Highly accurate protein structure prediction with AlphaFold. Nature 596:583–589 10.1038/s41586-021-03819-2 PMC8371605 34265844

[CR19] Kopan R, Ilagan MXG (2004) γ-Secretase: proteasome of the membrane? Nat Rev Mol Cell Biol 5:499–504 10.1038/nrm1406 15173829

[CR20] Krawczyk PA, Laub M, Kozik P (2020) To kill but not be killed: controlling the activity of mammalian pore-forming proteins. Front Immunol 11:601405 10.3389/fimmu.2020.601405 PMC7691655 33281828

[CR21] Kumar M, Michael S, Alvarado-Valverde J, Zeke A, Lazar T, Glavina J, Nagy-Kanta E, Donagh JM, Kalman ZE, Pascarelli S et al (2024) ELM—the Eukaryotic Linear Motif resource—2024 update. Nucleic Acids Res 52:D442–D455 10.1093/nar/gkad1058 PMC10767929 37962385

[CR22] Law RHP, Lukoyanova N, Voskoboinik I, Caradoc-Davies TT, Baran K, Dunstone MA, D’Angelo ME, Orlova EV, Coulibaly F, Verschoor S et al (2010) The structural basis for membrane binding and pore formation by lymphocyte perforin. Nature 468:447–451 10.1038/nature09518 21037563

[CR23] Lichtenthaler SF, Lemberg MK, Fluhrer R (2018) Proteolytic ectodomain shedding of membrane proteins in mammals—hardware, concepts, and recent developments. EMBO J 37:e99456 10.15252/embj.201899456 PMC6068445 29976761

[CR24] Lopez-Hernandez FJ, Ortiz MA, Bayon Y, Piedrafita FJ (2003) Z-FA-fmk inhibits effector caspases but not initiator caspases 8 and 10, and demonstrates that novel anticancer retinoid-related molecules induce apoptosis via the intrinsic pathway. Mol Cancer Ther 2:255–263 12657720

[CR25] Mantegazza AR, Savina A, Vermeulen M, Pérez L, Geffner J, Hermine O, Rosenzweig SD, Faure F, Amigorena S (2008) NADPH oxidase controls phagosomal pH and antigen cross-presentation in human dendritic cells. Blood 112:4712–4722 10.1182/blood-2008-01-134791 PMC2597138 18682599

[CR26] Marini G, Poland B, Leininger C, Lukoyanova N, Spielbauer D, Barry JK, Altier D, Lum A, Scolaro E, Ortega CP et al (2023) Structural journey of an insecticidal protein against western corn rootworm. Nat Commun 14:4171 10.1038/s41467-023-39891-7 PMC10344926 37443175

[CR27] Marraco SAF, Grosjean F, Duval A, Rosa M, Lavanchy C, Ashok D, Haller S, Otten LA, Steiner Q-G, Descombes P et al (2012) Novel murine dendritic cell lines: a powerful auxiliary tool for dendritic cell research. Front Immunol 3:331 10.3389/fimmu.2012.00331 PMC3491238 23162549

[CR28] McCormack RM, de Armas LR, Shiratsuchi M, Fiorentino DG, Olsson ML, Lichtenheld MG, Morales A, Lyapichev K, Gonzalez LE, Strbo N et al (2015) Perforin-2 is essential for intracellular defense of parenchymal cells and phagocytes against pathogenic bacteria. eLife 4:e06508 10.7554/eLife.06508 PMC4626811 26402460

[CR29] McCormack R, Podack ER (2015) Perforin-2/Mpeg1 and other pore-forming proteins throughout evolution. J Leukoc Biol 98:761–768 10.1189/jlb.4MR1114-523RR PMC4600061 26307549

[CR30] Meng EC, Goddard TD, Pettersen EF, Couch GS, Pearson ZJ, Morris JH, Ferrin TE (2023) UCSF ChimeraX: tools for structure building and analysis. Protein Sci 32:e4792 10.1002/pro.4792 PMC10588335 37774136

[CR31] Michlits, Jude G, Hinterndorfer J, Almeida M, de M, Vainorius G, Hubmann M, Neumann T, Schleiffer A, Burkard TR, Fellner M et al (2020) Multilayered VBC score predicts sgRNAs that efficiently generate loss-of-function alleles. Nat Methods 17:708–716 10.1038/s41592-020-0850-8 32514112

[CR32] Netting DJ, López-Haber C, Hutchins Z, Martina JA, Puertollano R, Mantegazza AR (2025) The lysosomal carrier SLC29A3 supports antibacterial signaling, and promotes autophagy by activating TRPML1 in murine dendritic cells. Proc Natl Acad Sci USA 122:e2511539122 10.1073/pnas.2511539122 PMC12685069 41284877

[CR33] Ni T, Jiao F, Yu X, Aden S, Ginger L, Williams SI, Bai F, Pražák V, Karia D, Stansfeld P et al (2020) Structure and mechanism of bactericidal mammalian perforin-2, an ancient agent of innate immunity. Sci Adv 6:eaax8286 10.1126/sciadv.aax8286 PMC6989145 32064340

[CR34] Nielsen K, Boston RS (2001) Ribosome-inactivating proteins: a plant perspective. Annu Rev Plant Physiol Plant Mol Biol 52:785–816 10.1146/annurev.arplant.52.1.785 11337416

[CR35] Pang SS, Bayly-Jones C, Radjainia M, Spicer BA, Law RHP, Hodel AW, Parsons ES, Ekkel SM, Conroy PJ, Ramm G et al (2019) The cryo-EM structure of the acid activatable pore-forming immune effector Macrophage-expressed gene 1. Nat Commun 10:4288 10.1038/s41467-019-12279-2 PMC6753088 31537793

[CR36] Rodríguez-Silvestre P, Laub M, Krawczyk PA, Davies AK, Schessner JP, Parveen R, Tuck BJ, McEwan WA, Borner GHH, Kozik P (2023) Perforin-2 is a pore-forming effector of endocytic escape in cross-presenting dendritic cells. Science 380:1258–1265 10.1126/science.adg8802 PMC7614779 37347855

[CR37] Savina A, Jancic C, Hugues S, Guermonprez P, Vargas P, Moura IC, Lennon-Duménil A-M, Seabra MC, Raposo G, Amigorena S (2006) NOX2 controls phagosomal pH to regulate antigen processing during crosspresentation by dendritic cells. Cell 126:205–218 10.1016/j.cell.2006.05.035 16839887

[CR38] Savina A, Peres A, Cebrian I, Carmo N, Moita C, Hacohen N, Moita LF, Amigorena S (2009) The small GTPase Rac2 controls phagosomal alkalinization and antigen crosspresentation selectively in CD8+ dendritic cells. Immunity 30:544–555 10.1016/j.immuni.2009.01.013 19328020

[CR39] Schmidt EK, Clavarino G, Ceppi M, Pierre P (2009) SUnSET, a nonradioactive method to monitor protein synthesis. Nat Methods 6:275–277 10.1038/nmeth.1314 19305406

[CR40] Serna M, Giles JL, Morgan BP, Bubeck D (2016) Structural basis of complement membrane attack complex formation. Nat Commun 7:10587 10.1038/ncomms10587 PMC4743022 26841837

[CR41] Uellner R, Zvelebil MJ, Hopkins J, Jones J, MacDougall LK, Morgan BP, Podack E, Waterfield MD, Griffiths GM (1997) Perforin is activated by a proteolytic cleavage during biosynthesis which reveals a phospholipid-binding C2 domain. EMBO J 16:7287–7296 10.1093/emboj/16.24.7287 PMC1170329 9405358

[CR42] Vind AC, Genzor AV, Bekker-Jensen S (2020) Ribosomal stress-surveillance: three pathways is a magic number. Nucleic Acids Res 48:10648–10661 10.1093/nar/gkaa757 PMC7641731 32941609

[CR43] Voskoboinik I, Thia M-C, Fletcher J, Ciccone A, Browne K, Smyth MJ, Trapani JA (2005) Calcium-dependent plasma membrane binding and cell lysis by perforin are mediated through its C2 domain: a critical role for aspartate residues 429, 435, 483, and 485 but not 491. J Biol Chem 280:8426–8434 10.1074/jbc.M413303200 15576364

[CR44] Yu X, Ni T, Munson G, Zhang P, Gilbert RJC (2022) Cryo-EM structures of perforin-2 in isolation and assembled on a membrane suggest a mechanism for pore formation. EMBO J 41:e111857 10.15252/embj.2022111857 PMC9713709 36245269

