# Supplementary material for: Anchoring of perforin-2 via the transmembrane domain is required for antigen escape into the cytosol
Source: EMBO J. 2026 Aug 19;45(19):6759–81. doi: 10.1038/s44318-026-00903-z (PMC13631362; doi:10.1038/s44318-026-00903-z)
Supplement: Supplementary file 1 [file 44318_2026_903_MOESM12_ESM.pdf]

Expanded View Figures

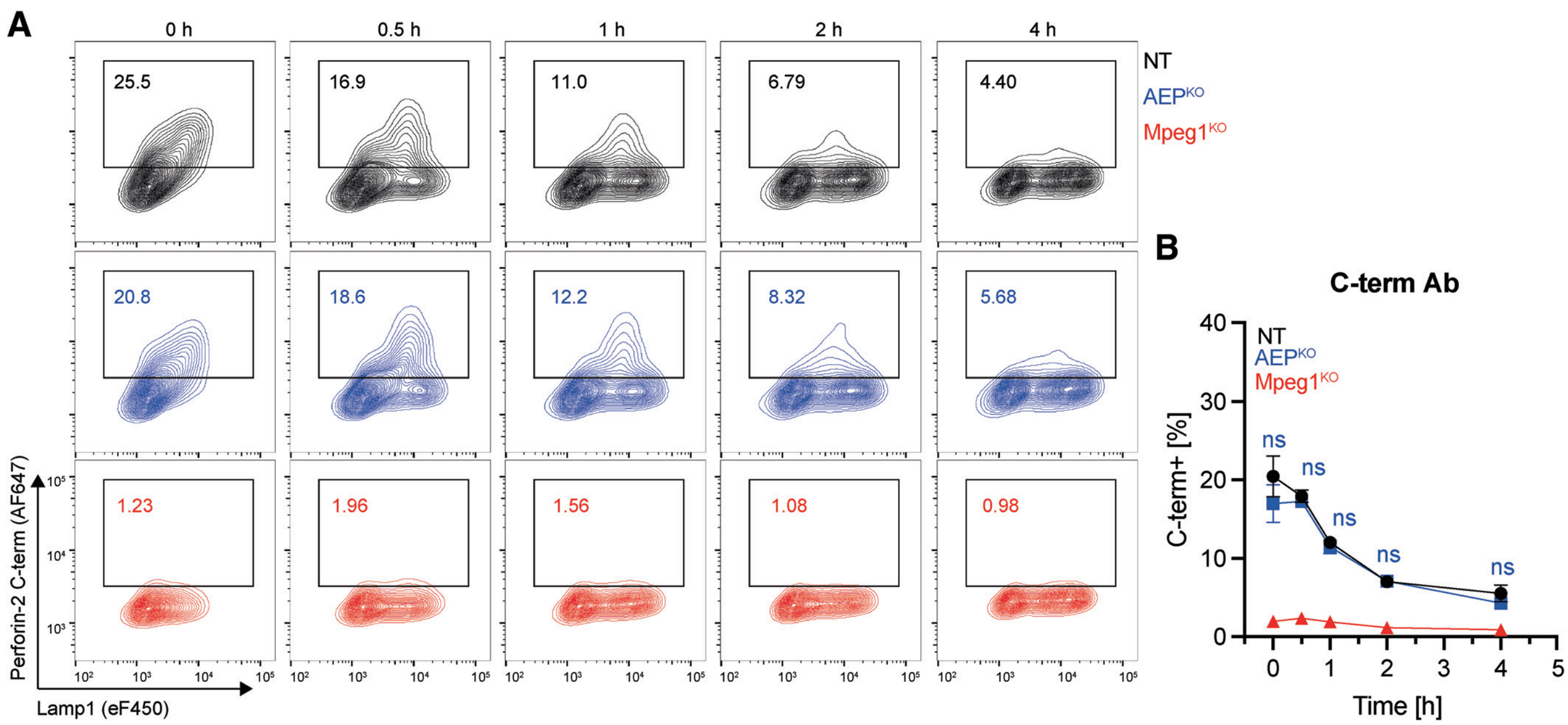

Figure EV1. Perforin-2 recruitment to phagosomes in control, AEP^{KO}, and Mpeg1^{KO} cells.

(**A**, **B**) PhagoFACS assay as in Fig. 2E,F showing staining with αC-term (**A**) and % of αC-term^{+} phagosomes (**B**). Data represent mean and SEM of $n = 3$ independent experiments. ns, not significant using a two-way ANOVA with Dunnett's multiple comparisons test versus NT MutuDCs.

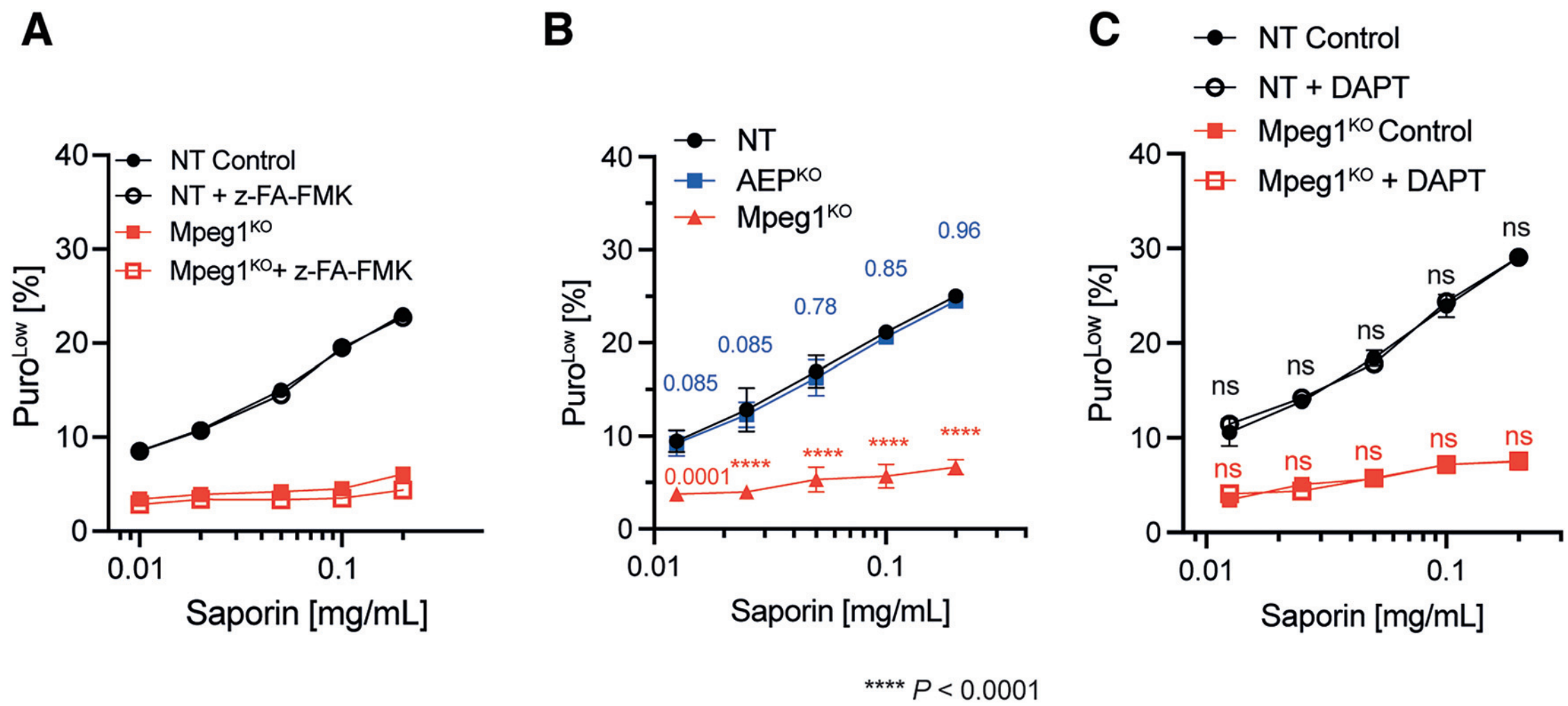

Figure EV2. Efficiency of antigen escape in MutuDCs monitored by the saporin-puromycin assay.

(**A**) Saporin–puromycin assay in NT or Mpeg1^{KO} MutuDCs pre-incubated for 16 h with Z-FA-FMK. (**B**) Saporin–puromycin assay in NT, AEP^{KO}, and Mpeg1 ^{KO} MutuDCs. See also Appendix Fig. S4C). $n = 3$ independent experiments. P values calculated using a two-way ANOVA with Dunnett's multiple comparisons test versus NT MutuDCs. (**C**) Saporin–puromycin assay in NT and Mpeg1^{KO} MutuDCs with and without DAPT. $n = 3$ independent experiments. ns, not significant using a multiple paired t test (two-stage step-up, Benjamini, Krieger and Yekutieli).

 © The Author(s)

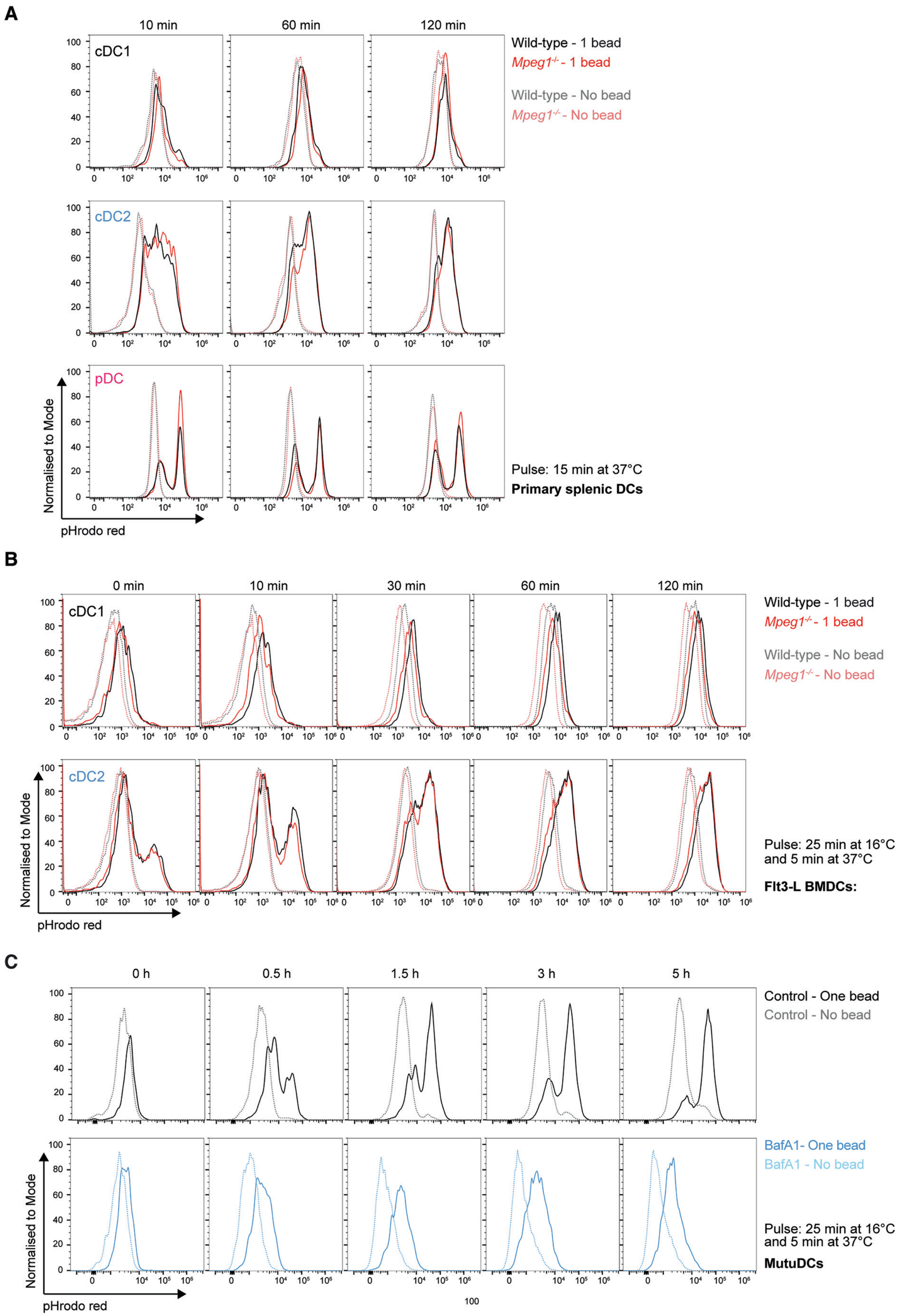

A
10 min
60 min
120 min
cDC1
cDC2
pDC
Wild-type - 1 bead
Mpeg1-/- - 1 bead
Wild-type - No bead
Mpeg1-/- - No bead
Pulse: 15 min at 37°C
Primary splenic DCs
Normalised to Mode
pHrodo red
B
0 min
10 min
30 min
60 min
120 min
cDC1
cDC2
Wild-type - 1 bead
Mpeg1-/- - 1 bead
Wild-type - No bead
Mpeg1-/- - No bead
Pulse: 25 min at 16°C
and 5 min at 37°C
Flt3-L BMDCs:
Normalised to Mode
pHrodo red
C
0 h
0.5 h
1.5 h
3 h
5 h
Control - One bead
Control - No bead
BafA1- One bead
BafA1 - No bead
Pulse: 25 min at 16°C
and 5 min at 37°C
MutuDCs
Normalised to Mode
pHrodo red
100

Figure EV3. Analysis of phagosomal pH in dendritic cells.

(**A**, **B**) pHrodo-bead assay on WT or Mpeg1$^{-/-}$ splenic DCs were (**A**) or Flt3L-derived BMDCs (**B**). See also Appendix Fig. S6. Cells were pulsed with beads for 15 min at 37 °C and chased for the indicated time. Data representative of $n = 2$ independent experiments. (**C**) pHrodo-bead assay on MutuDCs in the presence or absence of 0.5 μM BafA1. Data representative of $n = 5$ independent experiments.

© The Author(s)

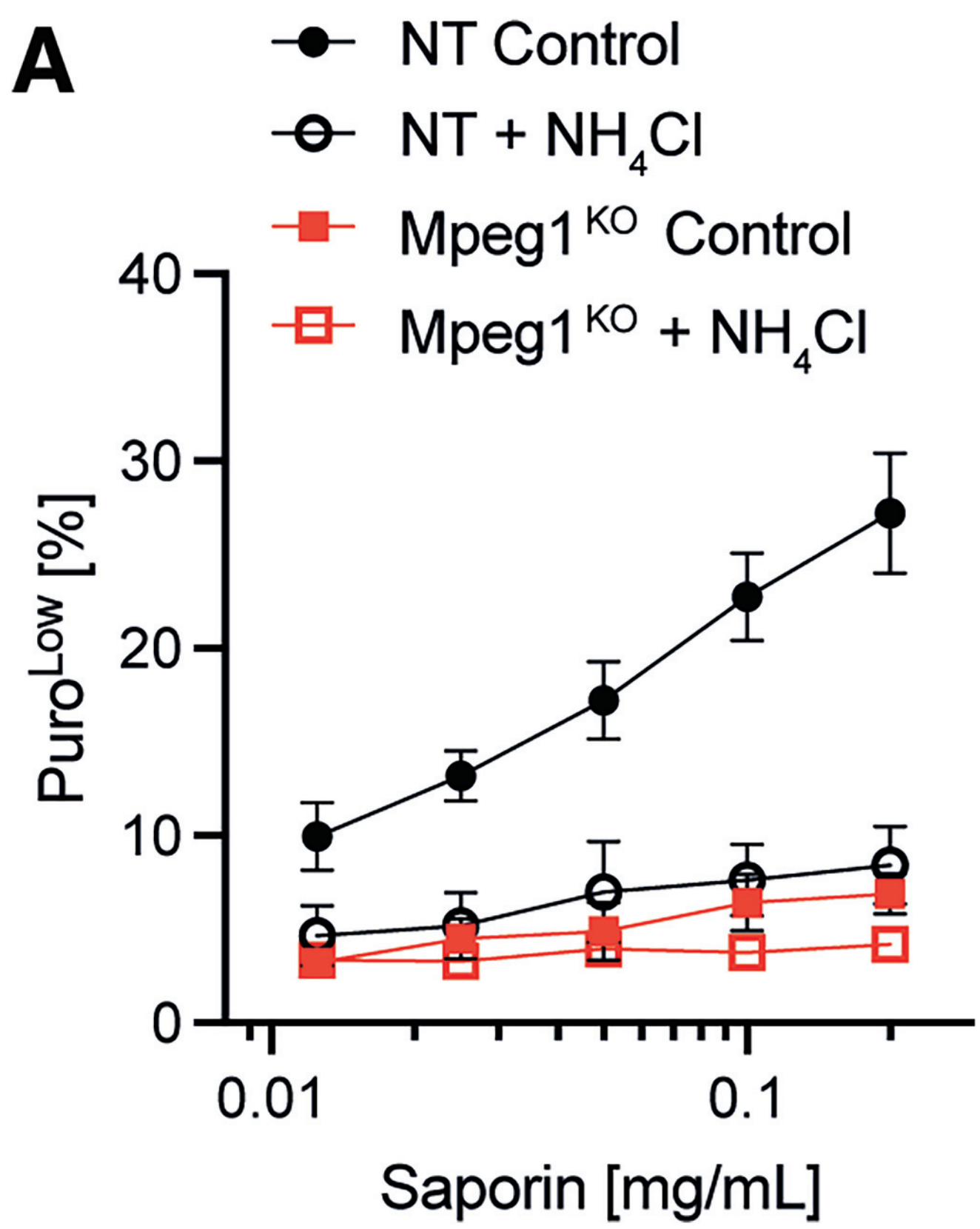

Figure EV4. Efficiency of antigen escape in MutuDCs treated with NH_2Cl to neutralise phagosomal pH.

(A) Saporin-puromycin assay in NT and Mpeg1^{KO} MutuDCs with and without NH_4Cl. $n = 3$ independent experiments.
