# Supplementary material for: Anchoring of perforin-2 via the transmembrane domain is required for antigen escape into the cytosol
Source: EMBO J. 2026 Aug 19;45(19):6759–81. doi: 10.1038/s44318-026-00903-z (PMC13631362; doi:10.1038/s44318-026-00903-z)
Supplement: Supplementary file 2 [file 44318_2026_903_MOESM1_ESM.pdf]

Appendix for Anchoring of perforin-2 via the transmembrane domain is required for antigen escape into the cytosol

Table of Contents

Appendix Figure S1. Ponceau-stained blots for Western Blots included in the manuscript. 2
Appendix Figure S2. Gating strategy for the phagoFACS assays. 3
Appendix Figure S3. Validation of the knockout cell lines. 4
Appendix Figure S4. Saporin-puromycin phagosomal escape assay. 5
Appendix Figure S5. Promiscuous cleavage in the EGF domain is not required for saporin escape. 6
Appendix Figure S6. pHrodo assay in primary and Flt3L-derived DCs. 7
Appendix Figure S7. Gating strategy for saporin assay with the perforin-2 CTT mutants. 8

Appendix Figure S1

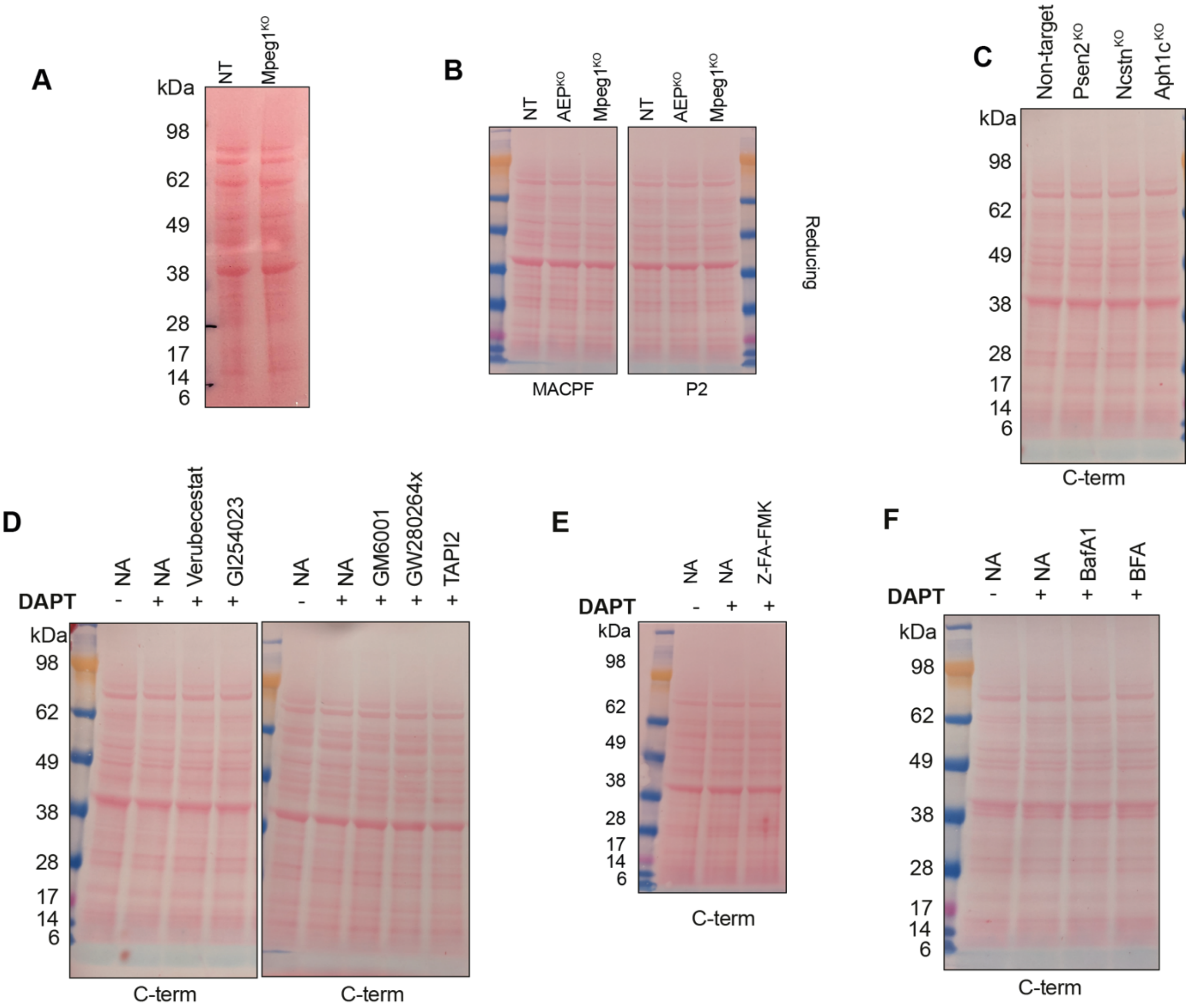

Appendix Figure S1. Ponceau-stained blots for Western Blots included in the manuscript.

A Loading control for Figure 2B
B Loading control for Figure 2C
C Loading control for Figure 3B
D Loading control for Figure 4C
E Loading control for Figure 4E
F Loading control for Figure 4D

Appendix Figure S2

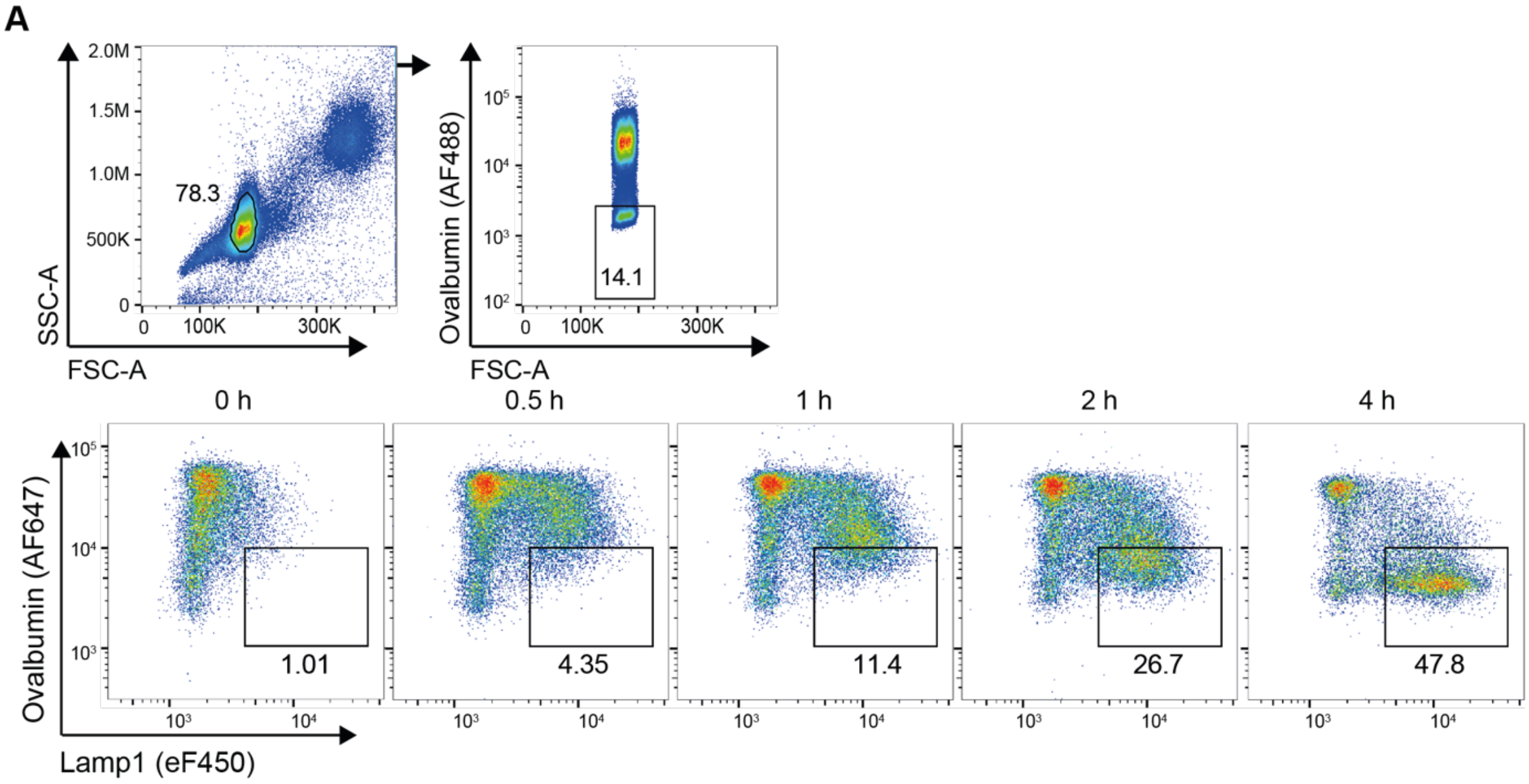

Appendix Figure S2. Gating strategy for the phagoFACS assays.

A Gating strategy for analysis of phagosomes isolated from MutuDCs. The cells were pulsed with OVA beads for 25 min at 16°C followed by 5 min at 37°C and either immediately placed on ice (0 h) or chased for the indicated time. Isolated phagosomes were stained with antibodies against ovalbumin and Lamp1. The gate in the bottom panel indicates mature phagosomes. n = 5 independent experiments.

Appendix Figure S3

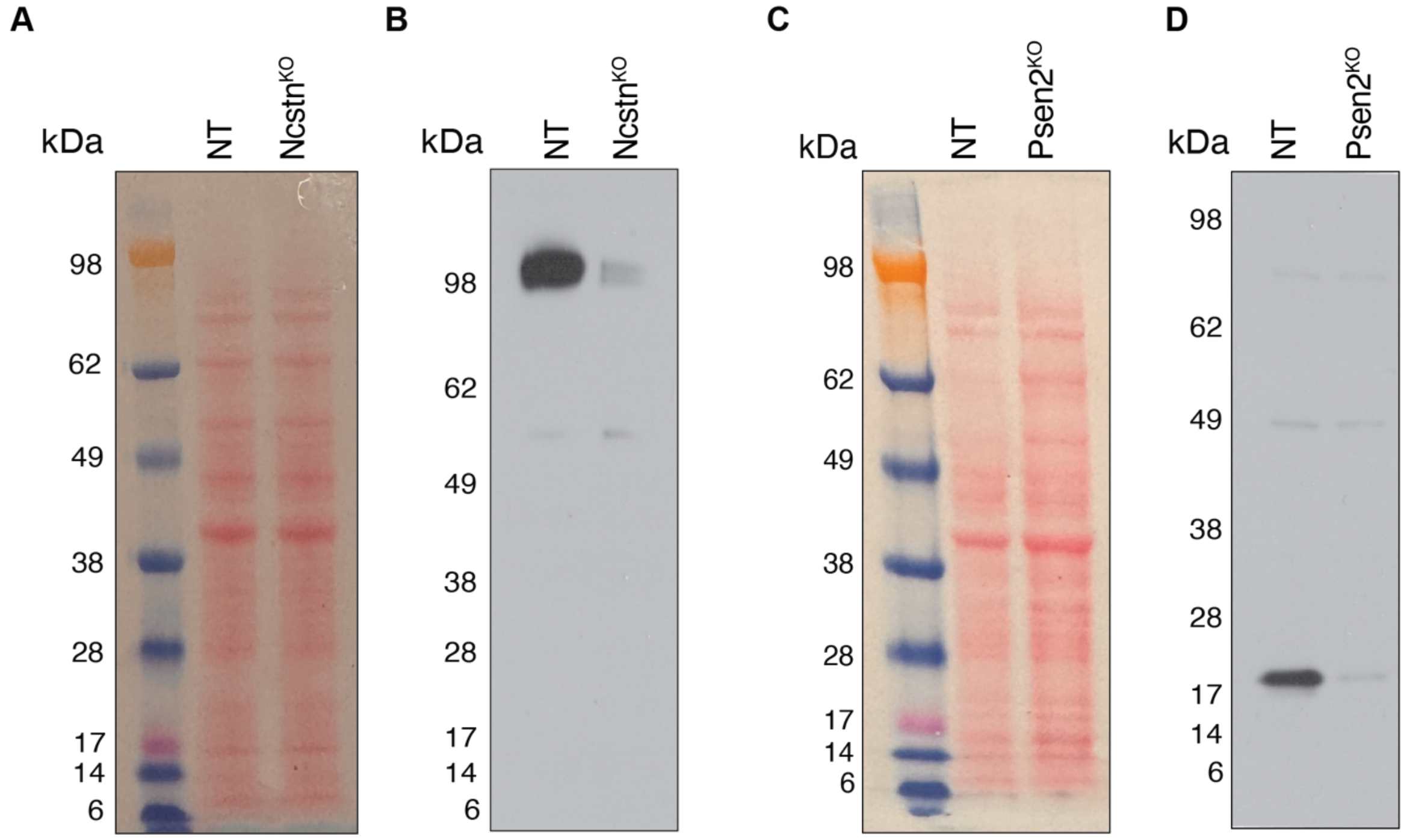

Appendix Figure S3. Validation of the knockout cell lines.

A,B Loading control (A) and immunoblot of control and Ncstn^{KO} MutuDCs with the αNcstn antibody.

C,D Loading control (C) and immunoblot (D) of control and Psen2^{KO} MutuDCs with the αPsen2 antibody.

Appendix Figure S4

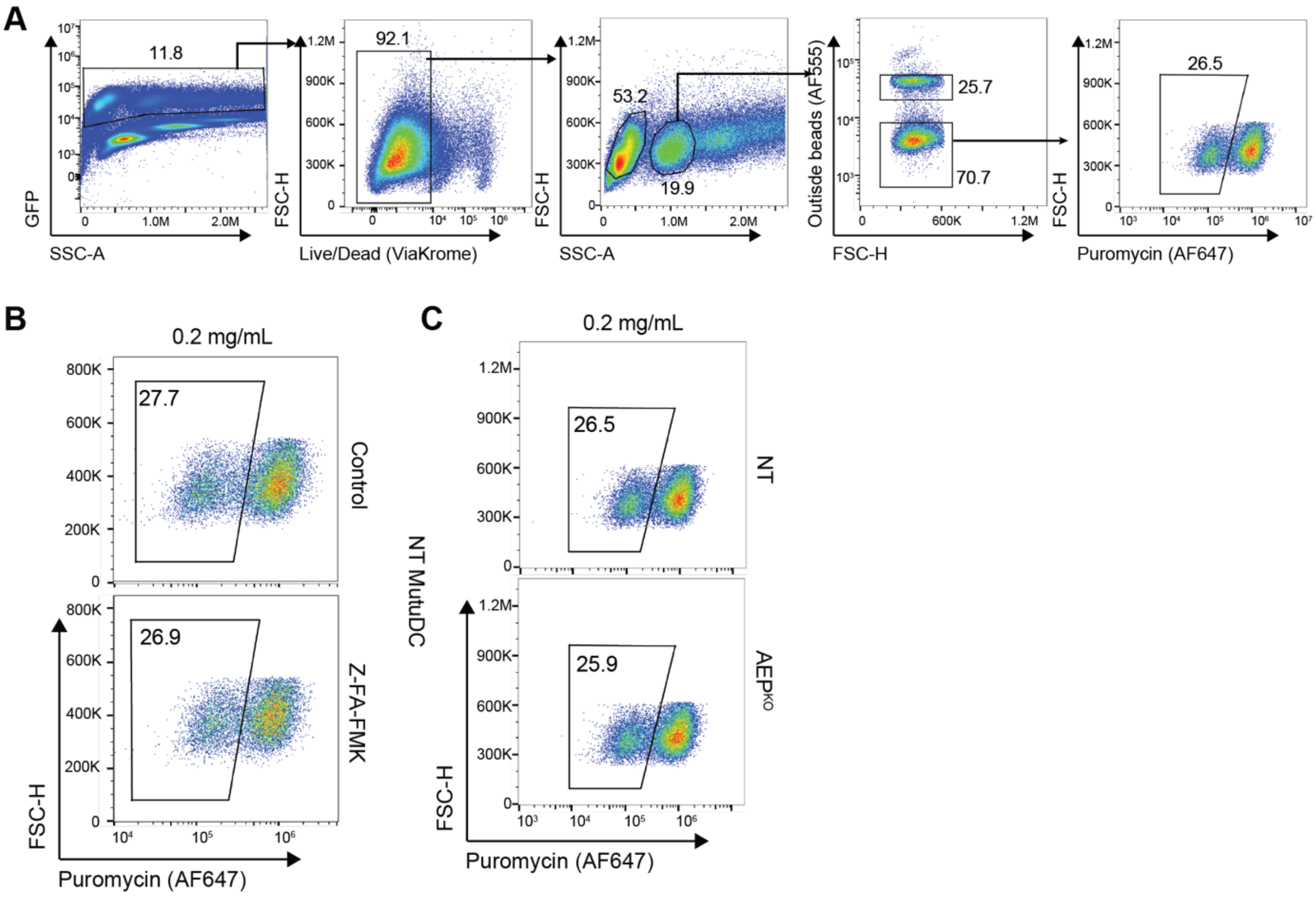

Appendix Figure S4. Saporin-puromycin phagosomal escape assay.

A Gating strategy for the saporin-puromycin assay to monitor antigen escape from phagosomes (NT sample from panel C is used as an example).
B,C Representative flow plots from the saporin-puromycin assay performed in the presence and absence of Z-FA-FMK (with 1 h pre-incubation) (B) or in NT and AEP^{KO} MutuDCs (C).

Appendix Figure S5

A

z-FA-FMK	Cysteine proteases
Leupeptin	Proteases inhibitor
E64D	Cysteine proteases-Cathepsin and Calpain
CA-074	Cathepsin B inhibitor
CA-074-Me	Cathepsin B and L inhibitor

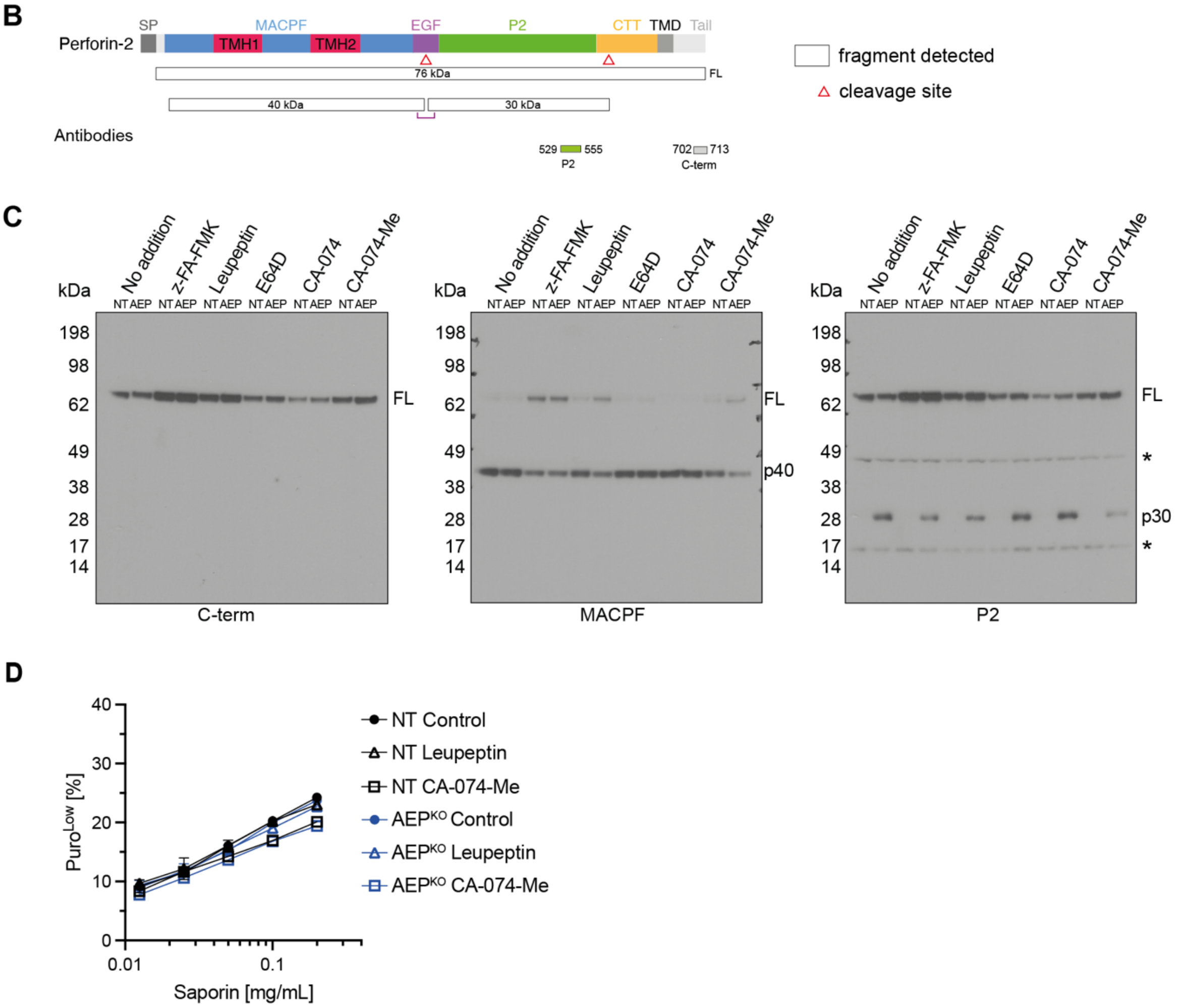

Appendix Figure S5. Promiscuous cleavage in the EGF domain is not required for saporin escape.

A Small-molecule inhibitors and their targets.

B Diagram of perforin-2 fragments relevant for the immunoblots in this Figure.

C Immunoblot of NT or AEP^{KO} MutuDCs untreated or incubated with Z-FA-FMK (10 µM), Leupeptin (100 µM), E64D (10 µM), CA-074 (50 µM) or CA-0740-Me (50 µM) for 6 hours. Immunoblot under reducing conditions using the αC-term, αMACPF or αP2 antibodies. Leupeptin and CA-074-Me have a synergistic effect with AEP depletion, suggesting that cleavage in the EGF linker is promiscuous and can be executed by either AEP or cathepsins. n = 2 independent experiments.

D Quantification of PuroLow cells in saporin-puromycin escape assay in NT and AEP^{KO} MutuDCs with and without treatment with the leupeptin or CA-074-Me. These data support the notion that cleavage in the EGF domain is not required for endocytic escape. Mean and SEM of n =2 independent experiments.

Appendix Figure S6

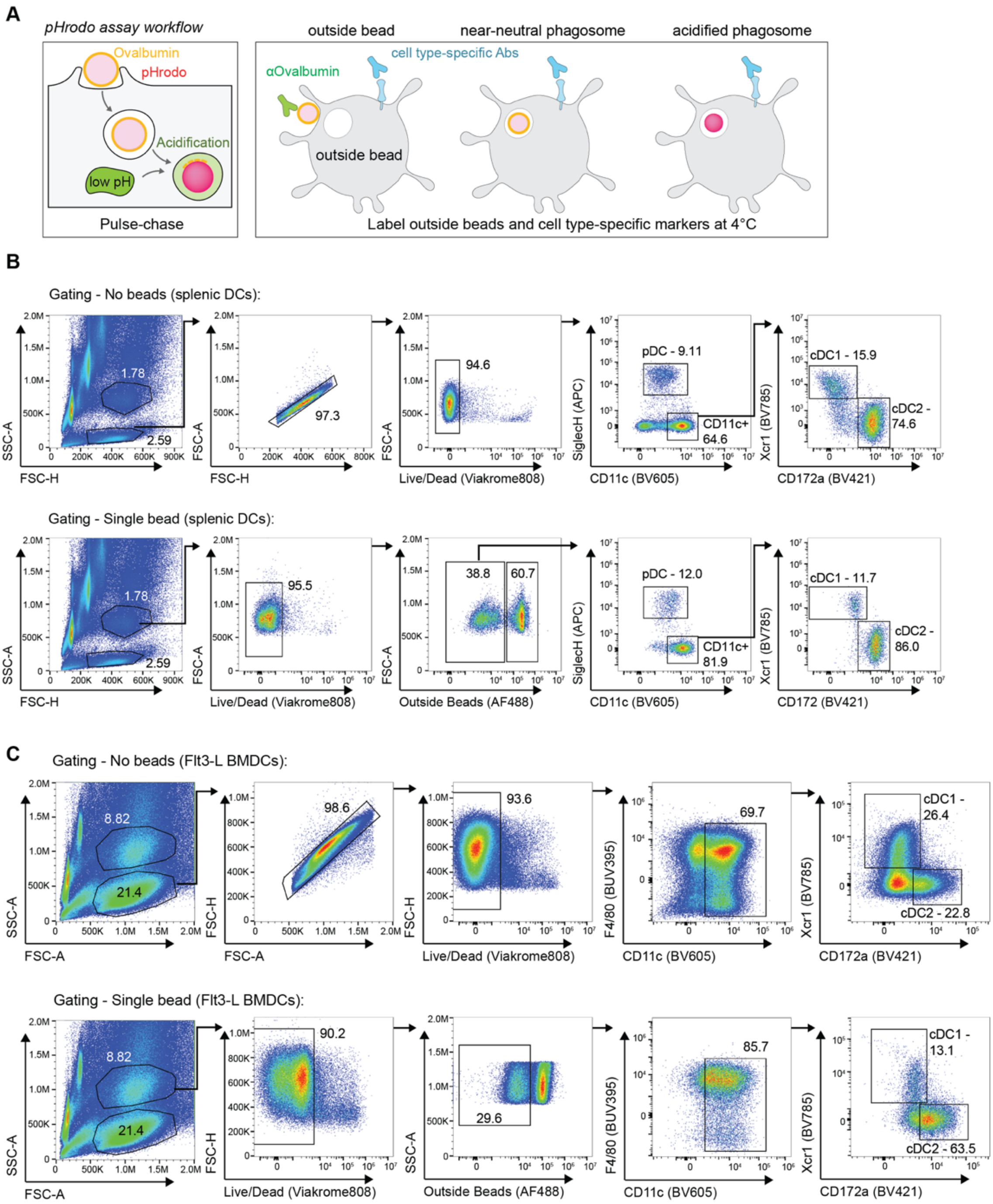

Appendix Figure S6. pHrodo assay in primary and Flt3L-derived DCs.

A Schematic representation of the pHrodo bead assay.
B Gating strategy for the pHrodo bead assay in primary splenic DCs. Two different gating strategies of the same sample are shown, which were used to identify cells with and without beads.
C Gating strategy for the pHrodo bead assay in primary Flt3L-bone marrow derived dendritic cells (Flt3-BMDCs). Two different gating strategies of the same sample are shown, which were used to identify cells with and without beads.

Appendix Figure S7

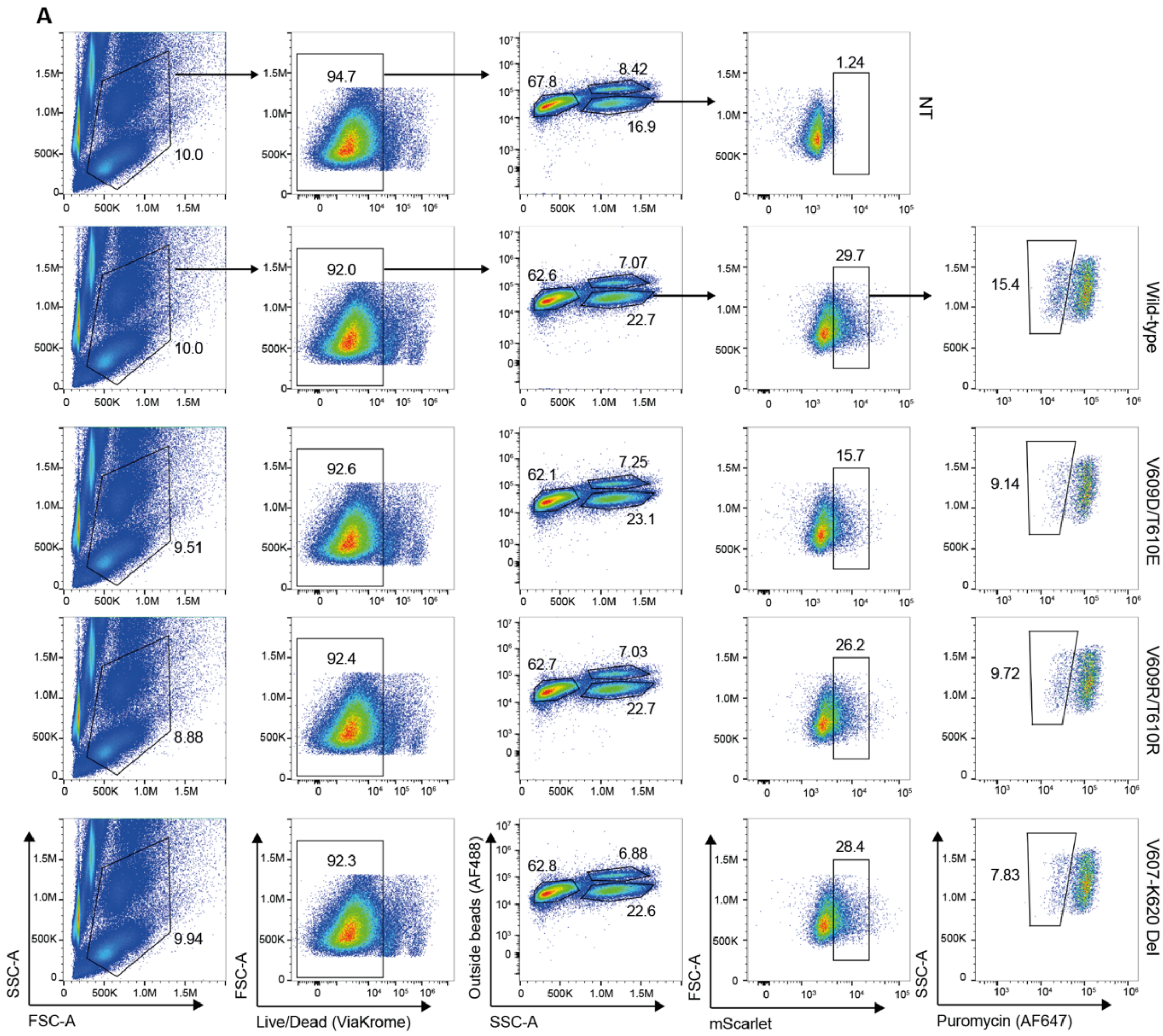

Appendix Figure S7. Gating strategy for saporin assay with the perforin-2 CTT mutants.

A Gating strategy for the saporin-puromycin assay in the indicated MutuDC lines: NT (control), and Mpeg1^{KO} MutuDC reconstituted with WT, V609D/T610E, V609R/T610R and V607-K620 Del perforin-2 mutants. mScarlet levels were used to normalise for transduction efficiency. Mean and SEM of n = 2 independent experiments.
