# Supplementary material for: Anchoring of perforin-2 via the transmembrane domain is required for antigen escape into the cytosol
Source: EMBO J. 2026 Aug 19;45(19):6759–81. doi: 10.1038/s44318-026-00903-z (PMC13631362; doi:10.1038/s44318-026-00903-z)
Supplement: Supplementary file 3 [file 44318_2026_903_MOESM5_ESM.pdf]

THE EMBO JOURNAL

Anchoring of perforin-2 via the transmembrane domain is required for antigen escape into the cytosol

Marco Laub, Ritika Chatterjee, and Patrycja Kozik

Corresponding author(s): Patrycja Kozik (pkozik@mrc-lmb.cam.ac.uk)

Review Timeline:

Submission Date:	1st Dec 25
Editorial Decision:	2nd Feb 26
Revision Received:	18th May 26
Editorial Decision:	9th Jun 26
Revision Received:	4th Aug 26
Accepted:	5th Aug 26

Editor: Hartmut Vodermaier

Transaction Report:

(Note: With the exception of the correction of typographical or spelling errors that could be a source of ambiguity, letters and reports are not edited. Depending on transfer agreements, referee reports obtained elsewhere may or may not be included in this compilation. Referee reports are anonymous unless the Referee chooses to sign their reports.)

1st Editorial Decision **2nd Feb 2026**

Dr. Patrycja Kozik
MRC Laboratory of Molecular Biology
Protein and Nucleic Acid Chemistry
Francis Crick Avenue
Cambridge
United Kingdom

2nd Feb 2026

Re: EMBOJ-2025-123194
Anchoring of perforin-2 via the transmembrane domain is required for antigen escape into the cytosol

Dear Kozik,

Thank you again for submitting your study on perforin-2 pore formation to The EMBO Journal, and my apologies for the extended duration of the review process at this time of the year. We have now finally received a complete set of reports from three expert referees, copied below for your information. Since all referees appreciate the interest and potential importance of your findings, we shall be happy to consider this work further for publication, pending satisfactory responses to a number of specific queries raised in the reviews.

Please remember that it is our policy to consider only a single round of major revision, making it important to adequately answer all comments at the time of resubmission; therefore, please do not hesitate to get back to me in case you would like to clarify/discuss any of the referees' points or plans for addressing them ahead of time. We would also be open to extending the revision deadline if that should be helpful. As always, our scooping protection will remain valid throughout the full revision period.

Detailed information on preparing, formatting and uploading a revised manuscript can be found below and in our Guide to Authors, and adhering to them as closely as possible shall greatly facilitate editorial processing upon resubmission. Thank you again for the opportunity to consider this work for The EMBO Journal, and I look forward to your revision in due time.

Yours sincerely,

Hartmut Vodermaier

Hartmut Vodermaier, PhD
Senior Editor, The EMBO Journal
h.vodermaier@embojournal.org

*** PLEASE NOTE: All revised manuscript are subject to initial checks for completeness and adherence to our formatting guidelines. Revisions may be returned to the authors and delayed in their editorial re-evaluation if they fail to comply to the following requirements. As a first step please read our guidelines for revised submissions:
https://link.springer.com/journal/44318/submission-guidelines#cms-Revised-submissions

1) Every manuscript requires a Data Availability section (even if only stating that no deposited datasets are included). Primary datasets or computer code produced in the current study have to be deposited in appropriate public repositories prior to resubmission, and reviewer access details provided in case that public access is not yet allowed.

2) Each figure legend must specify
- size of the scale bars that are mandatory for all micrograph panels
- the statistical test used to generate error bars and P-values
- the type error bars (e.g., S.E.M., S.D.)
- the number (n) and nature (biological or technical replicate) of independent experiments underlying each data point
- Figures may not include error bars for experiments with n<3; scatter plots showing individual data points should be used instead.

3) Revised manuscript text (including main tables, and figure legends for main and EV figures) has to be submitted as editable text file (e.g., .docx format). We encourage highlighting of changes (e.g., via text color) for the referees' reference.

4) Each main and each Expanded View (EV) figure should be uploaded as individual production-quality files (preferably in .eps, .tif, .jpg formats). For suggestions on figure preparation/layout, please refer to our Figure Preparation Guidelines:

https://media.springernature.com/original/springer-cms/rest/v1/content/27825798/data/v1

5) Point-by-point response letters should include the original referee comments in full together with your detailed responses to them (and to specific editor requests if applicable), and also be uploaded as editable (e.g., .docx) text files.

6) Please complete our Author Checklist, and make sure that information entered into the checklist is also reflected in the manuscript; the checklist will be available to readers as part of the Review Process File.

7) All authors listed as (co-)corresponding need to deposit, in their respective author profiles in our submission system, a unique ORCiD identifier linked to their name. Please see our Guide to Authors for detailed instructions.

8) Please note that supplementary information at EMBO Press has been superseded by the 'Expanded View' for inclusion of additional figures, tables, movies or datasets; with up to five EV Figures being typeset and directly accessible in the HTML version of the article.

9) To facilitate reproducibilty and cross-laboratory adoption of methodologies, please structure the Materials & Methods section as outlined in our guide to authors, including a completed Reagents and Tools Table.

10) Digital image enhancement is acceptable practice, as long as it accurately represents the original data and conforms to community standards. If a figure has been subjected to significant electronic manipulation, this must be clearly noted in the figure legend and/or the 'Materials and Methods' section. The editors reserve the right to request original versions of figures and the original images that were used to assemble the figure. Finally, we generally encourage uploading of numerical as well as gel/blot image source data.

At EMBO Press we ask authors to provide source data for the main manuscript figures. You will receive a separate email with instructions for providing source data with your revised manuscript, including how to upload and organize the files.

In the interest of ensuring the conceptual advance provided by the work, we recommend submitting a revision within 3 months (3rd May 2026). Please discuss the revision progress ahead of this time with the editor if you require more time to complete the revisions. Use the link below to submit your revision:

Link Not Available

Referee #1:

This study clarifies important issues about the biological roles and mechanisms of perforin-2 (PF2), an important effector in the immune response, both in attack of intracellular pathogens and the cross presentation of antigens. The proteolytic cleavage sites of PF2 are mapped and the proteases identified by the use of specific inhibitors. The finding that cleavage of the transmembrane anchor is not required for pore formation in the phagosome membrane along with the observations on the role of acidification lead to a plausible basis for the two different actions of PF2 - anchored monomers can assemble a cis pore and allow for antigen cross presentation, whereas the soluble ectodomains can assemble on a pathogen membrane as trans pores. Previously published mechanistic proposals were less plausible. Therefore this work provides an important advance in understanding of the actions of PF2 in different immune cell types.
The experiments have been carefully and thoroughly done, as far as this referee can discern.
Minor comment: The titration of saporin action is not convincing on a quantitative level, since only 2 points were measured to infer a linear (?) relationship.
Very minor comment: although the writing is generally very clear, there are 2 or 3 sentences with missing words, eg line 257.

Referee #2:

The protein perforin-2 has a central role in an important immunological process referred to as cross-presenting. While the authors have previously shown that the transmembrane protein perforin-2 can be proteolytically cleaved, the cleavage sites and the contributing proteases were previously largely unknown. More importantly, the functional relevance of proteolysis for the role of perforin-2 in cross-presenting remained entirely unclear and controversially discussed. Exactly these open questions are addressed and solved in the current manuscript. The authors demonstrate that perforin-2 undergoes shedding by acidic cathepsins, followed by intramembrane cleavage mediated by gamma-secretase. But the authors also demonstrate that both proteolytic events are not required for perforin's role in cross-presenting, but rather represent a degradation pathway. Based on the new findings, the authors propose a structure-function model for perforin's role in cross-presenting.
I am not an expert on cross-presenting and, thus, cannot fully judge the importance of the work for the perforin and cross-

presenting field.
The study is novel and interesting and largely well controlled. The following points need to be addressed to improve the manuscript and should be easy to address.

Major points:
1. Fig. 3C: the authors claim to have knocked-out subunits of gamma-secretase, but do not show proof. Provide a Western blot demonstrating the lack of the corresponding protein. This is easily feasible for Psen2 and Ncstn and may work for Aph1c as well. Alternatively, PCR may be used for Aph1c using mutation-specific primers.
2. Figs. 2, 3, 4: loading controls, such as actin, which are typically used as standard quality control for immunoblots, are entirely missing. They should be added, at least for panels 4D and E, where strong effects on full-length perforin-2 are seen in the absence of CTFs.
3. Describe in more detail how the shedding site at M599 was identified - were both peptides identified, the one ending in M599 and the one starting with amino acid 600? If not, how can the authors be sure that cleavage happens C-terminally of M599 and not further C-terminally, followed by carboxypeptidase trimming as it is frequently seen for other shedding and gamma-secretase substrates? Having a shedding site more than 50 amino acids away from the membrane is rare, as most cleavage sites are closer to the membrane. If the authors are not sure about this cleavage site, the corresponding part in the results should be phrased more carefully. Alternatively, experiments can further address this question, such as determining the N-terminus of the C-terminal fragment or testing point mutations that may abolish cleavage by cathepsins.

Minor points:
4. Line 179: The signal from the P2-loop antibody for NT peaks at 1 h, not at 2 h. Please correct.
5. Fig. 5A: describe the assay in more detail, at least in the figure legend. The assay is difficult to understand for scientists without prior knowledge of this assay.
6. Fig. 6A: describe in more detail how the structure was modeled. The description in the methods is too short. Make also clear in the results that this is a structure prediction, but not an experimentally solved structure.
7. Fig. 1c: describe in more detail how the semi-tryptic or non-tryptic peptides were obtained or filtered from the previous proteomic dataset.

Referee #3:

Summary
The authors set out to identify proteolytic processing sites in Perforin-2 (Mpeg1) based on the different orientations of cis vs trans Perforin-2 pores as well as the identification of Perforin-2 fragments in cells. Their work identified potential sites of protease cleavage within Perforin-2. They then went onto characterise these proteolytic sites, identify candidate proteases and determine the implications of protease cleavage on Perforin-2 function. One of the main outcomes from this work was the identification that the cis-pores formed on host endosome membranes does not require proteolytic processing, rather it was more a requirement for the formation of the trans-pore formed on pathogen membranes. Proteolytic sites were identified by proteomics and immunoblotting and the characterisation of proteolytic cleavage was conducted mainly in murine immortalised dendritic cells (DC), while some experiments utilised primary human DCs using various cell based assays.
While previous structural studies have shown the molecular details of pore formation for Perforin-2. This study follows on from a previous study that identified a role of Perforin-2 in the release of internalised proteins from the endosome to the cytosol for processing and presentation (Rodriguez-Silvestre 2023, Science, 380:1258). This is in addition to the proteins ability to target bacterial membranes. This is an interesting study and shows how proteolytic cleavage (or lack thereof) regulates the different roles of the protein. Pore formation is a complex and highly regulated process requiring pore forming proteins to undergo a variety of different conformational changes as it assembles and inserts in membranes. The authors have made use of the structural information available for this protein in its assembled prepore and pore states to provide more specific details at the molecular level on why Perforin-2 is not proteolytically processed when in its cis-pore conformation.
A key finding is that Perforin-2 function is regulated by proteolysis, albeit with a different implication to what has been observed for other pore formers such as the MACPFs, gasdermins and CDCLs. Whereas in the case of these other proteins proteolytic cleavage activates the protein to enable oligomerisation, the proteolytic events studied here seemed to be required for later stages of the pore formation. This makes this study more exciting in my opinion. Thus, it will be of broad interest.
Major points:
The authors identified 5 cleavage sites: of which two were evaluated more in-depth as part of the study. Have the authors considered the possibility of proteolytic cleavage at any of the other three sites that could enable the MACPF rotation required for pore formation?
An interesting observation is the ability of homologous secretases to γ-secretase to remove the TMD region. Have the authors done a deep dive into the plausibility of this by comparing Perforin-2 sequence for recognition and cleavage sites for these homologous proteases? If that information is available and included it will further validate their observation.
Given that the formation of the cis-pore on endosomes doesn't require proteolytic cleavage is a major finding in the paper. I would have liked more clarity on this in the results section. To me the sentence: "Nevertheless, saporin escape in BafA1-treated MutuDCs as well as in primary and Flt3L-derived cDC1s which maintain phagosomes at high pH, suggest that perforin-2-mediated endocytic escape is compatible with weak acidification of phagosomes." (lines 276-278) sounds contradictory. Is the

high pH in primary cells neutral or above or mildly acidic? Given that this is one of the main findings of the paper, I would suggest further clarification of this statement. Have the authors considered other environmental factors that could possibly trigger pore formation in these non-acidic/slightly acidic endocytic compartments?

No information on how the statistical analysis conducted is provided, even though the methods section mentions that this will be provided in the figure legends. e.g. Figure 3.E how did the authors estimate the significance? More information in some of the figure legends particularly in some of the supplementary figure legends will also be of value. E.g. Fig S4, what is the p28 fragment?

I would also like to suggest the inclusion of a schematic as part of the discussion highlighting key stages of pore formation in both cis and trans conformation with the inclusion of the various proteolytic cleavage events so readers would be better able to get an overview of the findings in the paper.

Minor comments:

A few more minor comments are given below:

It would be good to reference Figure 1B in the introduction where the different domains of the protein are being explained (e.g. lines 64-65) so that readers have a better idea of the domain arrangement with respect to each other.

I may have missed it but in the Methods pHrodo assay refers Section 2.5.1. Where Section 2.5.1 can be accessed is required.

Point-by-point response

Please see below our point-by-point response to reviewers' comments. Substantial changes in the manuscript are highlighted in the manuscript file. Smaller changes are also tracked for transparency.

Referee #1:

This study clarifies important issues about the biological roles and mechanisms of perforin-2 (PF2), an important effector in the immune response, both in attack of intracellular pathogens and the cross presentation of antigens. The proteolytic cleavage sites of PF2 are mapped and the proteases identified by the use of specific inhibitors. The finding that cleavage of the transmembrane anchor is not required for pore formation in the phagosome membrane along with the observations on the role of acidification lead to a plausible basis for the two different actions of PF2 - anchored monomers can assemble a cis pore and allow for antigen cross presentation, whereas the soluble ectodomains can assemble on a pathogen membrane as trans pores. Previously published mechanistic proposals were less plausible. Therefore this work provides an important advance in understanding of the actions of PF2 in different immune cell types.

The experiments have been carefully and thoroughly done, as far as this referee can discern.

We thank the reviewer for the positive feedback on the manuscript and on the model of perforin-2 action we propose.

Minor comment: The titration of saporin action is not convincing on a quantitative level, since only 2 points were measured to infer a linear (?) relationship.

We apologise for the confusion: we have performed the assay in Mpeg1^{KO} cells only at the highest concentration of saporin, but the linear relationship was inferred using data from WT cells and six saporin concentrations (0.5 mg/ml, 0.1 mg/ml, 0.02 mg/ml, 0.004 mg/ml, 0.0008 mg/ml). We now modified the legend to Figure 5D for clarity [lines 783-785].

While we have also included additional data in Figure 5D to demonstrate the reproducibility and below, we show that the average 4 % decrease in % of cells in translation arrest with a two-fold dilution of saporin on the beads was indeed observed across the different experiments.

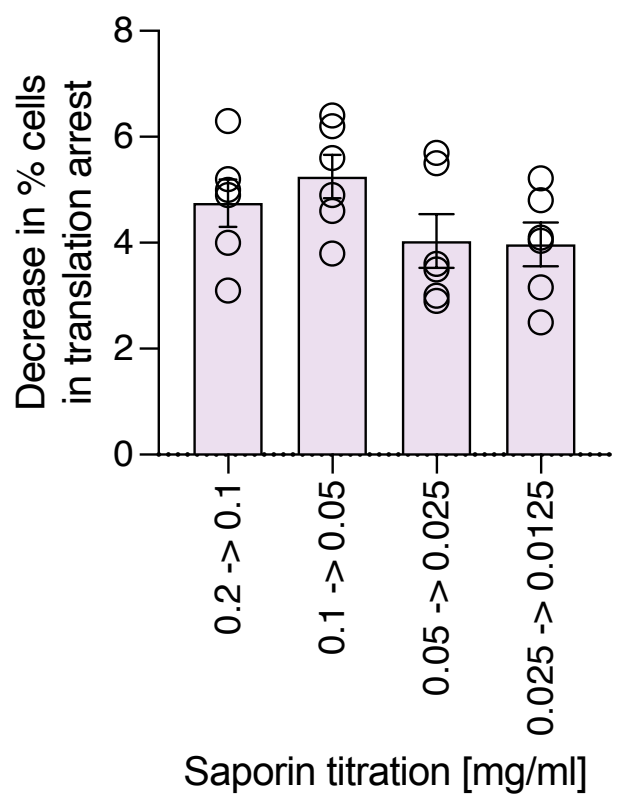

Very minor comment: although the writing is generally very clear, there are 2 or 3 sentences with missing words, eg line 257.

This has now been corrected:
*Perforin-2-mediated endocytic **escape** is not abolished by the V-ATPase inhibitor.* [line 264].

We have also carefully gone through the manuscript and corrected the text and figure legends for clarity and missing words.

Referee #2:

The protein perforin-2 has a central role in an important immunological process referred to as cross-presenting. While the authors have previously shown that the transmembrane protein perforin-2 can be proteolytically cleaved, the cleavage sites and the contributing proteases were previously largely unknown. More importantly, the functional relevance of proteolysis for the role of perforin-2 in cross-presenting remained entirely unclear and controversially discussed. Exactly these open questions are addressed and solved in the current manuscript. The authors demonstrate that perforin-2 undergoes shedding by acidic cathepsins, followed by intramembrane cleavage mediated by gamma-secretase. But the authors also demonstrate that both proteolytic events are not required for perforin's role in cross-presenting, but rather represent a degradation pathway. Based on the new findings, the authors propose a structure-function model for perforin's role in cross-presenting.

I am not an expert on cross-presenting and, thus, cannot fully judge the importance of the work for the perforin and cross-presenting field.

The study is novel and interesting and largely well controlled. The following points need to be addressed to improve the manuscript and should be easy to address.

We thank the reviewer for the positive comments on the manuscript and appreciating our work.

Major points:

1. Fig. 3C: the authors claim to have knocked-out subunits of gamma-secretase, but do not show proof. Provide a Western blot demonstrating the lack of the corresponding protein. This is easily feasible for Psen2 and Ncstn and may work for Aph1c as well. Alternatively, PCR may be used for Aph1c using mutation-specific primers.

We apologise for this omission. We have initially confirmed knockout efficiencies by PCR, but these data were not included in the original version of the manuscript. We now provide the Western blot to demonstrate deletion of Ncstn and Psen2 and in the Appendix Figure S3.

We were not able to identify a suitable antibody for Aph1c. During the revisions, we also realised that out of the three partially redundant Aph1 homologues, only Aph1a, and not Aph1b or Aph1c are detected by proteomics in MutuDCs (see below, from Kozik et al, Cell Reports, 2020). Therefore, we decided the Aph1c^{KO} data do not meaningfully contribute to the manuscript and we have decided to remove it from the updated version.

Gene names	Estimated copy number per cell	Copy number percentile
Psen1	91,612	58%
Psen2	24,748	38%
Aph1a	31,412	42%
Ncstn	168,265	70%

2. Figs. 2, 3, 4: loading controls, such as actin, which are typically used as standard quality control for immunoblots, are entirely missing. They should be added, at least for panels 4D and E, where strong effects on full-length perforin-2 are seen in the absence of CTFs.

In our hands (and especially in MutuDCs), Ponceau S stain of the membrane is a more reliable control for protein loading compared to a single housekeeping protein, as any single housekeeping protein might be affected by the experimental treatment. Using protein stains instead of housekeeping genes is also in line with published guidelines (e.g. [1], [2]). Ponceau S staining to control for protein loading is also frequently used in papers published in EMBO Journal (e.g. recently: [3]).

Therefore, we have provided the Ponceau S stained blots for all Western Blots (Fig 2B, 2C, 3B, 4C, 4D and 4E) in the original version of the manuscript, now Appendix Figure S1. Please note that we have not included these in main figures for space constraints, as wanted to use the entire lanes rather than cut out bands, to make sure all potential cleavage fragments are visualised.

[1] Fosang, A. J. & Colbran, R. J. (2015). Transparency Is the Key to Quality. *Journal of Biological Chemistry*, *290*(50), 29692–29694. https://doi.org/10.1074/jbc.e115.000002

[2] Romero-Calvo, I., Ocón, B., Martínez-Moya, P., Suárez, M. D., Zarzuelo, A., Martínez-Augustin, O. & Medina, F. S. de. (2010). Reversible Ponceau staining as a loading control alternative to actin in Western blots. *Analytical Biochemistry*, *401*(2), 318–320. https://doi.org/10.1016/j.ab.2010.02.036

[3] Lemus, L., Meyer, H., Rodríguez-Rosado, A. I., Schuldiner, M. & Goder, V. (2026). O-mannosylation of misfolded ER proteins promotes ERAD. *The EMBO Journal*, *45*(2), 564–591. https://doi.org/10.1038/s44318-025-00647-2

3. Describe in more detail how the shedding site at M599 was identified - were both peptides identified, the one ending in M599 and the one starting with amino acid 600? If not, how can the authors be sure that cleavage happens C-terminally of M599 and not further C-terminally, followed by carboxypeptidase trimming as it is frequently seen for other shedding and gamma-secretase substrates? Having a shedding site more than 50 amino acids away from the membrane is rare, as most cleavage sites are closer to the membrane. If the authors are not sure about this cleavage site, the corresponding part in the results should be phrased more carefully. Alternatively, experiments can further address this question, such as determining the N-terminus of the C-terminal fragment or testing point mutations that may abolish cleavage by cathepsins.

1) We now describe in more details how the non-tryptic peptides were identified, as well as provide all Mpeg1 peptides identified as Supplementary Table S1a. [lines 550-556]

2) The reviewer raises an important point: while we detect a non-tryptic peptide LPPYTKPPLM^, we did not detect the corresponding peptide ^SQVATNTVIVTNSETAR in this data set, raising the possibility that cleavage closer to the TMD is followed by trimming. Unfortunately, in that dataset, we did not identify any N-terminally cleaved peptides within the CTT.

Therefore, in order to verify whether M599^ is indeed where the shedding site is located, we performed immunoprecipitation experiment with the antibody binding to the C-terminal tail of perforin-2. In this new dataset, we have identified several N-terminally cleaved peptides, including ^Q^{601}VATNTVIVTNSETAR (see Figure 1 below), but in line with the possibility that the initial cleavage could take place upstream or downstream of M599.

Nevertheless, when we mapped all of these alternative sites detected by the analysis of the N-terminally cleaved peptides (see Figure 2 below). It appears that all of these residues are masked by the P2 domain, in line with the model that the initial cleavage site appears inaccessible in the pre-pore.

Surprisingly however, the C-term antibody has also pulled down several C-terminally cleaved peptides in two regions: near the I296^ cleavage in the MACPF domain and within the CTT domain. The most likely explanation is that cleavage does not happen simultaneously in all subunits of the oligomer, and the C-terminally cleaved peptides are pulled down from other subunits. It does, however, obscure the interpretation of these data, and for this reason, we decided not to include these results in the manuscript.

Instead, considering that most of the CTT is in fact masked by the P2 domain, we decided to rephrase this section as suggested by the reviewer. [line 302-204]:

> *Second, it suggests that CTT might not be accessible to the proteases as the is largely concealed by the P2 domain (including the putative M599^ shedding site) (Figure 6B).*

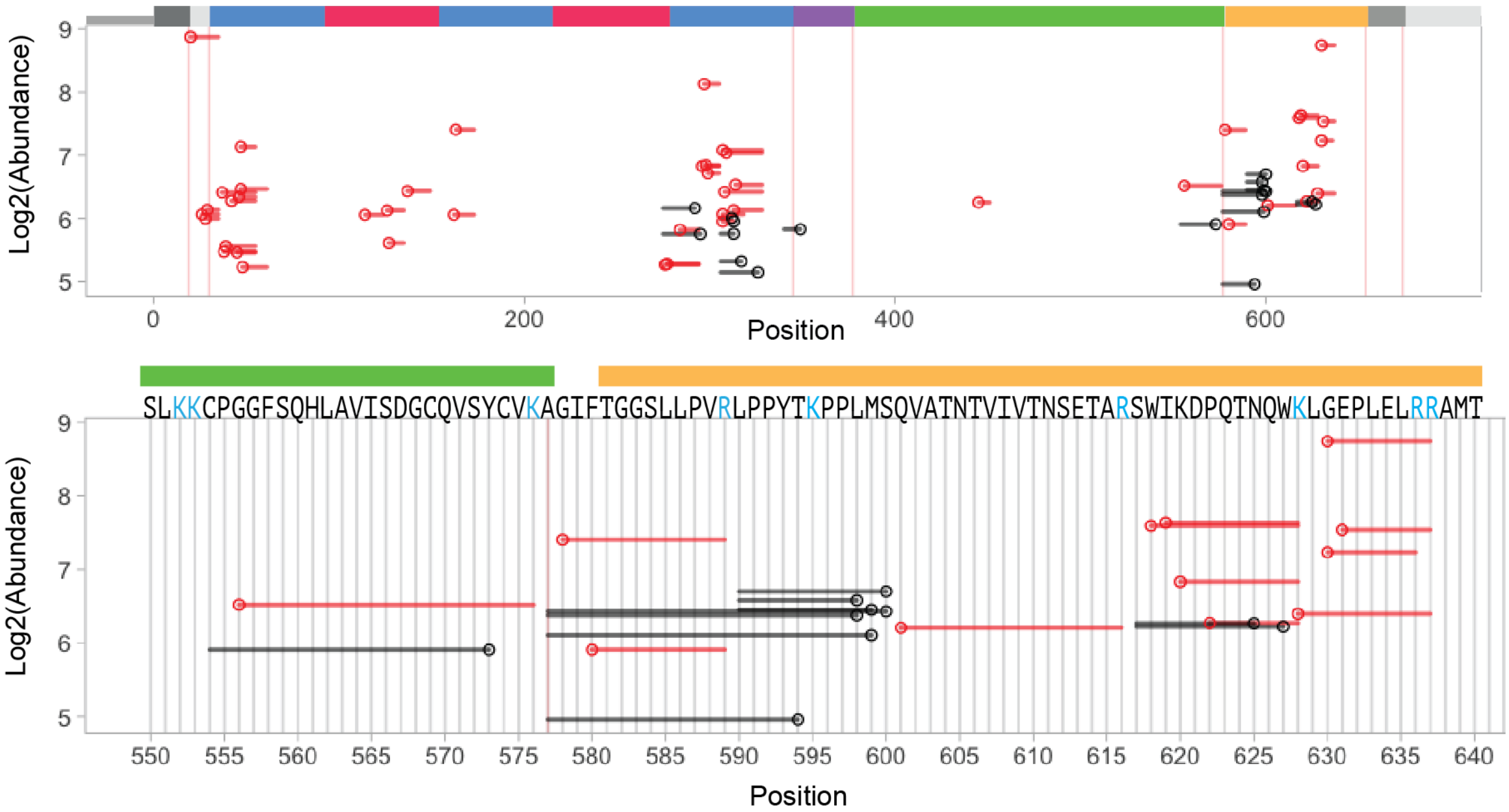

Figure 1. *MutuDCs were lysed and perforin-2 was immunoprecipitated with anti-C term antibody. Non-tryptic peptides identified by mass-spectrometry (top: all, bottom: zoomed in on residues 550-640). Non-tryptic termini indicated with a circle.*

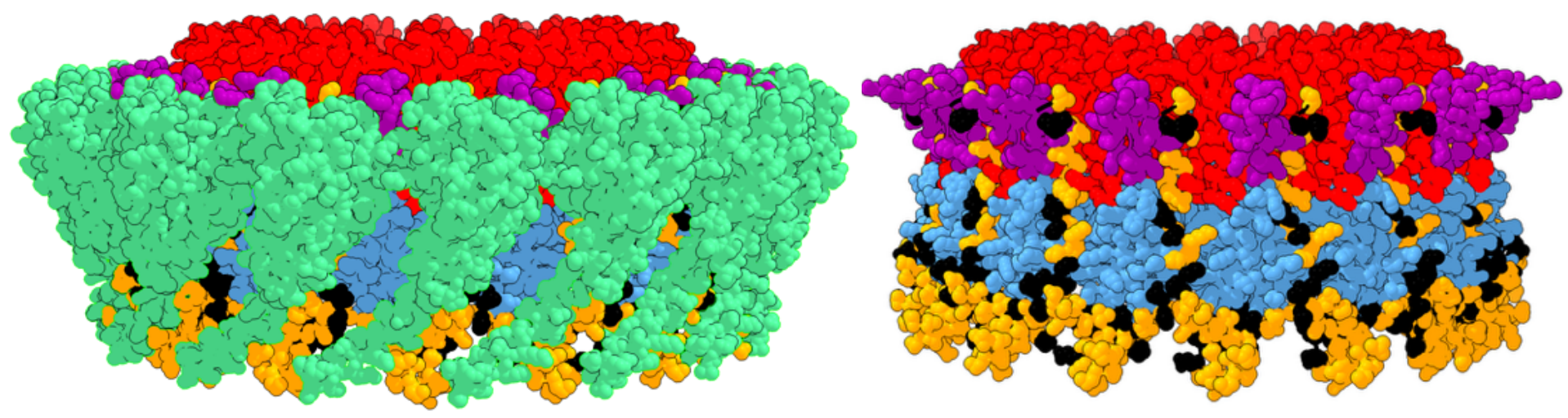

In black: 578,580,581,591,593,594,598,599,600,601,602,606,618,619,620,622,625,627,628,629,630,631

Figure 2. *Residues corresponding to termini of non-tryptic peptides detetcted by immunoprecipitation with C-term antibody (left: 6sb3, right: 6sb3 with hidden P2 domain).*

Minor points:

4. Line 179: The signal from the P2-loop antibody for NT peaks at 1 h, not at 2 h. Please correct.

Corrected.

5. Fig. 5A: describe the assay in more detail, at least in the figure legend. The assay is difficult to understand for scientists without prior knowledge of this assay.

We now describe the assay in more detail in the legend for Figure 5A [lines 770-777]. We have also added a gate in 5B to highlight the cells where saporin escaped into the cytosol causing translation arrest and inefficient puromycin incorporation.

6. Fig. 6A: describe in more detail how the structure was modeled. The description in the methods is too short.

We now explain step by step how structure in Figure 6A was modelled. [lines 568-574]

Make also clear in the results that this is a structure prediction, but not an experimentally solved structure.

We now modifed the legend for Figure 6 [lines 817-823] to say:

> *Predicted model of the pre-pore to cis pore conformational transition of full-length perforin-2 based on the experimental structures pdb:6sb3, pdb:6sb5 (Ni et al, 2020), and the AF-A1L314-F1 Alphafold model (Jumper et al, 2021).*

7. Fig. 1c: describe in more detail how the semi-tryptic or non-tryptic peptides were obtained or filtered from the previous proteomic dataset.

Peptide identification was performed in our previous study, Rodriguez-Silvestre et al, which is why we have not described it in detail in this version of the manuscript. However, following reviewer's comment, we have now expanded the relevant method section. [lines: 550-556]

In addition, we also include LFQ intensities and sequences of identified perforin-2 peptides in Table S1a.

Referee #3:

Summary

The authors set out to identify proteolytic processing sites in Perforin-2 (Mpeg1) based on the different orientations of cis vs trans Perforin-2 pores as well as the identification of Perforin-2 fragments in cells. Their work identified potential sites of protease cleavage within Perforin-2. They then went onto characterise these proteolytic sites, identify candidate proteases and determine the implications of protease cleavage on Perforin-2 function. One of the main outcomes from this work was the identification that the cis-pores formed on host endosome membranes does not require proteolytic processing, rather it was more a requirement for the formation of the trans-pore formed on pathogen membranes. Proteolytic sites were identified by proteomics and immunoblotting and the characterisation of proteolytic cleavage was conducted mainly in murine immortalised dendritic cells (DC), while some experiments utilised primary human DCs using various cell based assays.

While previous structural studies have shown the molecular details of pore formation for Perforin-2. This study follows on from a previous study that identified a role of Perforin-2 in the release of internalised proteins from the endosome to the cytosol for processing and presentation (Rodriguez-Silvestre 2023, Science, 380:1258). This is in addition to the proteins ability to target bacterial membranes. This is an interesting study and shows how proteolytic cleavage (or lack thereof) regulates the different roles of the protein. Pore formation is a complex and highly regulated process requiring pore forming proteins to undergo a variety of different conformational changes as it assembles and inserts in membranes. The authors have made use of the structural information available for this protein in its assembled prepore and pore states to provide more specific details at the molecular level on why Perforin-2 is not proteolytically processed when in its cis-pore conformation.

A key finding is that Perforin-2 function is regulated by proteolysis, albeit with a different implication to what has been observed for other pore formers such as the MACPFs, gasdermins and CDCLs. Whereas in the case of these other proteins proteolytic cleavage activates the protein to enable oligomerisation, the proteolytic events studied here seemed to be required for later stages of the pore formation. This makes this study more exciting in my opinion. Thus, it will be of broad interest.

We would like to thank the reviewer for the positive comments on the manuscript.

Major points:

The authors identified 5 cleavage sites: of which two were evaluated more in-depth as part of the study. Have the authors considered the possibility of proteolytic cleavage at any of the other three sites that could enable the MACPF rotation required for pore formation?

The three cleavage sites that we did not characterise in this work are: (1) a site at position L38^, which would remove a short N-terminal fragment; and (2-3) two sites within the MACPF domain (D210^ and I296^).

We have excluded the cleavages in the MACPF domain (2,3) as a plausible trigger for conformational change, as these are on the two edges of the THM2 (see the diagram below) and if the cleavages occurred prior to conformational change they would disrupt the pore forming domain.

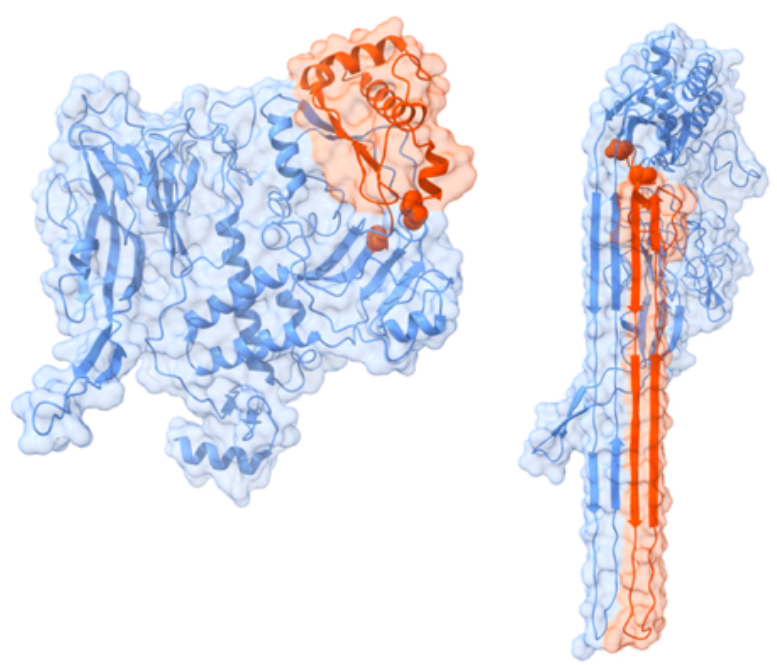

Residues preceeding cleavage sites (D210, I296) in sphere style
D210-I296 in red

Left: pdb 6sb3 (pre-pore); right: pdb 6sb5 (pore)

We are currently exploring in the lab the function of the N-terminal fragment. Our two working models are that the cleavage of the N-terminal fragment: 1) influences selectivity of the pores as it is located at the luminal side of the pore opening; or 2) it indeed inititiates pore formation, as reported for some bacterial and insecticidal MACPF pore-forming proteins [1,2]. To date, however, we have not succeeded to identify the protease responsible for the N-term cleavage or a strategy to inhibit it. Thus, while the role of N-term fragment is possibly important, we believe it goes beyond the key question of this manuscript, which is to define the role of shedding and TMD in pore formation by perforin-2. Nevertheless, we have expanded the discussion section to comment on the possible role of this cleavage event in pore formation. [lines: 371-375]

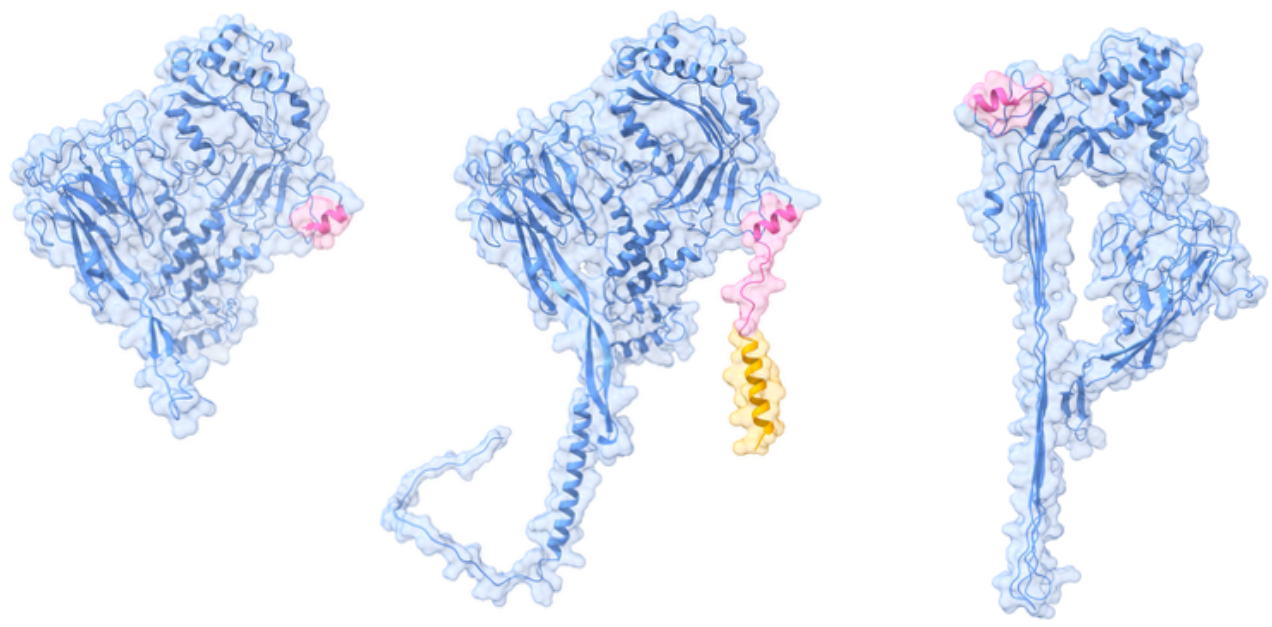

Signal papetide
*N-term fragment removed by cleavage at L38^*

Left: pdb 6sb3 (pre-pore); middle (Alphafold2 model); right: pdb 6sb5 (pore)

[1] Marini, G. et al. Structural journey of an insecticidal protein against western corn rootworm. Nat. Commun. 14, 4171 (2023).

[2] Abrahamsen, H. L. et al. Distant relatives of a eukaryotic cell-specific toxin family evolved a complement-like mechanism to kill bacteria. Nat. Commun. 15, 5028 (2024).

An interesting observation is the ability of homologous secretases to γ-secretase to remove the TMD region. Have the authors done a deep dive into the plausibility of this by comparing Perforin-2 sequence for recognition and cleavage sites for these homologous proteases? If that information is available and included it will further validate their observation.

In the original version of the manuscript, we have searched for any known cleavage sites/motifs using the ELM database (see Table S1b), but this search did not reveal any consensus sequences within the TMD of perforin-2. However, prompted by the reviewer's comment, we have now more carefully reviewed the cleavage requirements for the γ-secretase. Analysis of the published data suggests that the major determinant of γ-secretase cleavage is the length of the luminal domain following shedding [1].

However, while for the majority of the reported ectodomains after shedding are slightly under 15 aa (thereby frequently requiring shedding prior to processing by γ-secretase), there does not appear to be a universal cut-off, with several substrates with longer ectodomains identified. The proposed site of shedding in perforin-2 is at position 599, which would result in ectodomain of 54 aa. This , but in line with ectodomain lengths observed for other γ-secretase substrates, such as the B-cell maturation antigen [2].

Intriguingly, we have also observed that perforin-2, similar to one of the canonical substrates of γ-secretase, APP, has several GxxxG motifs within the juxtamembrane and transmembrane region (see below). However, while the GxxxG motif has been shown to affect γ-secretase processivity during APP cleavage [3], it is not required for cleavage of other substrates and it is unclear whether it plays a role in perforin-2 processing.

Thus, in the revised version of the manuscript, we now clarify that γ-secretase preferentially acts of substrates with short luminal domains [lines 195-196], but due to the lack of the clear cleavage determinants across the different substrates, we have not discussed this in detail to avoid detracting from the main focus of the paragraph.

GSNKGAIIGLMVGGVVIATVIVITLVMLKKK	APP:
GDSNGMSGGEAAGITLGVTIALGIVITLAIYGTRK	Perforin-2

TMD in red (*based on UniProt annotations)*

[1] Funamoto, S. et al. Substrate ectodomain is critical for substrate preference and inhibition of γ-secretase. Nat. Commun. 4, 2529 (2013).

[2] Laurent, S. A. et al. γ-secretase directly sheds the survival receptor BCMA from plasma cells. Nat. Commun. 6, 7333 (2015).

[3] Higashide, H., Ishihara, S., Nobuhara, M., Ihara, Y. & Funamoto, S. Alanine substitutions in the GXXXG motif alter C99 cleavage by γ-secretase but not its dimerization. J. Neurochem. 140, 955–962 (2017).

Given that the formation of the cis-pore on endosomes doesn't require proteolytic cleavage is a major finding in the paper. I would have liked more clarity on this in the results section. To me the sentence: "Nevertheless, saporin escape in BafA1-treated MutuDCs as well as in primary and Flt3L-derived cDC1s which maintain phagosomes at high pH, suggest that perforin-2-mediated endocytic escape is compatible with weak acidification of phagosomes." (lines 276-278) sounds contradictory. Is the high pH in primary cells neutral or above or mildly acidic? Given that this is one of the main findings of the paper, I would suggest further clarification of this statement.

We would like to thank the reviewer for drawing our attention to the fact, that the relative descriptions of phagosomal pH have been unclear throughout the paper. We now rephrased “high pH” with near-neutral pH throughout the manuscript.

We have also rephrased the sentence highlighted by the reviewer for the following:

> *Nevertheless, saporin escape in BafA1-treated MutuDCs as well as in primary and Flt3L-derived cDC1s (Rodríguez-Silvestre et al, 2023), which maintain phagosomes at near-neutral pH, suggests that perforin-2-mediated endocytic escape does not require strongly acidic environment.*

Have the authors considered other environmental factors that could possibly trigger pore formation in these non-acidic/slightly acidic endocytic compartments?

Indeed, it is likely that pore formation in the near-neutral compartments of cDC1s is not triggered by pH change. We have recently performed a genome-wide screen for regulators of endocytic escape which revealed as hits a range of enzymes involved in protein trafficking, post-translational modifications, as well as other putative co-factors. These include proteins involved in glycosylation, sulfation, as well as in lipid metabolism. These findings are in line with the hypothesis that other factors could control pore formation in cis. We are also exploring the hypothesis that cleavage of the N-terminal helix might act as trigger (see also response to Reviewer 2, Question 1), but at this point, we do not have tools to inhibit

it. While we believe this important question is beyond the scope of this paper, we have now expanded the discussion section to explicitly consider other mechanisms. [lines 558-592]

No information on how the statistical analysis conducted is provided, even though the methods section mentions that this will be provided in the figure legends. e.g. Figure 3.E how did the authors estimate the significance?

We thank the reviewer for pointing out that that a couple of panels were missing information on the statistical analysis. We have now added this in legends to Figures 2F, 3E, 4G, 5E and 6D.

More information in some of the figure legends particularly in some of the supplementary figure legends will also be of value. E.g. Fig S4, what is the p28 fragment?

We have now added a diagram in Appendix Figure S5B (previously Figure S4) with the perforin-2 fragments relevant for the Western Blot in C. We have mistakenly labelled the p30 fragment as p28 which has now been corrected. We have also edited figure legends for consistency with EMBO Journal guidelines and added additional information to improve clarity (e.g. see Figure 5 and 6).

I would also like to suggest the inclusion of a schematic as part of the discussion highlighting key stages of pore formation in both cis and trans conformation with the inclusion of the various proteolytic cleavage events so readers would be better able to get an overview of the findings in the paper.

We thank reviewer for this suggestion. We now include a diagram (a new Figure 7) highlighting the key cleavages and our model of the spatio-temporal regulation of these.

Minor comments:

A few more minor comments are given below:

It would be good to reference Figure 1B in the introduction where the different domains of the protein are being explained (e.g. lines 64-65) so that readers have a better idea of the domain arrangement with respect to each other.

Reference to Figure 1A,B has been added in the introduction as suggested. [line 85]

I may have missed it but in the Methods pHrodo assay refers Section 2.5.1. Where Section 2.5.1 can be accessed is required.

We have now removed the incorrect reference to Section 2.5.1 and the new version of the methods [lines 764-766] states:

> *For pHrodo assays with MutuDCs and BMDCs, the cells were pulsed with beads as described in the phagoFACS assay protocol, but using pHrodo-labelled beads (see above for bead preparation protocol).*

Dr. Patrycja Kozik
MRC Laboratory of Molecular Biology
Protein and Nucleic Acid Chemistry
Francis Crick Avenue
Cambridge
United Kingdom

9th Jun 2026

Re: EMBOJ-2025-123194R
Anchoring of perforin-2 via the transmembrane domain is required for antigen escape into the cytosol

Dear Patrycja,

Thank you for submitting your revised manuscript on perforin-2 pore formation for our consideration. It has now been re-reviewed by two of the original referees (comments copied below), who were fully satisfied with the revisions and only note a few remaining presentational issues that I would ask you to incorporate during a final round of minor revision. In addition, there are a few editorial issues that should be taken care of at this stage:

- Importantly, our routine pre-acceptance image checks indicated that several FACS panels from Figure 3D also appear in Figure 4F - please check. If intentional, this would need to be clearly stated in both respective figure legends and clearly rationalized in the text. The same applies to FACS panels in Appendix Figure S4A and S4C, and in Appendix Figure S6B and S6C. For Appendix Figure S5C, the legend should probably indicated that all three antibody probings have been done on the same membrane?

- Please adjust the order of the manuscript sections, and also make sure to use the correct section headers:
Single title page with complete (full-name!) author information, Abstract, Introduction, Results, Discussion, Methods, Data Availability, Acknowledgements, Disclosure and Competing Interests Statement, References, Main Figure Legends, Tables, Expanded Figure Legends.

- As we are switching from a free-text author contribution statement towards a more formal statement based on Contributor Role Taxonomy (CRediT) terms, please remove the present Author Contribution section and instead specify each author's contribution(s) directly in the Author Information page of our submission system during upload of the final manuscript. See https://casrai.org/credit/ for more information.

- Please carefully go through the reference list and make sure that all references have their complete citation information - including year, journal name & volume, and page/elocator numbers - such information is currently missing for several of them.

- Please check that all figure panels are referenced from the text in a sequential manner (by Figure number/panel label - e.g. 1A before 1B) - if necessary, you may first call-out the complete figure (e.g., Fig 3A-E) before referencing individual panels.

- Please provide the exact p values in the legends of 2F; 3E; EV-2B.

- Please upload the Appendix file in PDF format without any (revision) mark-up, and include a title page stating "Appendix for [ms title]" and containing a Table of Content listing the included figures with respective page numbers. Make sure to adjust the nomenclature of the included figures to "Appendix Figure S1-7" on the figures, in their legends and in the ToC; as well as when referring to them throughout the main manuscript.

- 'Table S1' should be renamed/converted into Expanded View 'Dataset EV1', both in the file and when referring to it from the main manuscript. Please include in the XLSX file a separate 'Legends' tab, also mentioning this title and including a minimum amount of legend information for the included contents.

- Finally, please provide suggestions for a short 'blurb' text prefacing and summing up the conceptual aspect of the study in two sentences (max. 250 characters), followed by 3-5 one-sentence 'bullet points' with brief factual statements of key results of the paper; they will form the basis of an editor-written 'Synopsis' accompanying the online version of the article. Please also upload a synopsis image, which can be used as a "visual title" for the synopsis section of your paper. The image (maybe based on a compacted version of Figure 7?) should be ideally in JPG format, and please make sure that it remains in the modest dimensions of (exactly) 550 pixels wide and between 300 pixels high.

I am returning the manuscript to you for a final round of minor revision, solely to allow you to make these modifications and upload the revised files. Once we will have received them, we should be ready to swiftly proceed with formal acceptance and

production of the manuscript.

With kind regards,

Hartmut

Hartmut Vodermaier, PhD
Senior Editor, The EMBO Journal
h.vodermaier@embojournal.org

*** PLEASE NOTE: All revised manuscript are subject to initial checks for completeness and adherence to our formatting guidelines. Revisions may be returned to the authors and delayed in their editorial re-evaluation if they fail to comply to the following requirements. As a first step please read our guidelines for revised submissions:
https://link.springer.com/journal/44318/submission-guidelines#cms-Revised-submissions

1) Every manuscript requires a Data Availability section (even if only stating that no deposited datasets are included). Primary datasets or computer code produced in the current study have to be deposited in appropriate public repositories prior to resubmission, and reviewer access details provided in case that public access is not yet allowed.

2) Each figure legend must specify
- size of the scale bars that are mandatory for all micrograph panels
- the statistical test used to generate error bars and P-values
- the type error bars (e.g., S.E.M., S.D.)
- the number (n) and nature (biological or technical replicate) of independent experiments underlying each data point
- Figures may not include error bars for experiments with n<3; scatter plots showing individual data points should be used instead.

3) Revised manuscript text (including main tables, and figure legends for main and EV figures) has to be submitted as editable text file (e.g., .docx format). We encourage highlighting of changes (e.g., via text color) for the referees' reference.

4) Each main and each Expanded View (EV) figure should be uploaded as individual production-quality files (preferably in .eps, .tif, .jpg formats). For suggestions on figure preparation/layout, please refer to our Figure Preparation Guidelines:
https://media.springernature.com/original/springer-cms/rest/v1/content/27825798/data/v1

5) Point-by-point response letters should include the original referee comments in full together with your detailed responses to them (and to specific editor requests if applicable), and also be uploaded as editable (e.g., .docx) text files.

6) Please complete our Author Checklist, and make sure that information entered into the checklist is also reflected in the manuscript; the checklist will be available to readers as part of the Review Process File.

7) All authors listed as (co-)corresponding need to deposit, in their respective author profiles in our submission system, a unique ORCiD identifier linked to their name. Please see our Guide to Authors for detailed instructions.

8) Please note that supplementary information at EMBO Press has been superseded by the 'Expanded View' for inclusion of additional figures, tables, movies or datasets; with up to five EV Figures being typeset and directly accessible in the HTML version of the article.

9) To facilitate reproducibilty and cross-laboratory adoption of methodologies, please structure the Materials & Methods section as outlined in our guide to authors, including a completed Reagents and Tools Table.

10) Digital image enhancement is acceptable practice, as long as it accurately represents the original data and conforms to community standards. If a figure has been subjected to significant electronic manipulation, this must be clearly noted in the figure legend and/or the 'Materials and Methods' section. The editors reserve the right to request original versions of figures and the original images that were used to assemble the figure. Finally, we generally encourage uploading of numerical as well as gel/blot image source data.

At EMBO Press we ask authors to provide source data for the main manuscript figures. You will receive a separate email with instructions for providing source data with your revised manuscript, including how to upload and organize the files.

In the interest of ensuring the conceptual advance provided by the work, we recommend submitting a revision within 3 months (7th Sep 2026). Please discuss the revision progress ahead of this time with the editor if you require more time to complete the

revisions. Use the link below to submit your revision:

Link Not Available

Referee #2:

The authors have adequately addressed my previous concerns.
There is only one minor point to be corrected, as the two words "cleavage site" are missing in the sentence in lines 302-304. The current sentence "Second, it suggests that CTT might not be accessible to the proteases as the is largely concealed by the P2 domain (including the putative M599^ shedding site) (Figure 6B)" does not make sense without these words.

Referee #3:

The original version of the paper reviewed the proteolytic processing of Perforin-2 (Mpeg1) to identify potential proteolytic processing and discussed its implications on the role of Perforin-2 on host cell membranes.
My main comments on the first draft of the paper were to do with including more information on some of the cleavage sites that were not described in detail, further clarity around one of the aims of the paper i.e. the pH requirements for cis-pore formation, and the lack of details on statistical analysis for the results presented. Other than that, I also suggested a schematic summarising the authors findings and a few other details that I thought were missing and that could add to the clarity of the paper. Having read through the authors responses, I believe the authors have more than adequately addressed these queries.

A few minor edits are suggested:
- Lines 558-592 do not correspond. I think the authors mean lines 371-375. If so this addresses the suggest comment,

- Figures 5E, 6D perhaps worth clarifying if these are representative graphs of how many independent experiments, i.e. "representative of x number of independent experiments", As has been done in Figure 4G.

- Line 764-766 is in fact 521-523 but addresses the point brought up by this referee as a comment on the first version of the paper.

Referee #2:

The authors have adequately addressed my previous concerns. There is only one minor point to be corrected, as the two words "cleavage site" are missing in the sentence in lines 302-304. The current sentence "Second, it suggests that CTT might not be accessible to the proteases as the is largely concealed by the P2 domain (including the putative M599^ shedding site) (Figure 6B)" does not make sense without these words.

The sentences has been corrected and now reads:

Second, it suggests that most of the CTT (including the putative M599^ shedding site) might not be accessible to proteases, because it is largely concealed by the P2 domain (Figure 6B).

Referee #3:

The original version of the paper reviewed the proteolytic processing of Perforin-2 (Mpeg1) to identify potential proteolytic processing and discussed its implications on the role of Perforin-2 on host cell membranes.

My main comments on the first draft of the paper were to do with including more information on some of the cleavage sites that were not described in detail, further clarity around one of the aims of the paper i.e. the pH requirements for cis-pore formation, and the lack of details on statistical analysis for the results presented. Other than that, I also suggested a schematic summarising the authors findings and a few other details that I thought were missing and that could add to the clarity of the paper. Having read through the authors responses, I believe the authors have more than adequately addressed these queries.

A few minor edits are suggested:

- Lines 558-592 do not correspond. I think the authors mean lines 371-375. If so this addresses the suggest comment,

We apologize for the incorrect line reference. The reviewer is correct.

- Figures 5E, 6D perhaps worth clarifying if these are representative graphs of how many independent experiments, i.e. "representative of x number of independent experiments", As has been done in Figure 4G.

We now show individual data points from independents experiments and state this in the legend (including number of experiments in each case).

Line 764-766 is in fact 521-523 but addresses the point brought up by this referee as a comment on the first version of the paper.

We apologize for the incorrect line reference. The reviewer is correct.

EMBO Press Author Checklist

Corresponding Author Name: Patrycja Kozik	
Journal Submitted to: The EMBO Journal	
Manuscript Number: EMBOJ-2025-123194R	

USEFUL LINKS FOR COMPLETING THIS FORM
The EMBO Journal - Author Guidelines
EMBO Reports - Author Guidelines
Molecular Systems Biology - Author Guidelines
EMBO Molecular Medicine - Author Guidelines

Reporting Checklist for Life Science Articles (updated January
This checklist is adapted from Materials Design Analysis Reporting (MDAR) Checklist for Authors. MDAR establishes a minimum set of requirements in transparent reporting in the life sciences (see Statement of Task: 10.31222/osf.io/9sm4x). Please follow the journal's guidelines in preparing your
Please note that a copy of this checklist will be published alongside your article.

Abridged guidelines for figures
1. Data
The data shown in figures should satisfy the following conditions:
- the data were obtained and processed according to the field's best practice and are presented to reflect the results of the experiments in an accurate and unbiased manner.
- ideally, figure panels should include only measurements that are directly comparable to each other and obtained with the same assay.
- plots include clearly labeled error bars for independent experiments and sample sizes. Unless justified, error bars should not be shown for technical
- if n<5, the individual data points from each experiment should be plotted. Any statistical test employed should be justified.
- Source Data should be included to report the data underlying figures according to the guidelines set out in the authorship guidelines on Data

2. Captions
Each figure caption should contain the following information, for each panel where they are relevant:
- a specification of the experimental system investigated (eg cell line, species name).
- the assay(s) and method(s) used to carry out the reported observations and measurements.
- an explicit mention of the biological and chemical entity(ies) that are being measured.
- an explicit mention of the biological and chemical entity(ies) that are altered/varied/perturbed in a controlled manner.
- the exact sample size (n) for each experimental group/condition, given as a number, not a range;
- a description of the sample collection allowing the reader to understand whether the samples represent technical or biological replicates (including how many animals, litters, cultures, etc.).
- a statement of how many times the experiment shown was independently replicated in the laboratory.
- definitions of statistical methods and measures:
  - common tests, such as t-test (please specify whether paired vs. unpaired), simple χ2 tests, Wilcoxon and Mann-Whitney tests, can be unambiguously identified by name only, but more complex techniques should be described in the methods section;
  - are tests one-sided or two-sided?
  - are there adjustments for multiple comparisons?
  - exact statistical test results, e.g., P values = x but not P values < x;
  - definition of 'center values' as median or average;
  - definition of error bars as s.d. or s.e.m.

Please complete ALL of the questions below.
Select "Not Applicable" only when the requested information is not relevant for your study.

Materials

Newly Created Materials	Information included in the manuscript?	In which section is the information available? (Reagents and Tools Table, Materials and Methods, Figures, Data Availability Section)
New materials and reagents need to be available; do any restrictions apply?	Yes	Reagents and Tools Table

Antibodies	Information included in the manuscript?	In which section is the information available? (Reagents and Tools Table, Materials and Methods, Figures, Data Availability Section)
For **antibodies** provide the following information:<br>- Commercial antibodies: RRID (if possible) or supplier name, catalogue number and or/clone number<br>- Non-commercial: RRID or citation	Yes	Reagents and Tools Table

DNA and RNA sequences	Information included in the manuscript?	In which section is the information available? (Reagents and Tools Table, Materials and Methods, Figures, Data Availability Section)
**Short novel DNA or RNA including primers, probes**: provide the sequences.	Yes	Materials and Methods, EV Tables

Cell materials	Information included in the manuscript?	In which section is the information available? (Reagents and Tools Table, Materials and Methods, Figures, Data Availability Section)
**Cell lines:** Provide species information, strain. Provide accession number in repository **OR** supplier name, catalog number, clone number, and/**OR** RRID.	Yes	Reagents and Toools, Materials and Methods
**Primary cultures:** Provide species, strain, sex of origin, genetic modification status.	Yes	Materials and Methods, Reagents and Tools
Report if the cell lines were recently **authenticated** (e.g., by STR profiling) and tested for mycoplasma contamination.	Yes	Materials and Methods

Experimental animals	Information included in the manuscript?	In which section is the information available? (Reagents and Tools Table, Materials and Methods, Figures, Data Availability Section)
**Laboratory animals or Model organisms:** Provide species, strain, sex, age, genetic modification status. Provide accession number in repository **OR** supplier name, catalog number, clone number, **OR** RRID.	Yes	Materials and Methods, Reagents and Tools Table
**Animal observed in or captured from the field:** Provide species, sex, and age where possible.	Not Applicable	
Please detail **housing and husbandry conditions**.	Yes	Materials and Methods

Plants and microbes	Information included in the manuscript?	In which section is the information available? (Reagents and Tools Table, Materials and Methods, Figures, Data Availability Section)
**Plants:** provide species and strain, ecotype and cultivar where relevant, unique accession number if available, and source (including location for collected wild specimens).	Not Applicable	
**Microbes:** provide species and strain, unique accession number if available, and source.	Not Applicable	

Human research participants	Information included in the manuscript?	In which section is the information available? (Reagents and Tools Table, Materials and Methods, Figures, Data Availability Section)
If collected and within the bounds of privacy constraints report on age, sex and gender or ethnicity for all study participants.	Not Applicable	

Core facilities	Information included in the manuscript?	In which section is the information available? (Reagents and Tools Table, Materials and Methods, Figures, Data Availability Section)
If your work benefited from core facilities, was their service mentioned in the acknowledgments section?	Yes	Acknowledgements

Design

Study protocol	Information included in the manuscript?	In which section is the information available? (Reagents and Tools Table, Materials and Methods, Figures, Data Availability Section)
If study protocol has been **pre-registered, provide DOI in the manuscript**. For clinical trials, provide the trial registration number **OR** cite DOI.	Not Applicable	
Report the **clinical trial registration number** (at ClinicalTrials.gov or equivalent), where applicable.	Not Applicable	

Laboratory protocol	Information included in the manuscript?	In which section is the information available? (Reagents and Tools Table, Materials and Methods, Figures, Data Availability Section)
Provide DOI OR other citation details if **external detailed step-by-step protocols** are available.	Not Applicable	

Experimental study design and statistics	Information included in the manuscript?	In which section is the information available? (Reagents and Tools Table, Materials and Methods, Figures, Data Availability Section)
Include a statement about **sample size** estimate even if no statistical methods were used.	Yes	
Were any steps taken to minimize the effects of subjective bias when allocating animals/samples to treatment (e.g. **randomization procedure**)? If yes, have they been described?	Not Applicable	
Include a statement about **blinding** even if no blinding was done.	Not Applicable	
Describe **inclusion/exclusion criteria** if samples or animals were excluded from the analysis. Were the criteria pre-established?<br><br>If sample or data points were omitted from analysis, report if this was due to attrition or intentional exclusion and provide justification.	Not Applicable	
For every figure, are **statistical tests** justified as appropriate? Do the data meet the assumptions of the tests (e.g., normal distribution)? Describe any methods used to assess it. Is there an estimate of variation within each group of data? **Is** the variance similar between the groups that are being statistically compared?	Yes	Figure Legends

Sample definition and in-laboratory replication	Information included in the manuscript?	In which section is the information available? (Reagents and Tools Table, Materials and Methods, Figures, Data Availability Section)
In the figure legends: state number of times the experiment was **replicated** in laboratory.	Yes	Figure Legends
In the figure legends: define whether data describe **technical or biological replicates**.	Yes	Figure Legends

Ethics

Ethics	Information included in the manuscript?	In which section is the information available? (Reagents and Tools Table, Materials and Methods, Figures, Data Availability Section)
Studies involving **human participants**: State details of **authority granting ethics approval** (IRB or equivalent committee(s), provide reference number for approval.	Not Applicable	
Studies involving **human participants**: Include a statement confirming that **informed consent** was obtained from all subjects and that the experiments conformed to the principles set out in the WMA Declaration of Helsinki and the Department of Health and Human Services Belmont Report.	Not Applicable	
Studies involving **human participants:** For publication of **patient photos**, include a statement confirming that consent to publish was obtained.	Not Applicable	
Studies involving experimental **animals**: State details of **authority granting ethics approval** (IRB or equivalent committee(s), provide reference number for approval. Include a statement of compliance with ethical regulations.	Yes	Materials and Methods
Studies involving **specimen and field samples:** State if relevant **permits** obtained, provide details of authority approving study; if none were required, explain why.	Not Applicable	

Dual Use Research of Concern (DURC)	Information included in the manuscript?	In which section is the information available? (Reagents and Tools Table, Materials and Methods, Figures, Data Availability Section)
Could your study fall under dual use research restrictions? Please check biosecurity documents and list of **select agents and toxins** (CDC): https://www.selectagents.gov/sat/list.htm	Not Applicable	
If you used a select agent, is the security level of the lab appropriate and reported in the manuscript?	Not Applicable	
If a study is subject to dual use research of concern regulations, is the name of the **authority granting approval and reference number** for the regulatory approval provided in the manuscript?	Not Applicable	

Reporting

The MDAR framework recommends adoption of discipline-specific guidelines, established and endorsed through community initiatives. Journals have their own policy about requiring specific guidelines and recommendations to complement MDAR.

Adherence to community standards	Information included in the manuscript?	In which section is the information available? (Reagents and Tools Table, Materials and Methods, Figures, Data Availability Section)
State if relevant guidelines or checklists (e.g., **ICMJE, MIBBI, ARRIVE, PRISMA**) have been followed or provided.	Not Applicable	
For **tumor marker prognostic studies,** we recommend that you follow the **REMARK** reporting guidelines (see link list at top right). See author guidelines, under 'Reporting Guidelines'. Please confirm you have followed these guidelines.	Not Applicable	
For **phase II and III randomized controlled trials**, please refer to the **CONSORT** flow diagram (see link list at top right) and submit the CONSORT checklist (see link list at top right) with your submission. See author guidelines, under 'Reporting Guidelines'. Please confirm you have submitted this list.	Not Applicable	

Data Availability

Data availability	Information included in the manuscript?	In which section is the information available? (Reagents and Tools Table, Materials and Methods, Figures, Data Availability Section)
Have **primary datasets** been deposited according to the journal's guidelines (see 'Data Deposition' section) and the respective accession numbers provided in the Data Availability Section?	Not Applicable	
Were **human clinical and genomic datasets** deposited in a public access-controlled repository in accordance to ethical obligations to the patients and to the applicable consent agreement?	Not Applicable	
Are **computational models** that are central and integral to a study available without restrictions in a machine-readable form? Were the relevant accession numbers or links provided?	Not Applicable	
If publicly available data were reused, provide the respective **data citations in the reference list**.	Yes	Materials and Methods (with References)
