# Supplementary material for: Anchoring of perforin-2 via the transmembrane domain is required for antigen escape into the cytosol
Source: EMBO J. 2026 Aug 19;45(19):6759–81. doi: 10.1038/s44318-026-00903-z (PMC13631362; doi:10.1038/s44318-026-00903-z)
Supplement: Supplementary file 4 [file 44318_2026_903_MOESM2_ESM.docx]

**Table EV1: sgRNA for KO generation**

| **Target** | **Forward oligo (5’-3’)** | **Reverse oligo (5’-3’)** |
| --- | --- | --- |
| Psen2 | CACCGATGGCACCCAAGATGACCC | AAACGGGTCATCTTGGGTGCCATC |
| Ncstn | CACCGCCAGCTGAGTGACAAAGG | AAACCCTTTGTCACTCAGCTGGC |
| Non-targeting (NT) | CACCACCGGTCGAAGTCTGGGATT | AAACAATCCCAGACTTCGACCGGT |
