# Supplementary material for: Anchoring of perforin-2 via the transmembrane domain is required for antigen escape into the cytosol
Source: EMBO J. 2026 Aug 19;45(19):6759–81. doi: 10.1038/s44318-026-00903-z (PMC13631362; doi:10.1038/s44318-026-00903-z)
Supplement: Supplementary file 5 [file 44318_2026_903_MOESM3_ESM.docx]

**Table EV2: Sequences of primers for knockout validation**

| **Target** | **Forward primer (5’-3’)** | **Reverse primer (5’-3’)** |
| --- | --- | --- |
| Ncstn | tgtgggactagaacctgacg | aagcgaaacagagagaccca |
| Psen2 | aacctagactgggacgtgtg | ggtaaacgctctgcttcctg |
