# Supplementary material for: Anchoring of perforin-2 via the transmembrane domain is required for antigen escape into the cytosol
Source: EMBO J. 2026 Aug 19;45(19):6759–81. doi: 10.1038/s44318-026-00903-z (PMC13631362; doi:10.1038/s44318-026-00903-z)
Supplement: Supplementary file 6 [file 44318_2026_903_MOESM4_ESM.docx]

**Table EV3: GeneBlock sequences for CTT mutants**

| **Mutant ID** | **GeneBlock Sequence** |
| --- | --- |
| V609R/T610R | CCCTATGACAAATGCACAGTCATGCCCTGCAGGCTACATCCCACTGAACCTGTTTGAAAGCCTCAAGGTATGTGTGTCCCTGGATTATGAGTTGGGGTTCAAGTTTTCAGTCCCCTTTGGTGGGTTCTTCAGTTGTATAATGGGGAACCCCTTGGTTAATTCTGATACAGCTAAAGACGTCAGAGCACCATCTCTGAAAAAGTGTCCCGGGGGCTTCAGCCAACACCTAGCTGTTATCAGTGATGGATGCCAAGTGTCCTACTGTGTCAAGGCTGGAATCTTCACAGGAGGGTCCCTGCTCCCTGTCAGGCTCCCACCTTATACCAAACCACCTCTTATGAGCCAGGTTGCCACCAACACTGTCATAcgccgtAATAGTGAGACTGCCAGATCCTGGATTAAGGACCCTCAGACCAACCAGTGGAAGCTGGGAGAACCTCTGGAGCTACGTAGGGCCATGACAGTCATCCATGGGGACAGTAATGGAATGTCAGGAGGGGAAGCTGCTGGAATCACTTTGGGAGTCACCATAGCACTAGGAGTTGTCATTACCTTGGCCATCTATGGTACTCGGAAGTACAAGAAGAAGGAATACCAGGAAATTGAGGAGCAGGAGAGTTTGGTTGGAAGCTTAGCAACAGATGCAACAGTCCTTAATGG |
| V609D/T610E | CCCTATGACAAATGCACAGTCATGCCCTGCAGGCTACATCCCACTGAACCTGTTTGAAAGCCTCAAGGTATGTGTGTCCCTGGATTATGAGTTGGGGTTCAAGTTTTCAGTCCCCTTTGGTGGGTTCTTCAGTTGTATAATGGGGAACCCCTTGGTTAATTCTGATACAGCTAAAGACGTCAGAGCACCATCTCTGAAAAAGTGTCCCGGGGGCTTCAGCCAACACCTAGCTGTTATCAGTGATGGATGCCAAGTGTCCTACTGTGTCAAGGCTGGAATCTTCACAGGAGGGTCCCTGCTCCCTGTCAGGCTCCCACCTTATACCAAACCACCTCTTATGAGCCAGGTTGCCACCAACACTGTCATAgatgaaAATAGTGAGACTGCCAGATCCTGGATTAAGGACCCTCAGACCAACCAGTGGAAGCTGGGAGAACCTCTGGAGCTACGTAGGGCCATGACAGTCATCCATGGGGACAGTAATGGAATGTCAGGAGGGGAAGCTGCTGGAATCACTTTGGGAGTCACCATAGCACTAGGAGTTGTCATTACCTTGGCCATCTATGGTACTCGGAAGTACAAGAAGAAGGAATACCAGGAAATTGAGGAGCAGGAGAGTTTGGTTGGAAGCTTAGCAACAGATGCAACAGTCCTTAATGG |
| V607-K620del | CCCTATGACAAATGCACAGTCATGCCCTGCAGGCTACATCCCACTGAACCTGTTTGAAAGCCTCAAGGTATGTGTGTCCCTGGATTATGAGTTGGGGTTCAAGTTTTCAGTCCCCTTTGGTGGGTTCTTCAGTTGTATAATGGGGAACCCCTTGGTTAATTCTGATACAGCTAAAGACGTCAGAGCACCATCTCTGAAAAAGTGTCCCGGGGGCTTCAGCCAACACCTAGCTGTTATCAGTGATGGATGCCAAGTGTCCTACTGTGTCAAGGCTGGAATCTTCACAGGAGGGTCCCTGCTCCCTGTCAGGCTCCCACCTTATACCAAACCACCTCTTATGAGCCAGGTTGCCACCAACACTGACCCTCAGACCAACCAGTGGAAGCTGGGAGAACCTCTGGAGCTACGTAGGGCCATGACAGTCATCCATGGGGACAGTAATGGAATGTCAGGAGGGGAAGCTGCTGGAATCACTTTGGGAGTCACCATAGCACTAGGAGTTGTCATTACCTTGGCCATCTATGGTACTCGGAAGTACAAGAAGAAGGAATACCAGGAAATTGAGGAGCAGGAGAGTTTGGTTGGAAGCTTAGCAACAGATGCAACAGTCCTTAATGG |
